# Polar-Region Soils as Novel Reservoir of Lactic Acid Bacteria from the Genus *Carnobacterium*

**DOI:** 10.3390/ijms25179444

**Published:** 2024-08-30

**Authors:** Katarzyna Kosiorek, Jakub Grzesiak, Jan Gawor, Agnieszka Sałańska, Tamara Aleksandrzak-Piekarczyk

**Affiliations:** Institute of Biochemistry and Biophysics, Polish Academy of Sciences, 02-106 Warsaw, Poland; k.izdebska@ibb.waw.pl (K.K.); jgrzesiak@ibb.waw.pl (J.G.); gaworj@ibb.waw.pl (J.G.); a.rozga@ibb.waw.pl (A.S.)

**Keywords:** -polar LAB isolates, microbial communities, polar *Carnobacterium* spp., psychrophiles, postglacial environments, *Carnobacterium* plasmids

## Abstract

Polar habitats offer excellent sites to isolate unique bacterial strains due to their diverse physical, geochemical, and biological factors. We hypothesize that the unique environmental conditions of polar regions select for distinct strains of lactic acid bacteria (LAB) with novel biochemical properties. In this study, we characterized ten strains of psychrotrophic LAB isolated from hitherto poorly described sources—High Arctic and maritime Antarctic soils and soil-like materials, including ornithogenic soils, cryoconites, elephant seal colonies, and postglacial moraines. We evaluated the physiological and biochemical properties of the isolates. Based on 16S rRNA and housekeeping genes, the four LAB strains were assigned to three *Carnobacterium* species: *C. alterfunditum*, *C. maltaromaticum*, and *C. jeotgali*. The remaining strains may represent three new species of the *Carnobacterium* genus. All isolates were neutrophilic and halophilic psychrotrophs capable of fermenting various carbohydrates, organic acids, and alcohols. The identified metabolic properties of the isolated *Carnobacterium* strains suggest possible syntrophic interactions with other microorganisms in polar habitats. Some showed antimicrobial activity against food pathogens such as *Listeria monocytogenes* and human pathogens like *Staphylococcus* spp. Several isolates exhibited unique metabolic traits with potential biotechnological applications that could be more effectively exploited under less stringent technological conditions compared to thermophilic LAB strains, such as lower temperatures and reduced nutrient concentrations. Analysis of extrachromosomal genetic elements revealed 13 plasmids ranging from 4.5 to 79.5 kb in five isolates, featuring unique genetic structures and high levels of previously uncharacterized genes. This work is the first comprehensive study of the biochemical properties of both known and new *Carnobacterium* species and enhances our understanding of bacterial communities in harsh and highly selective polar soil ecosystems.

## 1. Introduction

Lactic acid bacteria (LAB) are among the most studied microorganisms used in industry. They are Gram-positive, thermophilic or mesophilic aerotolerant anaerobes, non-sporulating cocci, or bacilli that produce lactic acid as the major fermentation product from various carbohydrates [1]. LAB have gained importance due to their metabolic activity, Generally Regarded as Safe (GRAS) status, and antimicrobial properties, leading to applications as probiotic and protective cultures in food production, pharmaceuticals, and biotechnology. LAB are widely distributed in nature, particularly in nutrient-rich habitats such as raw and fermented foods, decaying plant material, human and animal mucosal cavities, soil, and marine environments [2,3].

Despite extensive research on LAB, polar environments remain poorly characterized regarding these bacteria. Representatives of the genera *Carnobacterium* and *Lactobacillus* have only been identified in Antarctic lake sediments, Arctic permafrost, and Antarctic coastal sandy soils [4,5,6,7]. Little is known about the biology and ecology of psychrotolerant LAB in polar soils and soil-like materials.

The source materials for this study were various soils and soil-associated habitats from King George Island (maritime Antarctica) and Spitsbergen Island (High Arctic). Both islands have similar microbial habitats [8]. Coastal sites frequented by sea birds and marine mammals have developed rich ornithogenic soils due to chemical interactions between animal excreta and underlying rocks [9,10]. Both locations also contain glaciers that have retreated and thinned over the past five decades due to climate warming [11]. As a result, vast deglaciated areas have emerged, mostly devoid of vegetation and prone to erosion [12,13]. Glaciers also harbor soil-like microbial habitats, most notably cryoconite holes—shallow surface melt holes with dark debris at the bottom [14,15]. Although ephemeral, these contribute to periglacial microbial biodiversity [16]. Freshwater lakes are also common in polar regions, often hosting extensive microbial mat biomes [17,18].

Polar-region soils experience extremely harsh conditions, leading to simplified ecosystem structures. Limited organic matter, frequent freeze–thaw and wet–dry cycles, low humidity, low thermal capacity of the substrate, rapid drainage, and strong radiation are detrimental to most terrestrial life forms [8]. While non-ice-covered polar terrains are more abundant in nutrients from marine aerosol, seabirds, and mammalian rookeries [19,20], recently ice-free soils are oligotrophic with limited nutrients [13,21].

The highly selective environments of the High Arctic and maritime Antarctica, influenced by dynamic trophic conditions, can be sources of bacteria with high biotechnological potential. Psychrophilic and psychrotolerant microorganisms are being used as sources of new enzymes of industrial importance, including in pharmaceutical synthesis, production of “green″ chemicals, food processing, and agricultural development [22]. Although there is a general understanding of the microbial community in Arctic and Antarctic soils, most bacteria in these regions belong to the phyla *Acidobacteriota*, *Actinobacteriota*, *Pseudomonadota*, and *Bacteroidota*, while *Bacillota* (parent taxon of LAB) is found in minuscule amounts [23,24]. Therefore, the lack of information on LAB in polar soils is not surprising, and there are also few studies identifying the genetic and phenotypic mechanisms of LAB adaptation to the polar conditions.

This study investigates the genetic and metabolic properties of *Carnobacterium* spp. isolates from Arctic and Antarctic soils and soil-like materials to identify adaptive traits and biotechnological potential. Our results indicate that postglacial polar soils are rich sources of both known and new *Carnobacterium* species with unique characteristics, including the use of uncommon carbon sources, reduced nutritional requirements, resistance to high salt concentrations, growth at low temperatures, as well as a wide pH spectrum, and production of cold-active substances inhibiting other bacteria species. Identifying these properties may facilitate the future use of these isolates in biotechnology applications, many of which can be conducted under more suitable industrial conditions than those involving thermophilic or mesophilic LAB strains.2. Results

## 2. Results

### 2.1. Taxonomy of Carnobacterium spp. Isolates

Ten LAB strains were isolated from two polar regions: nine from terrestrial environments on King George Island in Antarctica and one from Hans Glacier in the Arctic (Table 1). Identification to the genus *Carnobacterium* was determined by 16S rRNA gene sequencing and BLASTn searches. The Arctic isolate 2857 showed 100% sequence identity with *C. maltaromaticum*, while Antarctic strains 2835, 2854, and 2862 aligned with *C. alterfunditum*, *C. jeotgali*, or *C. maltaromaticum*, respectively (Table 1). The remaining isolates had sequence coverage and identity ranges of 93–100% and 98–100%, respectively, relative to various *Carnobacterium* species.

**Figure 1:**
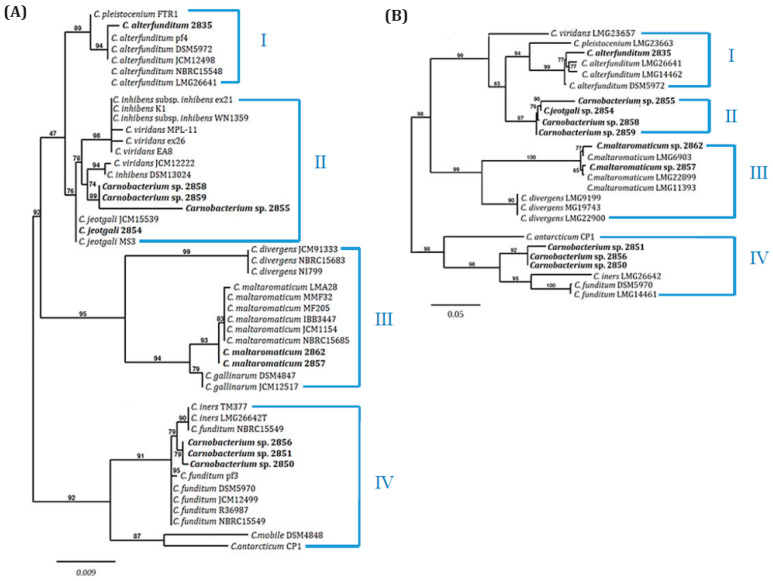
Phylogenetic trees based on 16S rRNA (**A**) and the concatenated *pheS* and *rpoA* genes (**B**). Polar *Carnobacterium* strains isolated in this study are shown in bold; other *Carnobacterium* spp. were retrieved from the GenBank database. Numbers I–IV correspond to the distinguished groups of strain origins: groups I and IV include polar environmental isolates, while groups II and III comprise microbiota from meat, fish, and seafood. The tree was constructed with the neighbor-joining method. Bootstrap values are given at the nodes. The scale bar represents number of substitutions per nucleotide position. *Carnobacterium* strain GenBank accession numbers used in the phylogenetic tree construction: OQ266887, OQ448831, OQ445553, OQ445549, OQ445555, OQ445554, OQ445550, OQ445556, OQ445557, OQ445552, JQLQ01000004, NR104715, LC145583, NR025211, LC145585, AB680898, HE590768, KF317891, NR036895, JX110652, NR025197, KR317896, JX110652, LC145568, JQIV01000006, LC258159, NR116460, LC077075, AB598939, NR102484, GQ304940, AY543032, LC65032, AB680942, NR42093, LC153546, NR108864, HE583595, NR113773, NR025946, LC145584,FR691457, NR113778, HE590756, HE590757, HE590753, HE590754, HE590759, HE590760, HE590768, HE590770, AM694187, AM694188, HE590726, HE590727, HE590729, HE590730, HE590700, HE590701, HE590712, HE590713, HE590706, HE590707, MG734180, MG734181, HE578182, HE578183, HE592670,HE592671, HE590696, HE590715.

High similarity in 16S rDNA sequences across multiple species impeded precise species determination for six isolates, prompting further phylogenetic analysis using a dendrogram based on 16S rDNA sequences from GenBank and study isolates (Figure 1A). Four main groups (I–IV) were distinguished: groups I and IV included polar environmental strains, while groups II and III comprised microbiota from meat, fish, and seafood. Isolate 2835 grouped with *C. alterfunditum* and *C. pleistocenium* from permafrost and polar lake sediments in group I. Group II, including isolates 2854, 2855, 2858, and 2859, contained strains similar to *C. alterfunditum*, *C. inhibens*, *C. jeotgali*, and *C. viridans*, primarily isolated from fish or fermented foods. Among them, isolate 2854 clustered together with *C. jeotgali* strains, while 2855, 2858, and 2859 were in a separate clade with *C. viridans* and *C. inhibens* as the most related species. Group III included *C. divergens*, *C. gallinarum*, and *C*. *maltaromaticum* from meat and seafood microbiome, with isolates 2857 and 2862 clustering with *C. maltaromaticum*. Group IV included isolates 2850, 2851, and 2856, clustering with *C. funditum* and *C. iners* from Antarctic aquatic environments.

A dendrogram based on concatenated housekeeping genes *pheS* and *rpoA* (Figure 1B) showed similar topology to the 16S rRNA tree, with four major groups. However, isolates 2850, 2851, and 2856 remained unassigned to specific species. Isolates 2854, 2855, 2858, and 2859 also formed a separate clade without related known species, potentially due to a lack of deposited *pheS* and *rpoA* genes from *C. jeotgali*.

Further species identification using 16S-23S rRNA spacer regions (ITS) pattern analysis confirmed species affiliations for isolates 2835, 2857, and 2862, while isolates 2850, 2851, 2855, and 2859 exhibited unique ITS patterns, indicating novel species (Supplementary Figure S1). HindIII restriction analysis of ITS fragments corroborated these findings.

### 2.2. Physiological Characteristics

Isolates exhibited typical *Carnobacterium* spp. traits: Gram-positive, non-spore-forming, non-motile facultative anaerobes, occurring in single rods, in pairs, or in small chains. Strains assigned to *C. jeotgali* or *C. maltaromaticum* (2854, 2857, 2862) displayed distinct physiological properties compared to reference mesophilic strains (*C. jeotgali* MS3, *C. maltaromaticum* IBB3447 and LMA28) (Table 2) [28,29,30]. For eight polar isolates, growth temperatures ranged from 4 to 20 °C with an optimum at 16 °C. Notably, two *C. maltaromaticum* strains (2857 and 2862) continued to grow at 37 °C. In contrast, mesophilic strains MS3, IBB3447, and LMA28 had higher temperature optima at 30 °C. Most strains grew in BHI and GM17 media, but not in MRS. Optimal growth for all polar *Carnobacterium* isolates was achieved in 50% BHI at 16 °C. NaCl tolerance ranged from 2 to 8%, with lower salinity resistance observed in mesophilic isolates (MS3, IBB3447, LMA28); however, *C. alterfunditum* 2835 and *C. maltaromaticum* 2857 grew even in 10% NaCl. All isolates demonstrated efficient growth at high pH (8.0–9.0) with a significant lag at pH 5.0–6.0. Mesophilic reference strains *C. maltaromaticum* IBB3447 and LMA28 grew at pH 3.0, indicating a broader pH tolerance compared to polar isolates. Optimal pH levels for all isolates were between 6.0 and 8.0 (Table 2).

### 2.3. Carbon Assimilation

Most strains metabolized several monosaccharides (D-glucose, D-galactose, L-fructose, D-mannose) and disaccharides (D-trehalose, D-cellobiose, sucrose). Significant differences in the ability to utilize individual carbohydrates were noted, particularly in the metabolism of β-glycosides and their derivatives (arbutin, salicin, lactose, amygdalin, and gentiobiose), as well as the α-disaccharide D-melibiose. All strains were metabolically active on several amides (N-acetyl-D-glucosamine, N-acetyl-manno-D-mannosamine) and sugar alcohols (D-mannitol), with limited activity in the presence of amines, amino acids, or organic acids.

**Figure 2:**
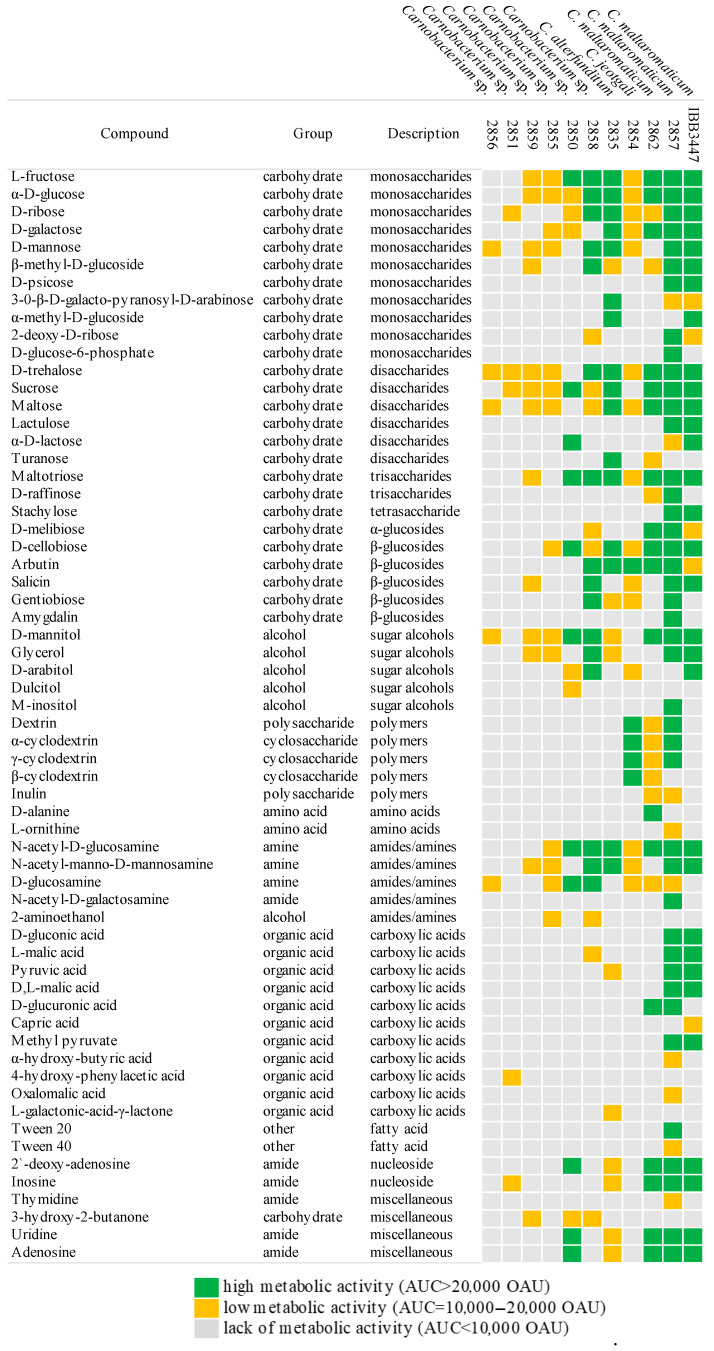
Carbon sources used by *Carnobacterium* spp. strains. The analysis was carried out using Phenotype MicroArrays™ and only those carbon sources are presented for which at least one positive reaction for a given isolate was detected.

*C. jeotgali* 2854 exhibited a metabolic pattern similar to the reference MS3 strain, with minor differences in pentoses and β-glycosides. Polar isolates of *C. maltaromaticum* metabolized the same carbohydrates as their reference strains but with less efficiency than those from milk or cheese. *Carnobacterium* spp. from unassigned species (2851, 2855, 2856, 2859) showed weaker metabolic activity, utilizing only a few pentoses and hexoses (Table 2, Figure 2). Strains such as *Carnobacterium* spp. (2850, 2858), *C. alterfunditum* 2835, and especially *C. maltaromaticum* utilized available carbon sources more efficiently, including di- and polysaccharides (Table 2). Interestingly, *C. alterfunditum* sp. 2835, 2859, 2858, and *C. maltaromaticum* 2857, 2862 could assimilate glycerol as a carbon source (Table 2, Figure 2).

**Figure 3:**
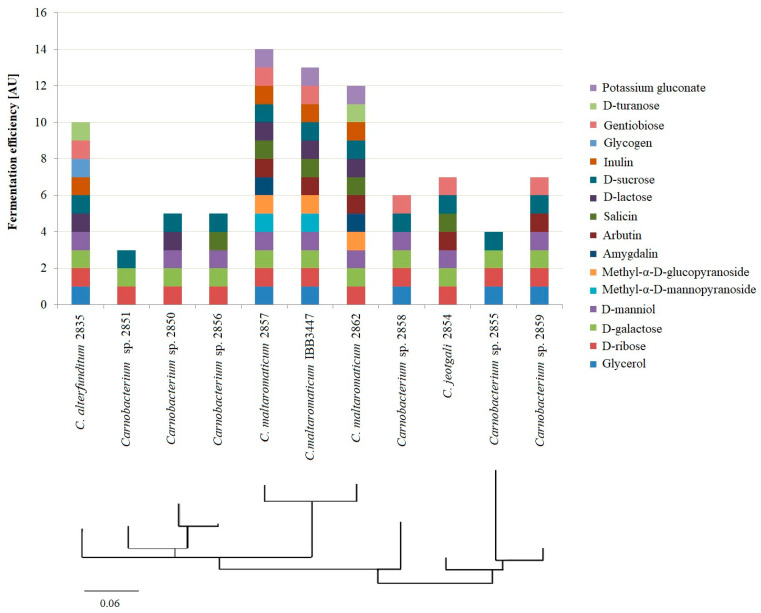
Carbon source assimilation and phylogenetic relatedness between *Carnobacterium* spp. strains. A carbon source assimilation score was considered positive when the overlapping metabolic activity of a strain on a carbon source was observed in both API50CH and Phenotype MicroArrays Biolog^®^ assays. For all strains analyzed, both overlapping and non-metabolized carbohydrates were omitted from the data analysis. Positive results were converted to numerical values and used to build a phylogenetic tree based on the neighbor-joining method as a bootstrap test of phylogeny. In the phylogenetic analysis, the scale bar represents number of substitutions per carbohydrate position in the prepared metabolic patterns of the strains.

Phylogenetic analysis based on combined API50CH and Phenotype MicroArrays (Figure 3) further highlighted similarities and differences in metabolic activity. The strains with the highest metabolic activity (*C. maltaromaticum* 2857 and 2862) clustered with the reference strain *C. maltaromaticum* IBB3447 (Figure 3). *Carnobacterium* sp. 2850, 2851, and 2856 formed a separate cluster, indicating a lack of affinity with known species, while isolates 2855 and 2859 grouped close to *C. jeotgali* 2854, suggesting evolutionary affinity.

### 2.4. Lactic Acid Production

All isolates produced lactic acid (LA) with yields ranging from 4.3 to 5.9 g/L (Table 2). *C. jeotgali* 2854 had the highest average LA titer (5.9 g/L), followed by *Carnobacterium* sp. 2851 (5.7 g/L) and *Carnobacterium* sp. 2859 (5.6 g/L). *C. maltaromaticum* 2862 showed the lowest titer (4.3 g/L). Most strains produced a mixture of L- and D-Las, with the L-LA isoform constituting 96–100% of the total LA produced. *C. maltaromaticum* strains exclusively produced the L form of LA.

### 2.5. Antimicrobial Activity

*Carnobacterium* spp. isolates were tested for antimicrobial activity against 17 pathogenic (*Bacillus* sp., *Campylobacter* sp., *Candida* sp., *Salmonella* sp., *Staphylococcus* spp., *Streptococcus* spp.) and six non-pathogenic indicator strains (*Bacillus* sp., *Lactococcus* sp., and *Staphylococcus* spp.) (Table 3)*. C. jeotgali* 2854 and *Carnobacterium* sp. 2855 exhibited the broadest antimicrobial activity, being bactericidal against seven human pathogens or commensals: *C. jejuni*, *S. aureus*, *S. caprae*, *S. epidermidis*, *S. hyicus*, *S. intermedius*, and *S. lugdunensis*. *Carnobacterium* sp. 2858 was active against four staphylococci species, while *Carnobacterium* sp. 2859 was effective against only *S. epidermidis*. *C. maltaromaticum* 2857 and IBB3447 showed inhibitory activity against *L. monocytogenes* LMGT2604 (Table 3). Bactericidal activity was observed for pure cultures, ammonium sulphate precipitates, and proteinase K-treated supernatants (2854, 2855, 2858, 2859). However, only compounds produced by 2857 and IBB3447 lost bactericidal activity after protease treatment, indicating a bacteriocin-like nature.

### 2.6. Plasmid Characterization

Thirteen plasmids were identified across five *Carnobacterium* spp. isolates: five in *C. maltaromaticum* 2862, three each in 2835 and 2851, and one each in 2856 and 2859 (Table 4). The complete circular nucleotide sequences of these plasmids range from 4.8 to 79.5 kbp with a GC content of 28.9–33.9% (Table 4), aligning with known carnobacterial plasmids but slightly lower that the chromosomal DNA average (38.4%). The plasmids exhibited low sequence similarity (2–58%) to those in GenBank, including plasmids from *Carnobacteriaceae* and *Jeotgalibaca* strains in fish food additives and polar marine sediments (Supplementary Figure S2).

A total of 560 plasmid genes were identified and annotated, with only 35% encoding proteins with known functions, while 65% were of unknown function. Genes encoding membrane and extracellular proteins accounted for 38% and 13%, respectively, with cytoplasmic protein comprising 49%. Detailed gene annotations for each plasmid, including their position and potential functions, are provided in Supplementary Table S1a–m.

Seventeen replicons were identified across the plasmids, with some plasmids containing multiple replicons (Table 4, Supplementary Figure S3). Comparison of these replication proteins with those from other *Carnobacterium* spp. plasmids revealed three phylogenetic groups. Group I includes most Rep proteins (10/17) with Rep_3 (Pfam: PF01051; clade IA) or Rep_trans (Pfam: PF06970; clade IB) domains and a downstream helix-turn-helix (HTH) motif. Group II includes proteins with only the Rep_3 domain (3/17). Group III includes Rep proteins with Rep_3 (clade IIIA) or RepA_N domains (Pfam: PF06970; clade IIIB) and HTH motifs at different positions (Table 4, Supplementary Figure S3).

Mobilization, conjugation, and partition system components were identified in the plasmids (Table 4, Supplementary Table S1a–m). Six plasmids (2851_p2, 2851_p3, 2856_p1, 2859_p1, 2862_p1, and 2862_p4) carry homologs of the mobilization gene mobA, suggesting potential for plasmid mobilization. Conjugal transfer genes (ArdC, TraG, TrwB, YukC) were also identified in some plasmids (2851_p2 and 2856_p1). Partitioning system genes (*parA* and/or *parB*) were present in eight plasmids, indicating stable inheritance mechanisms. Additionally, two plasmids (2835_p1 and 2862_p5) contained complete type II toxin–antitoxin systems (*parD-parE* and *mazE-mazF*), with other plasmids containing partial systems (*phd-yefM* and *txe-yoeB*).

Ten plasmids encoded genes involved in adaptive functions, affecting host cell phenotypes such as carbohydrate or peptide transport and metabolism, and protection against environmental stresses such as low temperatures, high salinity, UV radiation, metal ions, and antibiotics (Supplementary Table S1a–m). Nutrient transport systems included ABC-type peptide transport permeases (OppA, SapC), phosphotransferase system (CelB), and TRAP permeases (DctQ, DctR). Carbohydrate utilization genes encoded components for glucose, galactose, and cellobiose metabolism. Peptidase and protease genes (ArdC, NlpC, PepV, PepA, YddH, Clp) were also identified. Plasmid 2859_p1 included genes for D-glucuronate degradation (UxuA, UxaC).

Four plasmids (2835_p1, 2851_p1, 2851_p3, 2862_p3) contained genes for antibiotic resistance and environmental stress protection. Plasmid 2835_p1 had the highest number of genes related to metal transport and antibiotic resistance, including the *kdpABCD* operon for potassium ion transport and efflux genes (MacB, TcaB). DNA repair genes (*umuD* and *uvrX*) were found in plasmids 2851_p3 and 2851_p1. Plasmid 2862_p3 encoded AmaP, a membrane-anchoring protein for alkaline shock response. Additionally, plasmid 2862_p1 contained *cspD*, a cold shock protein gene with protective functions against low temperatures.

## 3. Discussion

This study presents the genotypic and phenotypic characterization of ten new *Carnobacterium* spp. strains isolated from poorly reported Arctic and Antarctic terrestrial habitats, including cryoconites, elephant seal colonies, and postglacial moraines. Polar-region environments, such as glacial forelands, cryoconite holes, and ornithogenic soils, are recognized for their unique microbiocenosis diversity due to harsh, rapidly changing conditions [13,16,31]. Our findings confirm these speculations, demonstrating that polar-region soils and soil-like materials can be a prolific source of LAB species with unique metabolic traits and antimicrobial properties, facilitating their adaption to polar habitats.

Members of the *Carnobacteriaceae* family have previously been isolated from various habitats, including vacuum-packaged and refrigerated foods, seafood, fish, humans, biofilms, tundra soil, and Antarctic water reservoirs and sediments [6,32,33]. However, there has been no information on their isolation from aerated soils affected by freezing and thawing. DNA sequencing and phylogenic analysis identified strains of *C. jeotgali*, *C. alterfunditum*, and *C. maltaromaticum* in various polar terrestrial structures. Meltwater runoff supplying organic and inorganic matter to soil structures may explain the presence of LAB in postglacial environments. This is supported by phylogenetic clustering of the 16S rRNA genes of the *Carnobacterium* spp. studied here, indicating a close relationship with strains from polar environments. *C. alterfunditum* 2835 and *Carnobacterium* spp. 2850, 2851, and 2856 are closely related to strains isolated from polar environments such as permafrost and Antarctic lake sediments. It has previously been suggested that the process of glacial surface ablation affects the development of cryoconite holes on Antarctic glaciers, and that on ice-covered surfaces, embedded sediments can migrate downward, providing a continuous supply of inorganic and organic material including microorganisms [16,34]. On the other hand, *C. jeotgali* 2854 and *C. maltaromaticum* 2862 showed similarity of 16S rRNA and ITSs to species dominating the microbiota of meat, fish, and seafood. This implies that migrating birds and animals, as well as inorganic and organic components of marine aerosol, should also be considered possible transmitters of microbiota in postglacial habitats. Indeed, Teixeira et al. highlighted the role of marine aerosol, birds, and mammals in the formation of soil microbial communities in coastal regions of the Antarctic environment [20]. Given the phylogenetic affinity of the *Carnobacterium* spp. strains isolated here, it cannot be ruled out that they may be of marine and animal origin. Interestingly, as many as five of the isolated strains may represent new species in the *Carnobacterium* genus, suggesting that polar soils may be an environment in which new LAB species differentiate. However, a definitive determination of the status of the species must follow more rigorous taxonomic characterization.

Polar *Carnobacterium* spp. strains isolated here are characterized by a repertoire of phenotypic traits that result from their adaptation to life in polar-region conditions and that distinguish them from mesophilic carnobacterial strains. Most of these psychrophilic isolates tolerated salinity in the optimal range of 2–6%, which classifies them as moderate halophiles, according to the standard reported for mesophilic LAB [35,36]. However, when it comes to coping with salinity limits, *C. alterfunditum* 2835, *C. jeotgali* 2854, and *C. maltaromaticum* 2857 showed increased tolerance compared to mesophiles, even to values such as 9–10% NaCl. This increased tolerance can be explained by the consequences of ablative processes in the emerging soils, where glacial meltwaters can transport salts from marine aerosol deep into the non-ice-covered area [37]. The presence of sea salt aerosol in Antarctic coastal regions and water evaporation in postglacial environments are also factors that may contribute to the enhanced resistance of the bacteria studied to high salinity [38,39]. The *Carnobacterium* strains presented here grew well in the pH range of 6–9, while one isolate from an Arctic glacier (*C. maltaromaticum* 2857) was able to grow at pH 5 and lower, which can be explained by the pH values of the surface of Hans glacier, where the ice and cryoconites had a pH of 3.3–4.8 [15]. On the other hand, mesophilic carnobacterial strains (*C. maltaromaticum* IBB3447 and LMA28) had a greater resistance to acidification, manifested by their ability to actively grow at pH 3, which may be due to adaptive features to fermentation processes. Mesophilic *Carnobacterium* spp. and other LAB actively acidify the milk or plant material, leading to a reduction in pH values from ca. 7 to below 4 [40]. The soils of the polar region mainly have pH close to neutral, and therefore the discussed differences in resistance to acidification may indicate a loss of adaptive functions towards low pH, resulting from adaptation to polar conditions [24].

LAB are generally fastidious microorganisms with complex nutrient requirements. However, optimal growth conditions for the psychrophiles studied here occurred mainly at reduced nutrient concentrations, while a more rich nutrient solution resulted in ineffective growth or its inhibition. This may be related to the low nutrient content of recently ice-uncovered soil structures or coastal areas where soils remain nutrient-poor despite the influence of organic and inorganic inputs from marine aerosol, local meltwater streams, seabirds, and mammalian rookeries [13]. Such habitats can shape the metabolic properties of the residents and lead to a narrow profile of assimilated compounds. Indeed, simple sugars such as monosaccharides and glucose-containing disaccharides were mainly utilized by isolated *Carnobacterium* spp. strains as carbon sources, while other groups of compounds such as carboxylic acids, amines, amides, and amino acids were assimilated poorly or not at all. This phenomenon can be explained by adaptation to polar environments, where soil structures exposed to freezing and thawing are rich in simple carbohydrates and polyols, whereas complex sugars are not often identified. A preference for simple carbohydrates may also indicate symbiotic interactions with other microorganisms found in these habitats. In polar soil structures, soluble simple sugars are the main source of nutrients, which are provided by algae, mosses, and lichens [41]. Underwood et al. showed that algae under stress conditions can secrete simple carbohydrates that are immediately consumed by bacteria, while mosses and lichens provide disaccharides (mostly sucrose) and several alcohols (such as arabitol or mannitol) [42]. Strains of *Carnobacterium* spp. have been reported in a postglacial microcommunity with a phototrophic partner [41],indicating that symbiotic interactions within this genus may serve as an important adaptive mechanism. Interestingly, three isolated strains (*Carnobacterium* sp. 2858, *C. maltaromaticum* 2857, and *C. alterfunditum* 2835) efficiently metabolized glycerol, which is a unique feature among LAB and could result in potential industrial applications, as this compound is a major waste in biofuel production, and bacteria that utilize it can be used to produce various organic acids (e.g., propionic acid) or in bioremediation processes.

Five carnobacterial isolates examined here carried between one and five plasmids. Such abundance is quite exceptional, as most *Carnobacterium* spp. strains are devoid of such mobile elements [32], and consequently, a limited number of complete carnobacterial plasmid sequences have been deposited in the GenBank database so far. Moreover, plasmids from *Carnobacterium* spp. had a unique structure and low similarity (2–58%) to other bacterial plasmids, the closest of which were pMA1X17-3 of *Jeotgalibaca* sp. MA1X17-3 isolated from polar marine sediment and plasmid 2 of *C. maltaromaticum* 18ISCm from diseased Korean trout [43]. The fact that these five strains were isolated from neighboring sites suggests that the similarities between their plasmids may be due to horizontal gene transfer. Indeed, in support of this hypothesis, in plasmid 2856_p1 of *Carnobacterium* sp. 2856 with the highest homology to pMA1X17-3, the components for a sufficient mobilization system (*mobA*, *trwB*, *yukC*) were identified. The basis for the compatible coexistence of such a multiplicity of distinct plasmids in a single bacterial cell is also worthy of interest. In silico predictions of the affinity of the replication initiation proteins encoded in the 13 plasmids sequenced in this work classified them into three families: Rep3, Rep_trans, and RepA_N. Plasmids encoding Rep proteins from the Rep_3, RepA_N, or Rep_trans families have been shown to replicate via the theta replication mechanism in several lactococcal strains [44,45], suggesting the same mode of replication of carnobacterial plasmids. Phylogenetic analysis of the replication proteins of plasmids from the same strains separated them into distinct groups, indicating a lack of relatedness between them. This may suggest that each *ori* interacts specifically only with the corresponding Rep protein, allowing several different plasmids to coexist in a single bacterial cell. Another characteristic feature of the plasmids identified here is the fairly abundant occurrence of systems for their stable persistence in the cell, both in the form of *parAB* genes responsible for equal partitioning of plasmids into daughter cells and TA systems that eliminate plasmid-deficient cells. Consequently, many newly acquired plasmid-encoded adaptive traits are stably maintained, conditioning bacterial survival in harsh polar environments. Moreover, genetic determinants for adaptation can be actively transmitted to other bacteria due to the presence of mobilization and conjugation transfer genes in some plasmids of the carnobacterial strains identified here.

Plasmid determinants supporting host adaptation to polar environmental conditions are quite widely described in the literature and are distinguished primarily by the presence of proteins with protection functions against cold and UV radiation, including the cold shock protein, the UmuD subunit of DNA polymerase V, and the UV damage repair protein UvrX [46]. The enhanced UV radiation and oxygen solubility present in ice-uncovered polar soils promote the formation of reactive oxygen species (ROS), leading to the risk of damage to cellular macromolecules, including DNA, RNA, proteins, and lipids [12,47]. As such, plasmid genes may play an important adaptive role by providing antioxidant defense mechanisms, including enzymes for DNA repair and reducing levels of toxic ROS. Plasmids identified in polar isolates of *Carnobacterium* spp. encode over 50% of membrane or extracellular proteins, which is quite high compared to other LAB species, such as *L. lactis* IL594 isolated from cheese, which contains only up to 35% of genes encoding such proteins [46,48]. Such a large number of membrane/extracellular proteins encoded in the plasmids may also be related to LAB’s adaptive mechanisms to polar-region environments by adjusting cell membrane fluidity or producing exopolymeric substances that enhance growth during successive freeze–thaw cycles [49]. Two of the identified plasmids (2862_p2 and 2862_p5) carry genes encoding enzymes responsible for lipoprotein and exopolysaccharide (EPS) synthesis and homologous to those of *C. maltaromaticum* LMA28 and *C. funditum* DSM5970, respectively. Previously, it was shown that lipoproteins in polar bacteria determine the maintenance of cell membrane structure and permeability at low temperatures [50], while the formation of EPS can provide a cell coating that enables protection against freezing [51]. This may indicate important plasmid-based mechanism for LAB strains to adapt to polar environments by enhancing the expression of cellular integrity proteins under conditions of low or fluctuating water potential leading to desiccation and freeze–thaw stress in cold habitats.

Identification of plasmid-encoded resistance to macrolides and peptide antibiotics (*macB*, *tcaB* in 2835_p1) represents a novel feature within carnobacterial adaptive traits. As previously reported, the *C. maltaromaticum* strain possesses chromosomally-encoded antibiotic resistance genes, including several aminoglycosides, β-lactams, and tetracyclines [52]. *mac* genes encoding extracellular efflux systems were previously reported to transmit between environmental bacterial species [53], whereas glycopeptide-resistant associated *tca* operon was identified mostly in human pathogenic strains of. *S. aureus* [54]. So far, there is no information on bacterial species with neither *mac* nor *tca* operons in polar environments. Polar regions are still environments with minimal human impact, but human activities at Antarctic research stations can generate macrolide- and aminoglycoside-resistant bacteria [55]. The analyses in this work also showed that Antarctic plasmids are sources of genes that may be beneficial to the metabolic properties of their host. In this group, genes encoding proteins involved in carbohydrate and peptide metabolism are particularly important, since the use of sugars (galactose) and proteins (casein) present in milk affects the exploitation of LAB in the food industry. Indeed, the plasmids described here encoded ABC-type peptide transporters (OppA, SapC), cellobiose-specific permeases CelB of the PTS system [56], as well as mutarotase involved in galactose metabolism (GalM) [57]. Most polar *Carnobacterium* isolates showed moderate or no ability to utilize galactose and lactose compared to mesophilic reference strains isolated from milk and its products. This feature indicates the loss or lack of ability of polar carnobacteria to hydrolyze lactose, the main milk sugar, absent in a polar environment. It is worth mentioning that the plasmidome of polar carnobacteria contains 18% to 100% of genes encoding proteins of unknown function. This is considerably higher compared to the plasmidomes (5–75%) (GenBank, NCBI, last accessed 10 November 2023) of strains isolated from more temperate environments, suggesting their native origin more than transfer from inhabited areas. Therefore, an in-depth analysis of this hitherto undescribed gene pool would be essential for a comprehensive evaluation of LAB plasmidomes from the polar region described in this work.

LAB strains and their metabolites are used industrially as natural preservatives to control the growth of pathogenic and food spoilage bacteria. Unlike thermophilic LAB species, *Carnobacterium* strains isolated from polar environments are capable of thriving and functioning in cold environments, making them particularly suitable for applications that require or benefit from low temperatures. This ability could be especially advantageous for the production of lactic acid and antimicrobial compounds under more favorable technological conditions, including lower temperatures and nutrient concentrations. Several structurally diverse bacteriocins (i.e., bacterial ribosomally synthetized antimicrobial peptides) from *Carnobacterium* spp. with considerable antimicrobial potential have already been characterized [58,59,60], but this feature is not very common among this group of bacteria [61]. The *C. maltaromaticum* 2857 and IBB3447 tested here were active against *L. monocytogenes* with loss of activity after proteinase treatment, strongly suggesting that the compounds they produce may be bacteriocins. Other strains such as *C. jeotgali* 2854 and *Carnobacterium* sp. 2855, 2858, and 2859 showed antagonistic activity against *Staphylococcus* spp., but the active compounds they secreted were resistant to proteolysis, suggesting their non-bacteriocin nature or cyclic structure, which may result in insensitivity to some proteases, as is the case with carnocyclin A [58]. These proteinase-insensitive compounds were active against *C. jejuni*, reinforcing the hypothesis that the antimicrobial compounds produced by strains 2854, 2855, and 2858 are not bacteriocins, as LAB are thought to exhibit anti-*Campylobacter* potential based on the production of non-peptide compounds (e.g., organic acids) [59,62]. This assumption requires further research, as the ability of cold-tolerant and halotolerant LAB strains to produce antimicrobials may be important for their potential industrial applications. Moreover, the production of these compounds appears to be chromosomally encoded, as no plasmids were detected in most of the producer strains, indicating stability in the maintenance of this trait.

LA produced by LAB is an important preservative in food production, also ensuring proper acid and flavor conditions [63]. In comparison to thermophilic LAB species, *Carnobacterium* strains isolated from olar environments possess the ability of lactic acid and antimicrobial compound production in more suitable technological conditions, including lower temperatures and nutrient concentrations. Polar isolates of *Carnobacterium* spp. produced total LA at similar concentrations in the range of 4.3–7.6 g/L, which is not a very efficient titer, but which can be increased by modifying oxygen availability, pH, culture temperature, or nutrient abundance [64]. Significantly, psychrophilic *Carnobacterium* spp. strains produced L(+)-lactic acid as the main end product from glucose, which is more favored in food and pharmaceutical industries, where high purity of LA monomers (≥98%) is critical in the synthesis of polylactides [65]. Furthermore, carnobacteria, by secreting lactic acid into the surrounding soil, can increase the bioavailability of labile phosphorus and biogenic metals such as iron or zinc [66,67]. Therefore, by increasing the availability of essential nutrients, they are likely to contribute to soil biodiversity in polar regions [23,68].

## 4. Materials and Methods

### 4.1. Isolation of Carnobacterium spp. Strains

Soil from polar areas (ornithogenic soils, elephant seal colonies, and postglacial moraines) and soil-like materials (cryoconites and microbial mats from a freshwater reservoir) were collected using sterile spatulas. Cryoconite holes were emptied using a sterile 160 mL plastic syringe. Samples were stored in 50 mL Falcon-type tubes (Fisher Scientific, Waltham, MA, USA) at −20 °C until further study in Poland (see Table 1 for details). Approximately 10 g of each sample was placed in a sterilized 100 mL Simax-type glass bottle with 90 mL of cool (ca. 4 °C) YGLPB semi-strength broth containing peptone (5 g/L), beef extract (4 g/L), glucose (2.5 g/L), lactose (2.5 g/L), yeast extract (1.5 g/L), KH_2_PO_4_ (1.25 g/L), K_2_HPO_4_ (1.25 g/L), MgSO_4_ (0.1 g/L), and MnSO_4_ (0.025 g/L). The semi-closed bottles were incubated anaerobically (7 days; 10 °C; N_2_/CO_2_ atmosphere) in a GenBox 7.0 L container (Biomerieux, Marcy-l′Étoile, France). Cultures (10 μL) were streaked on solid YGLPB medium and incubated (14 days; 10 °C; N_2_/CO_2_). Colonies were transferred to fresh medium and incubated aerobically (7 days; 10 °C). Cultures were checked for catalase activity with 3% H_2_O_2_, Gram’s reaction with KOH, and cell morphology using light microscope with crystal violet staining. Spores were not observed. Isolates were cultured in 50% Brain Heart Infusion (BHI) medium (Oxoid, Basingstoke, UK), solidified with 2% *w*/*v* agar (Merck, Darmstadt, Germany) as needed, and incubated at 16 °C. Reference strain culture conditions are listed in Supplementary Table S2.

### 4.2. Genetic Analyses

Primers used in the study are listed in Supplementary Table S2. The 16S rRNA sequences were amplified as described previously [69]. Amplification of house-keeping genes (*pheS*, *rpoA*) followed [70]. PCR amplifications were performed using Takara ExTaq™ polymerase (TaKaRa, Kusatsu shi, Japan) in a C1000 Thermal Cycler (Bio-Rad, Hercules, CA, USA). PCR product sizes (~1500 bp for 16S rRNA, ~300 bp for *pheS*, ~600 bp for *rpoA*) were confirmed and sequenced using BigDye Terminator v.3.1 chemistry (Applied Biosystems, San Francisco, CA, USA) on an ABI3730xl genetic analyzer (Life Technologies, Carlsbad, CA, USA) at the DNA Sequencing and Synthesis Facility (IBB PAS, Poland). Sequencing reads were assembled using Clone Manager Professional 9 (S&E Software, version 9.2, Edison, NJ, USA) and aligned against GenBank references using BLAST (NCBI, Bethesda, MD, USA) [71]. The 16S rRNA, *rpoA*, and *pheS* gene sequences were deposited in GenBank (accession numbers in Supplementary Table S3).

Phylogenetic trees, based on the neighbor-joining method with bootstrap analysis (1000 repetitions), were prepared using Clustal Omega [72] and Phylogeny.fr [73,74]. Internal transcribed spacer (ITS)-PCR regions were amplified according to [75] using Phusion^®^ High-Fidelity DNA Polymerase (NEB, Hitchin, UK) and KIL1 and GIL1 primers (Supplementary Table S2). ITS-PCR restriction analysis was based on a theoretical restriction analysis of *Carnobacterium* ITS sequences deposited in GenBank [71] and used HindIII, EcoRV, EcoRI, SacI, and BamHI enzymes (Thermo Fisher Scientific, Branchburg, NJ, USA).

### 4.3. Plasmids Isolation and Sequencing

*Carnobacterium* spp. cells were harvested by centrifugation (4 °C; 10 min; 8000 rpm) from a 10 mL culture at mid-log phase (OD_600_ 0.6–0.8). Cell pellets were resuspended in 1 mL of TEG buffer (25 mM Tris pH 8.0, 10 mM EDTA, 50 mM glucose) with lysozyme (10 mg/mL, NEB, UK) and incubated (37 °C; 30 min). Plasmids were isolated using a plasmid isolation kit according to the manufacturer’s instructions (A&A Biotechnology, Poland) and sequenced at the DNA Sequencing and Synthesis Facility of the Institute of Biochemistry and Biophysics of the Polish Academy of Science (IBB PAS, Warsaw, Poland) on an Illumina MiSeq instrument (Illumina, San Diego, CA, USA) using the v3 chemistry kit (Applied Biosystems, San Francisco, CA, USA). Sequences were assembled and annotated using Unicycler v0.4.6. [76] and DFAST 1.2.18 [77]. Regulatory and metabolic gene sequences were compared with NCBI database homologs using BLASTn and BLASTp (NIH, Bethesda, MD, USA; accessed on 2 January 2024). Protein domains were analyzed using PFAM (http://pfam.xfam.org/; accessed on 5 January 2024), CDD (NCBI, NIH), InterPro (EMBL-EBI), and HHpred [78]. Transmembrane helices in proteins were predicted using TMHMM2.0 (DTU Health Tch, Kongens Lyngby, Denmark). The complete nucleotide sequences of the *Carnobacterium* spp. plasmids were deposited in GeneBank (accession numbers in Table 4).

Optimal growth conditions were tested as described previously [32]. *Carnobacterium* spp. strains were inoculated from deep-freeze in YGLPB, incubated (14 days; 10 °C), transferred to the desired media (50% BHI, BHI, LB, MRS), and incubated at various temperatures (4 °C, 16 °C, 20 °C, 30 °C, 37 °C) for 72 h under aerobic conditions. Optical density (OD_600_) was measured using a plate reader (Bioscreen C, Helsinki, Finland). Salinity tolerance was assessed in 50% BHI with 2%, 4%, 6%, 8%, 9%, or 10% NaCl (Merck, Germany). pH tolerance was tested in 50% BHI at 5.0, 6.0, 7.0, 8.0, or 9.0, adjusted with H_2_SO_4_ or NaOH. Single colonies were transferred to 50% BHI and incubated (12 h; 16 °C), and 2 μL of each culture was added to 100-well plates with 198 μL of medium at desired salinity or pH. OD_600_ was measured using a plate reader (Bioscreen C, Finland) for 72 h at 16 °C. Experiments were performed in triplicate.

### 4.4. Metabolic Analyses

Carbon source fermentation patterns were determined using API50CHL (BioMérieux, France) and Phenotype MicroArrays (Biolog, Hayward, CA, USA). Colonies were scraped from 50% BHI agar plates, titrated in IF-0a fluid (Biolog, USA) to 65% transmittance, and supplemented with growth supplements and Biolog redox tetrazolium G dye (Biolog, USA), according to standard protocols recommended by Biolog for *Streptococcus* species. Then, 100 μL aliquots were added to PM1 and PM2 plates and incubated in an OmniLog incubator-reader for 72 h at 16 °C and 30 °C for polar and dairy isolates, respectively. OmniLog arbitrary units (OAUs) were recorded and areas under the curve (AUC) of metabolic activity were calculated and averaged and presented in OAUs.

Swimming and swarming assays were performed as described by [79]. Strains were streaked on 50% BHI agar plates and incubated (48 h; 16 °C), and single colonies were transferred to swimming and swarming plates. Results were recorded after 24 and 48 h.

Lactic acid (LA) detection followed [80]. Total LA and concentrations of D- and L-lactate were assayed in triplicate using a D-/L-lactic acid determination kit (Megazyme International, Wicklow, Ireland).

### 4.5. Antimicrobial Activity and Bacteriocin Purification

Antimicrobial activity of overnight cultures and ammonium sulfate precipitates of post-culture liquids was tested against selected Gram-positive and Gram-negative bacteria using the spot-on-lawn method [81]. Crude extracts were treated with proteinase K (NEB, USA) at 1 mg/mL for 1 h to test susceptibility to proteolysis. Bacteriocins were precipitated with ammonium sulphate as described previously [82] and stored at −20 °C.

### 4.6. Statistical Analysis

Data on genetic and physiological traits of isolated *Carnobacterium* spp. strains were regressed to estimate a 95% confidence level (*p*-value ≤ 0.05) using Microsoft Excel (Excel 2021 for Windows, Microsoft). Experiments were conducted in triplicate unless otherwise specified.

## 5. Conclusions

The study presented here provides the first in-depth insight into the characterization of *Carnobacterium* spp. isolated from less explored niches—soils and soil-like materials of the Arctic and Antarctic regions—sources of LAB that have not been previously reported in the scientific literature. Performed analyses provide:

1. Identification of metabolic properties of isolated *Carnobacterium* strains that may suggest an ecological role involving various syntrophic interactions with other microorganisms in polar habitats, such as algae, mosses, and lichens, through the secretion and fermentation of carbohydrates and alcohols.
2. Presentation of methodology and data providing guidelines for the isolation of psychrophilic LAB from environmental resources of polar regions; identification of new *Carnobacterium* species; and characterization of metabolic traits and antimicrobial properties that determine the adaptive potential of LAB strains to permanently cold habitats, as well as for biotechnological application.
3. Description of important metabolic features of industrial importance in cold-adapted *Carnobacterium* strains, such as the production of fermented foods, probiotics, and antimicrobial compounds, that could be more efficiently harnessed under conditions that are less demanding than those required by thermophilic LAB strains.
4. Detailed characterization of carnobacterial plasmids and identification of certain genetic elements that may be useful in targeted engineering of suitable *Carnobacteriaceae* strains.

The findings underscore the under-explored biodiversity of polar regions and their potential for biotechnological applications.

## Figures and Tables

**Table 1:** Isolation source and taxonomic identification of polar *Carnobacterium* spp. isolates based on 16S rRNA.

Isolate	Source of Isolation	Longitude	Latitude	Alignment Result ^1^	Query Coverage [%]	Sequence Identity [%]	Taxonomic Identification	Reference for Soil Components
2835	Cryoconite, Ecology Glacier, King George Island (Antarctica)	−58.47559	−62.17331	*C. alterfunditum* JCM12498	100	100	*C. alterfunditum*	[15]
2850	Ornithogenic soil, Llano Cape, King George Island (Antarctica)	−58.44761	−62.17491	*C. funditum* NBRC15549 *C. funditum* JCM12499 *C. iners* TM377	100 100 100	100 99 99	*Carnobacterium* sp.	[25]
2851	Ornithogenic soil, Arctowski Station terrain, King George Island (Antarctica)	−58.45869	−62.16317	*C. funditum NBRC15549* *C. funditum* JCM12499 *C. funditum* pf3 *C. iners* TM377	100 99 99 99	100 99 99 99	*Carnobacterium* sp.	[23]
2854	Soil, Baranowski Glacier foreland, King George Island (Antarctica)	−58.44339	−62.20501	*C. jeotgali* MS3 *C. jeotgali* JM15539	100 100	100 99	*C. jeotgali*	Not available
2855		−58.44084	−62.2047	*C. jeotgali* MS3 *C. inhibens* WN1359 *C. funditum* NBRC15549	94 93	98 98	*Carnobacterium* sp.	
2856	Soil, seal elephants wake, King George Island (Antarctica)	−58.46284	−62.16255	*C. funditum* NBRC15549 *C. funditum* JCM12499 *C. iners* TM377	100 99 99	100 99 98	*Carnobacterium* sp.	[26]
2857	Soil, Hans Glacier foreland, Spitsbergen (Arctic)	15.597403	77.015244	*C. maltaromaticum* JCM1154	100	100	*C. maltaromaticum*	[27]
2858	Soil, Windy Glacier foreland, King George Island (Antarctica)	−58.47487	−62.2309	*C. jeotgali* MS3 *C. inhibens* WN1359 *C. viridans* JCM12222	100 100 99	100 100 99	*Carnobacterium* sp.	Not available
2859		−58.47257	−62.23316	*C. jeotgali* JCM15539 *C. inhibens* WN1359 *C. jeotgali* MS3	100 100 100	100 100 99	*Carnobacterium* sp.	
2862	Freshwater microbial mats, Jasnorzewski Garden, King George Island (Antarctica)	−58.4683	−62.15943	*C. maltaromaticum* JCM 1154	100	99	*C. maltaromaticum*	Not available

^1^ Accession numbers from the GenBank database (NCBI, UK) of strains identified in the alignment search are shown in the description of Figure 1.

**Table 2:** Biochemical and physiological features of polar and mesophilic *Carnobacterium* spp. strains. +, very good growth or positive reaction; +/−, weak growth or reaction; −, no growth or negative reaction; N/A, not available; N/D, not done. Numbers shown in parentheses refer to the optimal growth conditions of the identified strain.

Feature	*C. alterfunditum*		*C. jeotgali*		*C. funditum*	*Carnobacterium* sp.						*C. maltaromaticum*			
	2835	DSM5972	2854	MS3 ^1^	DSM5970	2850	2851	2855	2856	2858	2859	2857	2862	IBB3447	LMA 28 ^2^
Motility	−	−	−	−	−	−	−	−	−	−	−	−	−	−	−
Growth:															
50% BHI	+	+/−	+	N/A	+/−	+	+	+	+	+	+	+	+	+/−	N/A
100% BHI	+/−	−	+/−	N/A	−	+/−	+/−	+/−	+	+/−	+/−	+	+	+	N/A
200% BHI	+/−	−	−	N/A	−	+/−	+/−	−	+/−	−	−	+	+	+	N/A
LB	+/−	+/−	+/−	N/A	+/−	+/−	+/−	+/−	+/−	+/−	+/−	+/−	+/−	+/−	N/A
GM17	+/−	−	+/−	N/A	−	+/−	+/−	+/−	+/−	+/−	+/−	+	+	+	N/A
MRS	−	−	−	N/A	−	−	−	−	−	−	−	−	−	+	N/A
Temp range [°C]	4–20 (16)	4–12 (4)	4–20 (16)	4–37 (30)	4–20 (16)	4–20 (16)	4–20 (16)	4–16 (16)	4–30 (16)	4–20 (16)	4–20 (16)	4–37 (16)	4–37 (16)	16–37 (30)	16–37 (30)
NaCl tolerance [%]	2–10 (8)	2–6 (3)	2–9 (7)	0–5 (2)	2–10 (6)	2–6 (4)	2–6 (4)	2–9 (4)	2–8 (6)	2–6 (4)	2–8 (7)	2–10 (8)	2–8 (6)	2–8 (3)	2–8 (3)
pH range	6–9 (8)	6–8 (7)	6–9 (7)	5.5–9 (8.5)	6–9 (7)	6–9 (8)	6–9 (8)	6–9 (7)	6–9 (7)	6–9 (7)	6–9 (7)	5–9 (6)	5–9 (6)	3–9 (6)	3–9 (6)
Produced acid from:															
Glycerol	+	+/−	−	−	−	−	−	−	−	−	−	+	+/−	+	+
D-ribose	+	+	+/−	−	+	−	+/−	−	+	−	−	+	+	+	+
D-galactose	+	+/−	+/−	−	+	+/−	+	+/−	+	+/−	+/−	+	+/−	+/−	+/−
D-glucose	+	+	+/−	+	+	+/−	+	+/−	+	+	+/−	+	+	+	+
D-fructose	+	+/−	+/−	+	+	+/−	+	+/−	+	+	+/−	+	+	+	+
D-mannose	+	+/−	+/−	+/−	+	+/−	+	+/−	+	+	+/−	+	+	+	+
D-sucrose	+/−	+/−	+/−	+/−	+	+/−	+/−	+/−	+	+/−	+/−	+	+	+	+
D-mannitol	−	−	+/−	+	−	−	−	+/−	−	+/−	+/−	+	+/−	+	+
D-melibiose	−	−	−	−	−	−	−	−	−	−	−	+	+/−	+/−	−
D-cellobiose	+	−	+/−	−	−	+	−	+/−	−	+/−	−	+	+	+	+
Arbutin	−	N/D	+/−	+/−	−	−	−	−	−	+/−	+/−	+	+	+	+
Salicin	+	+	+/−	N/A	−	−	−	+/−	−	+/−	+/−	+	+	+	+
D-lactose	+	−	−	−	−	+/−	−	+/−	−	+/−	−	+	−	+	+
Amygdalin	−	+/−	+/−	−	−	−	−	−	−	+/−	+/−	+	+	+	+
Gentiobiose	+/−	N/D	−	−	−	−	−	−	−	+/−	+/−	+	+/−	+	+
Inulin	+/−	−	−	−	−	−	−	−	−	−	−	+/−	−	−	−
Starch	−	−	−	−	−	−	−	−	−	−	−	+/−	−	−	−
Glycogen	+/−	−	−	−	−	−	−	−	−	−	−	−	−	−	−
Esculin	−	−	+	+	+	+	+	+	+	+	+	+	+	+	+
Produced lactic acid [g/L]	5.5	3.9	5.9	N/A	3.6	5.5	5.7	5.3	5.6	5.8	5.5	4.5	4.3	5.3	NA
L-lactic acid [%]	98	99	98	N/A	98	98	96	98	96	98	97	100	100	100	NA
D-lactic acid [%]	2	1	2	N/A	2	2	4	2	4	2	3	0	0	0	NA

**^1^**, data are from [28]; ^2^, data are from [28,29,30].

**Table 3:** Spectrum of antimicrobial activity of *Carnobacterium* strains against the most common human pathogens. Black circle corresponds to strong antimicrobial activity; white circle—no microbial activity.

Strain	*C. alterfunditum*		*C. funditum*	*Carnobacterium* sp.						*C. jeotgali*	*C. maltaromaticum*		
	2835	DSM5972	DSM5970	2850	2851	2856	2855	2858	2859	2854	2857	2862	IBB3447
*B. cereus* IBB3390	○	○	○	○	○	○	○	○	○	○	○	○	○
*B. subtilis* 168	○	○	○	○	○	○	○	○	○	○	○	○	●
*C. jejuni* 81176	○	○	○	○	○	○	●	●	○	●	○	○	○
*C. albicans* CAI-4	○	○	○	○	○	○	○	○	○	○	○	○	○
*L. monocytogenes* LMGT2604	○	○	○	○	○	○	○	○	○	○	●	○	●
*P. aeruginosa* ATCC9027	○	○	○	○	○	○	○	○	○	○	○	○	○
*S. typhimurium* TT622	○	○	○	○	○	○	○	○	○	○	○	○	○
*S. aureus* ATCC638	○	○	○	○	○	○	●	●	○	●	○	○	○
*S. caprae* DSM20608	○	○	○	○	○	○	●	●	○	●	○	○	○
*S. delphini* DSM20771	○	○	○	○	○	○	○	○	○	○	○	○	○
*S. epidermidis* DSM20044	○	○	○	○	○	○	●	●	●	●	○	○	○
*S. hyicus* DSM20454	○	○	○	○	○	○	●	○	○	●	○	○	●
*S. intermedius* DSM20373	○	○	○	○	○	○	●	○	○	●	○	○	○
*S. lugdunensis* DSM4804	○	○	○	○	○	○	●	●	○	●	○	○	○
*S. pseudintermedius* DSM21284	○	○	○	○	○	○	○	○	○	○	○	○	○
*S. saprophyticus* DSM20229	○	○	○	○	○	○	○	○	○	○	○	○	○
*S. schleiferi* DSM6628	○	○	○	○	○	○	○	○	○	○	○	○	○
*S. agalactiae* IBB130	○	○	○	○	○	○	○	○	○	○	○	○	○
*S.mitis* IBB3449	○	○	○	○	○	○	○	○	○	○	○	○	○
*S. pneumoniae* 5005	○	○	○	○	○	○	○	○	○	○	○	○	○
*S. agalactiae* IBB123	○	○	○	○	○	○	○	○	○	○	○	○	○
*S. sobrinus* IBB3450	○	○	○	○	○	○	○	○	○	○	○	○	○
*L. lactis* 1403	○	○	○	○	○	○	○	○	○	○	○	○	○

**Table 4:** General features of *Carnobacterium* spp. plasmids identified in this study.

Species	Strain	Plasmid	GenBank Acc. No.	Size [bp]	GC Content [%]	Replication (Rep Protein Family)	Mobility	Maintenance		Genes of Adaptation Mechanisms			
							Transfer and Mobilization Genes	Plasmid addiction Genes	Stability Genes	Carbohydrate Metabolism Genes	Peptide Metabolism Genes	DNA Repair Genes	Cell Resistance Genes
*C. alterfunditum*	2835	2835_p1	OR786483	37,450	30.3	RepB (HTH_11)	none	*parD* *parE*	*parA*	*celB* *pdaC*	*ardC*, *oppA* *pepV*		*kdp* operon *macB*, *tcaB*
		2835_p2	OR786484	4480	33.7	RepB (Rep_3)	none	none	none				
		2835_p3	OR786485	7305	29.1	RepB (Rep_3) Rep (HTH_11)	none	none	none	*lacX*			
*Carnobacterium* spp.	2851	2851_p1	OR786482	3360	28.9	RepB (Rep_trans)	none	none	none	*galM*		*uvrX*	
		2851_p2	OR786486	45,450	28.4	RepA (HTH)	*mobP*, *traG* *ardC*	*parE*	*parA*		*nlpC*		
		2851_p3	OR786487	37,450	30.3	RepB (Rep_3)	*mobA*	none	*parA*	*pdaC*		*umuD*	*kdp* operon
	2856	2856_p1	OR786488	23,480	28.9	RepB (Rep_3)	*mobA*, *trwB* *yukC*	none	*parA* *parB*		*yddH*		
	2859	2859_p1	OR786489	36,820	31.1	RepB (Rep_3)	*mobA*	none	*parA*	*uxa*, *dctQ* *kdg* operon			
*C. maltaromaticum*	2862	2862_p1	OR786490	10,598	33.4	RepB (Rep_3) Rep (HTH_XRE)	none	none	none		*pepA*		
		2862_p2	OR786491	43,406	35.5	Rep (HTH)	none	none	none		*clp*		
		2862_p3	OR786492	60,935	33.2	RepB (HTH_11) Rep (HTH_XRE)	none	*mazF*	*parA* *parB*	*celB*			*amaP*
		2862_p4	OR786493	66,284	33.4	RepB (HTH_11) Rep (HTH_XRE)	*mobA* *traC* *traG*	*parE* *mazF* *yoeB*	*parA*				
		2862_p5	OR786494	79,510	32.4	RepB (Rep_3)	none	*mazE* *mazF* *yoeB*	*parA* *parB*	*galE* *ugd* *wecB*	*yjiD*		

## Data Availability

The complete nucleotide sequences of the *Carnobacterium* spp. plasmids obtained in this work were deposited in GenBank under accession numbers OR786482 to OR786494. The nucleotide sequences of the 16S rRNA, *rpoA*, and *pheS* genes were deposited under accession numbers OQ266887, OQ445549 to OQ445559, and OQ865238 to OQ865259.

## Supplementary Materials

The following supporting information can be downloaded at: https://www.mdpi.com/article/10.3390/ijms25179444/s1.
