# Supplementary material for: Polar-Region Soils as Novel Reservoir of Lactic Acid Bacteria from the Genus Carnobacterium
Source: Int J Mol Sci. 2024 Aug 30;25(17):9444. doi: 10.3390/ijms25179444 (PMC11395011; doi:10.3390/ijms25179444)
Supplement: Supplementary file 1 [file ijms-25-09444-s001.zip › ijms-3125193-supplementary.pdf]

Polar Region Soils as Novel Reservoir of Lactic Acid Bacteria from the Genus *Carnobacterium*

Katarzyna Kosiorek, Jakub Grzesiak, Jan Gawor, Agnieszka Sałańska and Tamara Aleksandrzak-Piekarczyk *

Institute of Biochemistry and Biophysics, Polish Academy of Sciences, Warsaw, Poland
* Correspondence: author. Mailing address: IBB PAS, Pawińskiego 5a, 02-106 Warsaw, Poland, Phone: +48 22 592 1213, E-mail: tamara@ibb.waw.pl.

Supplementary Materials

Figure S1. ITS-PCR and ITS-RFLP profiles of *Carnobacterium* spp. strains. Both polar *Carnobacterium* sp. isolates (2835, 2850, 2851,2854, 2855, 2856, 2857, 2858, 2859, 2862) and several reference strains (*C. alterfunditum* DSM5972 [30], *C. funditum* DSM5970 [30], *C. maltaromaticum* IBB3447) were used in analysis in order to evaluate genetic relatedness between carnobacteria. ITS-RFLP patterns were distinguished on restriction analysis by HindIII endonuclease, numbers in bold reference to DNA fragments with high intensity shown in agarose gel electrophoresis.

ITS pattern

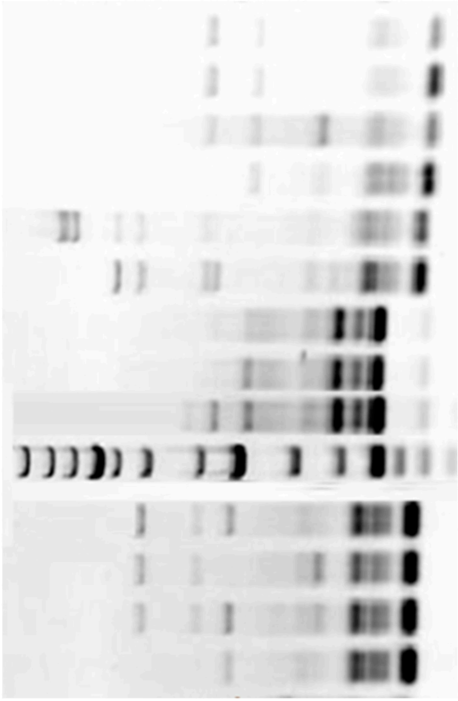

ITS- RFLP pattern

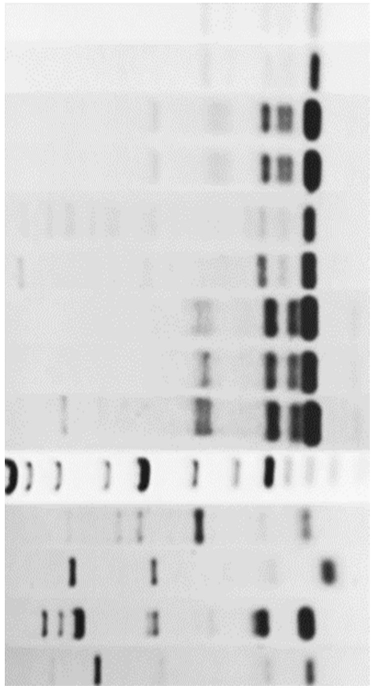

Strain		HindIII fragment size (bp)
Carnobacterium sp.	2850	950, 900, 800, 500, 400, 300
Carnobacterium sp.	2851	950, 900, 800, 500, 400, **300**
Carnobacterium sp.	2856	1500, 900, 500, 400, **300**
C. funditum	DSM5970	1500, 900, 500, 400, **300**
C. alterfunditum	2835	4000, 3200, 3000, 2500, 2000, 1500, 1400, 500, 400, **300**
C. alterfunditum	DSM5972	4000, 3200, 3000, 2500, 2000, 1500, 1400, **500**, 400, **300**
C. maltaromaticum	2857	1000, 900, **500**, **400**, **300**
C. maltaromaticum	2862	900, **500**, **400**, **300**
C. maltaromaticum	IBB3447	3000, 900, **500**, **400**, **300**
**GeneRuller 1 kb Plus ® Ladder**		**GeneRuller 1 kb Plus ® Ladder**
Carnobacterium sp.	2854	2900, 1700, 1000, 550, 300
Carnobacterium sp.	2855	**2900**, 1400, 500, 200
Carnobacterium sp.	2858	3500, 3000, **2800**, 1500, 1400, 1000, **500**, **300**
Carnobacterium sp.	2859	3200, **2300**, 1400, 550, **300**

Figure S2. DNA sequence similarity between *Carnobacterium* spp. plasmids derived from this work and other plasmids from the NCBI database. Plasmid sequences are placed around a circle, where plasmid names are shown on the sides of each plasmid. Ribbons represent local alignments in five colours representing the four quartiles up to the maximum score (blue to red). Ribbons may reverse if the local alignments are inverted. Plasmids used in analysis: pCAR50 from *Carnobacterium* sp. 17-4; pA1P3, pA1P4 from *Carnobacterium* sp. ANT_1; pCP1 from *Carnobacterium* sp. CP1; p124 from *B. subtilis* PN51; pMA1X17-3 from *Jeotgalibaca* sp. MA1X17-3; p181SCm_p1, p181SCm_p2 from *C. maltaromaticum* 181SCm. GenBank accession numbers used in the plasmids sequence similarity analyses: NC_015390.1; NZ_MN657137.1; NZ_CP010819.1; NZ_MN657138.1; NZ_CP036020.1; NZ_CP090984.1; AJ518865.1; NZ_CP045041.1; NZ_CP045042.1; NZ_CP045043.1; NZ_CP045044.1.

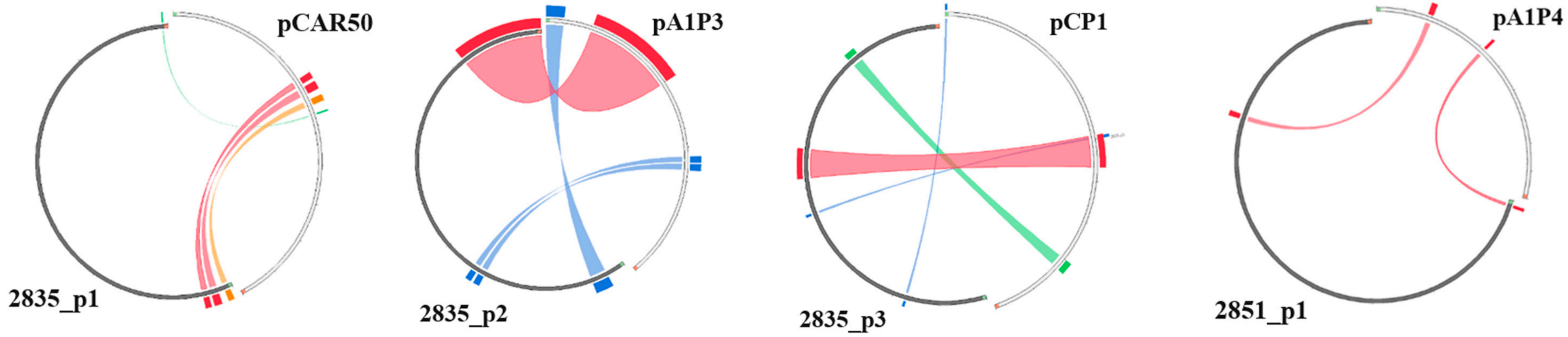

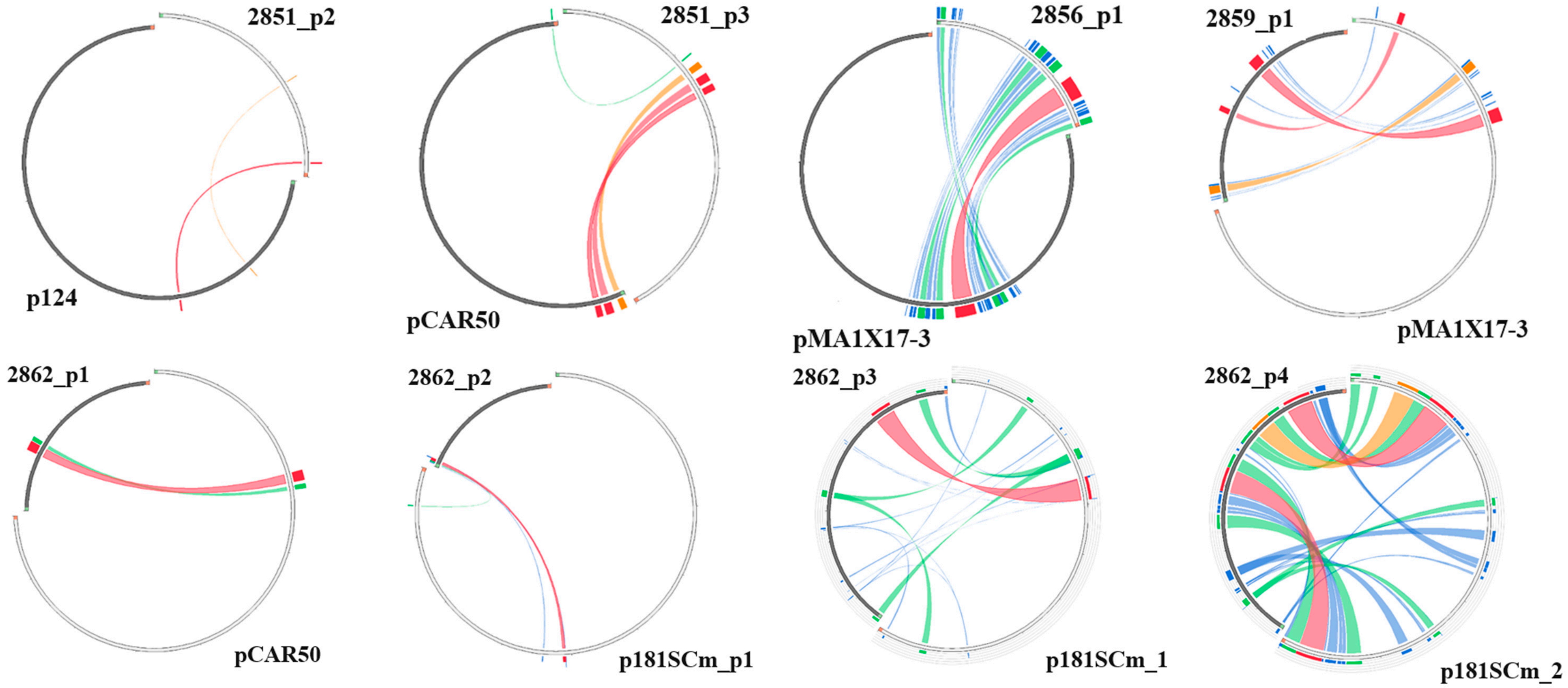
2851_p2
p124
2851_p3
pCAR50
2856_p1
pMA1X17-3
2859_p1
pMA1X17-3
2862_p1
pCAR50
2862_p2
p181SCm_p1
2862_p3
p181SCm_1
2862_p4
p181SCm_2

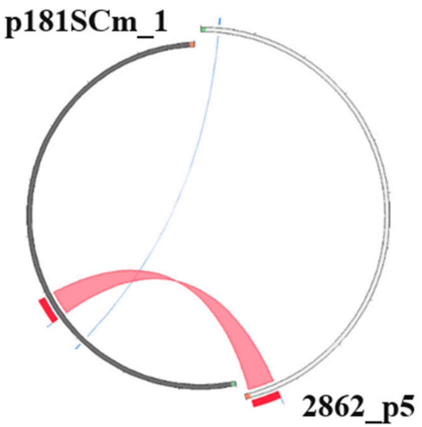
p181SCm_1
2862_p5

Figure S3. Phylogenetic tree based on plasmid replication initiation proteins sequences from *Carnobacterium* spp. strains. Rep, RepA and RepB sequences from polar *Carnobacterium* spp. strains isolated in this study are shown in bold, other were retrieved from the GenBank database. The tree was constructed with the neighbour-joining method. Bootstrap values are given at the nodes. The scale bar represents number of substitutions per nucleotide position. The right panel shows the arrangement of domains identified in the sequences of replication proteins in the group. *Carnobacterium* spp. strains GenBank accession numbers used in phylogenetic tree construction: NC_015390.1; NZ_MN657136.1; NZ_MN657135.1; NZ_MN657138.1; NC_022601.1; NC_022603.1; NZ_CP010819.1; NZ_MN657136.1; NZ_MN657137.1; NZ_CP016847.1; NZ_CP084917.1; NZ_CP084919.1; NZ_CP016848.1; NZ_MN657137.1; NZ_MN657135.1; NC_022601.1; NZ_CP084919.1; CP084920.1.

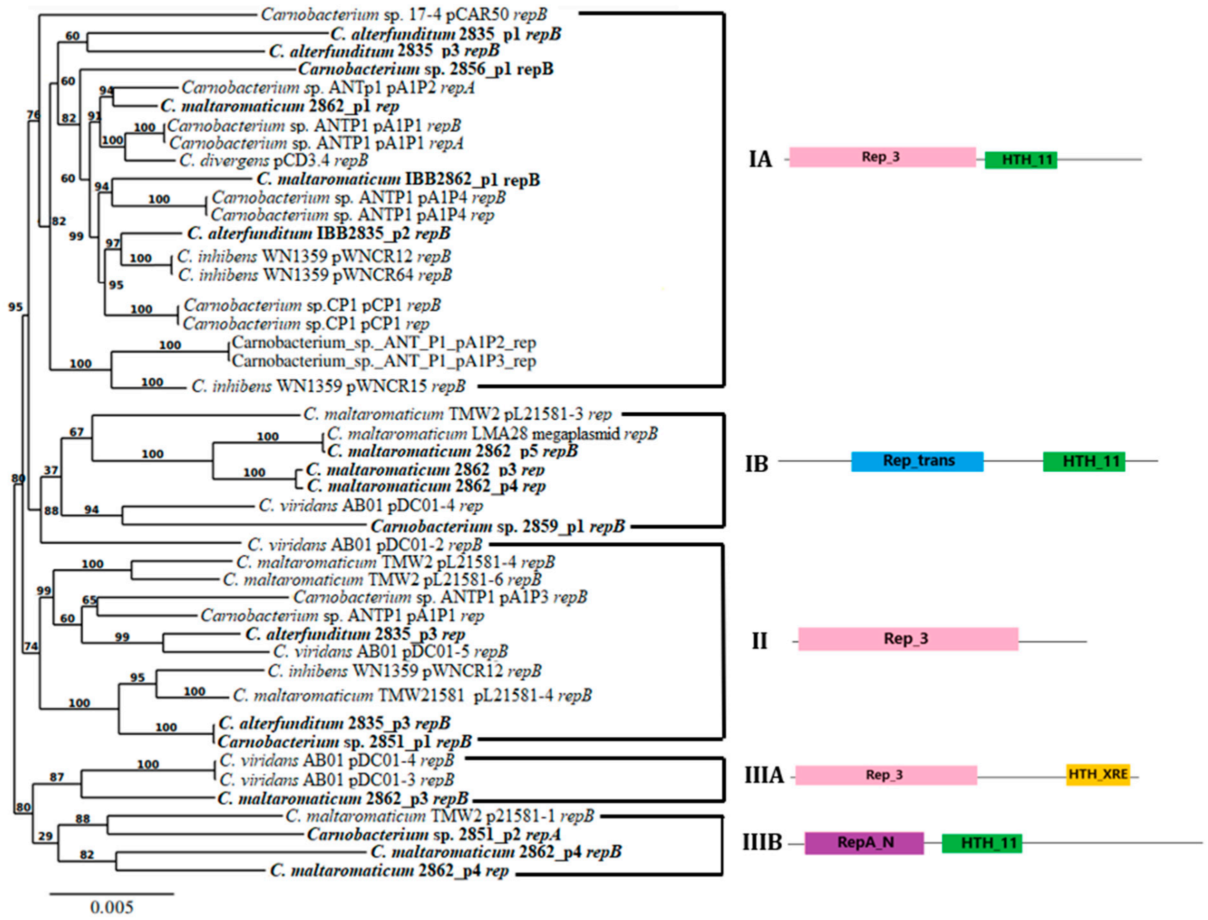

S1a. Annotation of the 2835_p1 plasmid of *C. alterfunditum* 2835

Tag	Gene	Position		Function	BLASTp best result	Identity	Coverage	Localization *	Protein domain	Protein domain function
		start	stop			(%)	(%)			
2835_p1_1	*repB*	2	1387	replication protein RepB	recombination protein [*Carnobacterium iners* TM377]	100	73	C	HTH_11	DNA binding domain
2835_p1_2	*orf2*	1303	1629	hypothetical protein	hypothetical protein [*Carnobacterium* sp. 17-4]	100	74	C	transpos_ISL3 super family	ISL3 family transposase
2835_p1_3	*ytqB*	1927	2175	transposase	transposase [*Carnobacterium* sp. 17-4]	100	77	C	transpos_ISL3 super family	ISL3 family transposase
2835_p1_4	*dltD*	2142	2438	D-alanine-D-alanyl carrier protein ligase	D-alanine-l carrier protein ligase [*Bacillus piscicola* NZ89]	99	61	M	CE4_SF super family; DUF3298	Catalytic NodB domain of the carbohydrate esterase 4 superfamily
2835_p1_5	*pdaC*	2705	4162	polysaccharide deacetylase	polysaccharide deacetylase [*Carnobacterium iners* TM377]	62	75	C	Aldose_epim 1	Aldose_epim 1
2835_p1_6	*orf6*	4333	4869	hypothetical protein	hypothetical protein [*Carnobacterium iners* LMG 26642T]	98	79	M	KdpC super family	K+-transporting ATPase, c chain;
2835_p1_7	*kdpC*	5173	5805	potassium-transporting ATPase KdpC subunit	potassium-transporting ATPase subunit KdpB [*Carnobacterium iners* LMG 26642T]	100	93	M	P-type_ATPase_K	potassium-transporting ATPase ATP-binding subunit, KdpB
2835_p1_8	*kdpB*	5880	7934	K-transporting ATPase ATP-binding subunit	potassium-transporting ATPase subunit KdpA [*Carnobacterium iners* LMG 26642T]	100	92	M	KdpA super family	Potassium-transporting ATPase A subunit;
2835_p1_9	*kdpA*	7948	9666	K-transporting ATPase K-binding subunit	response regulator transcription factor [*Carnobacterium iners* LMG 26642T]	100	92	M	OmpR	DNA-binding response regulator, OmpR family
2835_p1_10	*kdpD*	10050	10760	DNA-binding response regulator	DNA-binding response regulator KdpD [*Carnobacterium iners* LMG 26642T]	100	85	C	KdpD	K+-sensing histidine kinase KdpD involved in signal transduction
2835_p1_11	*orf11*	10753	13425	recombinase	recombinase family protein [*Carnobacterium inhibens* WN1359]	98	93	C	SR_ResInv	Serine Recombinase family, Resolvase and Invertase subfamily
2835_p1_12	*ficA*	13938	14534	recombinase family protein	Fic family protein [*Carnobacterium* sp. 17-4]	100	85	C	COG3177 super family	Fic family protein involved in transcription

* Abbreviations used in protein localization prediction: C- cytoplasm; M- membrane, E- extracellular

S1a. Annotation of the 2835_p1 plasmid of *C. alterfunditum* strain 2835 (continued)

Tag	Gene	Position start	Position stop	Function	BLASTp best result	Identity (%)	Coverage (%)	Localization	Protein domain	Protein domain function
2835_p1_25	*orf25*	26654	27352	hypothetical protein	hypothetical protein [*Carnobacterium funditum* DSM5970]	98	61	E	PRK15111 super family	peptide ABC transporter permease SapC
2835_p1_26	*oppA*	27480	28424	peptide ABC transporter permease	peptide ABC transporter permease [*Carnobacterium jeotgali*]	100	87	M	DppC super family	ABC-type di/oligopeptide/nickel transport system, permease component
2835_p1_27	*orf27*	28850	29158	hypothetical protein	hypothetical protein [*Carnobacterium mobile* S2]	98	75	M	no data	no data
2835_p1_28	*orf28*	29646	29801	hypothetical protein	hypothetical protein [*Carnobacterium* sp. 17-4]	100	79	M	no data	no data
2835_p1_29	*orf29*	29976	30167	hypothetical protein	hypothetical protein [*Carnobacterium* sp. 17-4]	100	86	M	no data	no data
2835_p1_30	*parE*	30391	30966	addiction module toxin	Txe/YoeB family addiction module toxin [*Carnobacterium funditum* DSM5970]	100	97	C	ParE_toxin super family	ParE toxin of type II toxin-antitoxin system, ParDE
2835_p1_31	*parD*	31128	31394	antitoxin ParD	antitoxin ParD [*Bavariicoccus seileri*]	100	93	C	ParD super family	Antitoxin Pa
2835_p1_32	*orf32*	31379	31633	type II toxin-antitoxin system Phd/YefM family antitoxin	type II toxin-antitoxin system [*Carnobacterium alterfunditum* DSM5972]	100	95	C	XRE	transcriptional regulator, XRE-family HTH domain involved in transcription
2835_p1_33	*orf33*	31883	32080	transcriptional regulator	transcriptional regulator [*Carnobacterium alterfunditum* DSM5972]	99	86	C	XRE	transcriptional regulator, XRE-family
2835_p1_34	*orf34*	32083	32526	hypothetical protein	HTH transcriptional regulator [*Jeotgalibaca* sp. MA1X17-3]	100	99	C	XRE	transcriptional regulator, XRE-family

* Abbreviations used in protein localization prediction: C- cytoplasm; M- membrane, E- extracellular

S1a. Annotation of the 2835_p1 plasmid of *C. alterfunditum* strain 2835 (continued)

Tag	Gene	Position start	Position stop	Function	BLASTp best result	Identity (%)	Coverage (%)	Localization	Protein domain	Protein domain function
2835_p1_35	*orf35*	32603	32821	hypothetical protein	hypothetical protein [*Jeotgalibaca* sp. MA1X17-3]	100	100	E	no data	no data
2835_p1_36	*orf36*	32811	33116	hypothetical protein	hypothetical protein [*Carnobacterium pleistocenium* LM299]	94	57	E	no data	no data
2835_p1_37	*orf37*	33554	33991	hypothetical protein	hypothetical protein [*Enterococcus durans*]	100	79	E	no data	no data
2835_p1_38	*ardC*	34402	34614	peptidase	ArdC family protein peptidase [*Jeotgalibaca* sp. MA1X17-3]	97	65	C	Peptidase_M78 super family; DUF1738 family	IrrE N-terminal-like domain; Domain of unknown function
2835_p1_39	*orf39*	34626	36035	hypothetical protein	DUF3847 domain-containing protein [*Carnobacterium* sp. 17-4]	100	68	E	DUF3847 super family	Protein of unknown function (DUF3847)
2835_p1_40	*orf40*	36062	36289	hypothetical protein	hypothetical protein [*Carnobacterium* sp. 17-4]	92	69	E	no data	no data
2835_p1_41	*orf41*	36311	36643	hypothetical protein	hypothetical protein [*Carnobacterium pleistocenium* LM299]	94	57	M	no data	no data

* Abbreviations used in protein localization prediction: C- cytoplasm; M- membrane, E- extracellular

S1b. Annotation of the 2835_p2 plasmid of *C. alterfunditum* strain 2835 (complement sequence)

Tag	Gene	Position start	Position stop	Function	BLASTp best result	Identity (%)	Coverage (%)	Localization	Protein domain	Protein domain function
p2835_p2_5	*orf5*	1359	664	hypothetical protein	recombination protein [*Lactobacillus pentosus*]	90	36	C	Mob_Pre; tRNA-synt_1	tRNA synthetases class I (C) catalytic domain
p2835_p2_4	*orf4*	1751	1473	hypothetical protein	plasmid recombination protein [*Enterococcus faecium*]	98	73	C	Mob_Pre super family	Plasmid recombination enzyme
p2835_p2_3	*orf3*	2147	2893	hypothetical protein	XRE family transcriptional regulator [*Vagococcus fluvialis*]	100	44	C	HTH_XRE	Helix-turn-helix XRE-family like, DNA binding protein
p2835_p2_2	*orf2*	3722	3147	hypothetical protein	hypothetical protein [*Carnobacterium viridans* AB01]	93	93	C	HTH_MerR-like	Helix-turn-helix XRE-family like, DNA binding protein
p2835_p2_1	*repB*	4839	3756	replication protein RepB	RepB family plasmid replication initiator protein [*Carnobacterium viridans* AB01]	100	98	C	Rep_3	Initiator Replication protein

* Abbreviations used in protein localization prediction: C- cytoplasm; M- membrane, E- extracellular

S1c. Annotation of the 2835_p3 plasmid of *C. alterfunditum* strain 2835

Tag	Gene	Position		Function	BLASTp best result	Identity (%)	Coverage (%)	Localization	Protein domain	Protein domain function
		start	stop							
p2835_p3_1	*repB*	498	1667	Replication protein RepB	RepB family plasmid replication initiator protein [*Lacobacillus malefermentas* SRSPKC01]	99	99	C	Rep_3	Initiator Replication protein
p2835_p3_2	*rep*	1670	2209	replication protein	DUF536 domain-containing protein [*Vagococcus fluvialis*]	100	98	C	HTH_XRE	Helix-turn-helix XRE-family like DNA binding proteins
p2835_p3_3	*orf3*	2451	2251	hypothetical protein	hypothetical protein [*Carnobacteriaceae*]	98	84	M	no data	no data
p2835_p3_4	*orf4*	2699	2529	hypothetical protein	hypothetical protein [*C. alterfunditum* DSM5972]	100	65	C	no data	Helix-turn-helix XRE-family like DNA binding proteins
p2835_p3_5	*orf5*	2892	3404	hypothetical protein	helix-turn-helix domain-containing protein [*Carnobacterium viridans* AB01]	100	65	C	HTH_XRE	no data
p2835_p3_6	*intA*	4141	3590	prophage site-specific recombinase	integrase [*Carnobacterium funditum* DSM5970]	100	71	C	DNA_BRE_C super family	no data
p2835_p3_7	*orf7*	4356	4141	hypothetical protein	hypothetical protein [*Carnobacterium* sp. 17-4]	100	81	E	no data	Helix-turn-helix XRE-family DNA binding domain
p2835_p3_8	*lacX*	4883	4542	galactose mutarotase-like protein	galactose mutarotase-like protein [*Lactococcus lactis* subsp. *cremoris* SK11]	99	99	C	aldose-epimerase	epimerase
p2835_p3_9	*orf9*	5168	5386	hypothetical protein	hypothetical protein [*Carnobacterium alterfunditum* DSM5972]	44	95	M	no data	no data
p2835_p3_10	*orf10*	5617	5820	hypothetical protein	hypothetical protein [*Carnobacterium jeotgali* MS3]	100	64	M	no data	no data
p2835_p3_11	*orf11*	7103	6696	hypothetical protein	hypothetical protein [*Carnobacterium iners* TM377]	44	78	M	no data	no data

* Abbreviations used in protein localization prediction: C- cytoplasm; M- membrane, E- extracellular

S1d. Annotation of the 2851_p1 plasmid of *Carnobacterium* spp. strain 2851 (complement sequence)

Tag	Gene	Position start	Position stop	Function	BLASTp best result	Identity (%)	Coverage (%)	Localization	Protein domain	Protein domain function
p2851_p1_5	*galM*	2864	3082	galactose mutarotase	galactose mutarotase [*Carnobacterium maltaromaticum* LMA28]	99	98	C	Aldose_epim 1	Aldose_epim 1
p2851_p1_4	*plsY*	1032	1439	glycerol-3-phosphate O-acyltransferase	Acyl-phosphate:glycerol-3-phosphate O-acyltransferase PlsY [*Carnobacterium maltaromaticum* LMA28]	98	73	C	MFS_dom	ATP-Binding Cassette (ABC) primary transporter superfamily
p2851_p1_3	*int*	1286	1837	integrase	site-specific integrase [*Carnobacterium maltaromaticum* LMA28]	100	98	M	INT_ICEBs1_C_like	C-terminal catalytic domain of integrases
p2851_p1_2	*uvrX*	1837	2052	Putative UV-damage repair protein UvrX	Putative UV-damage repair protein UvrX [*Carnobacterium* sp. 17]	95	no data	C	UmuC	Helix-turn-helix XRE-family like DNA binding proteins belonging to the UV response element family
p2851_p1_1	*repB*	2139	3308	Replication protein Rep	RepB family plasmid replication initiator protein [*Lacobacillus malefermentas* SRSPKC01]	89	46	C	Rep_trans / Rep_trans super family	Initiator Replication protein

* Abbreviations used in protein localization prediction: C- cytoplasm; M- membrane, E- extracellular

S1e. Annotation of the 2851_p2 plasmid of *Carnobacterium* spp. strain 2851

Tag	Gene	Position		Function	BLASTp best result	Identity (%)	Coverage (%)	Localization	Protein domain	Protein domain function
		start	stop							
p2851_p2_1	*repA*	1	1002	replication protein RepA	replication initiator protein A [*Lacobacillus malefermentas* SRSPKC01]	99	57	C	HTH super family	Helix-turn-helix DNA binding domain
p2851_p2_2	*orf2*	1157	1630	hypothetical protein	hypothetical protein [*Carnobacterium* sp. 17]	99	34	E	no data	no data
p2851_p2_3	*orf3*	1968	2279	hypothetical protein	hypothetical protein [*Carnobacterium* sp. 17]	75	86	E	no data	no data
p2851_p2_4	*orf4*	2789	3016	hypothetical protein	hypothetical protein [*Carnobacterium* sp. 17]	90	34	E	no data	no data
p2851_p2_5	*orf5*	3083	7228	hypothetical protein	hypothetical protein [*Carnobacterium maltaromaticum* LMA28]	98	43	E	no data	no data
p2851_p2_6	*orf6*	7273	7434	hypothetical protein	hypothetical protein	65	55	E	no data	no data
p2851_p2_7	*orf7*	7473	7814	hypothetical protein	hypothetical protein [*Carnobacterium maltaromaticum* LMA28]	90	44	E	no data	no data
p2851_p2_8	*orf8*	7898	8071	hypothetical protein	hypothetical protein [*Carnobacterium maltaromaticum* LMA28]	82	66	M	SH3_and_anchor super family	SH3 domain protein; signal peptide
p2851_p2_9	*orf9*	8150	8536	hypothetical protein	hypothetical protein [*Carnobacterium maltaromaticum* LMA28]	99	53	M	no data	no data
p2851_p2_10	*orf10*	8548	9867	hypothetical protein	CpaF/VirB11 family protein [*Carnobacterium jeotgali* MS3]	90	71	M	VirB11-like_ATPase	type IV secretory pathway component VirB11-like
p2851_p2_11	*orf11*	9927	10772	hypothetical protein	hypothetical protein [*Carnobacterium viridans* AB01]	100	65	E	no data	no data
p2851_p2_12	*orf12*	10769	11512	hypothetical protein	hypothetical protein [*Carnobacterium jeotgali* MS3]	100	67	M	no data	no data

* Abbreviations used in protein localization prediction: C- cytoplasm; M- membrane, E- extracellular

S1e. Annotation of the 2851_p2 plasmid of *Carnobacterium* spp. strain 2851 (continued)

Tag	Gene	Position start	Position stop	Function	BLASTp best result	Identity (%)	Coverage (%)	Localization	Protein domain	Protein domain function
p2851_p2_13	*orf13*	11536	11898	hypothetical protein	DUF4320 family protein [*Carnobacterium viridans* AB01]	100	61	M	no data	no data
p2851_p2_14	*orf14*	11918	12064	hypothetical protein	hypothetical protein [*Carnobacterium viridans* AB01]	100	44	M	no data	no data
p2851_p2_15	*orf15*	12083	12355	hypothetical protein	DUF5415 family protein [*Carnobacterium viridans* AB01]	85	57	M	no data	no data
p2851_p2_16	*orf16*	12395	12667	hypothetical protein	TrbC/VirB2 family protein [*Carnobacterium maltaromaticum* LMA28]	100	57	M	TrbC super family	conjugal transfer protein
p2851_p2_17	*orf17*	12748	15138	hypothetical protein	hypothetical protein [*Carnobacterium viridans* AB01]	99	38	C	PRK12704 super family; Smc super family	phosphodiesterase
p2851_p2_18	*orf18*	15135	15749	hypothetical protein	hypothetical protein [*Carnobacterium viridans* AB01]	68	44	E	no data	no data
p2851_p2_19	*traG*	15802	18468	conjugal transfer protein	type IV secretory system conjugative DNA transfer family protein [*Carnobacterium viridans* AB01]	99	68	M	TrwB_TraG_TraD_VirD4 super family	Type IV secretory system conjugative DNA transfer protein
p2851_p2_20	*orf20*	18508	21609	hypothetical protein	hypothetical protein [*Carnobacterium viridans* AB01]	99	37	C	MDN1 super family	Midasin involved in ribosome maturation
p2851_p2_21	*orf21*	21625	21906	hypothetical protein	hypothetical protein [*Carnobacterium* sp. 17-4]	99	45	M	no data	no data
p2851_p2_22	*orf22*	21932	22561	hypothetical protein	hypothetical protein [*Peribacillus* sp. BBB004]	99	65	M	no data	no data

* Abbreviations used in protein localization prediction: C- cytoplasm; M- membrane, E- extracellular

S1e. Annotation of the 2851_p2 plasmid of *Carnobacterium* spp. strain 2851 (continued)

Tag	Gene	Position		Function	BLASTp best result	Identity	Coverage	Localization	Protein domain	Protein domain function
		start	stop			(%)	(%)			
p2851_p2_23	*virB4*	22625	24550	type IV secretory pathway VirB4 protein	VirB4 protein [*Carnobacterium jeotgali* MS3]	100	75	M	CagE_TrbE_VirB super family	component of type IV transporter system
p2851_p2_24	*nlpC*	24583	25725	NlpC/P60 family hydrolase	bifunctional lytic hydrolase/C40 family peptidase [*Peribacillus* sp. BBB004]	96	58	C	CwlT-like; NLPC_P60	CwlT-like N-terminal lysozyme domain and similar domain
p2851_p2_25	*orf25*	26726	27016	hypothetical protein	DUF5513 family protein [*Carnobacterium* sp. 17-4]	85	66	E	DUF5513 super family	Family of unknown function
p2851_p2_26	*orf26*	27003	27839	hypothetical protein	hypothetical protein [*C. alterfunditum* DSM5972]	98	43	C	no data	no data
p2851_p2_27	*orf27*	28067	28375	hypothetical protein	hypothetical protein [*Carnobacterium viridans* AB01]	100	53	M	no data	no data
p2851_p2_28	*orf28*	28390	28887	hypothetical protein	hypothetical protein [*Carnobacterium maltaromaticum* LMA28]	74	34	M	no data	no data
p2851_p2_29	*mobP*	28869	30359	relaxase	MobP2 family relaxase [*Bacillus* sp. ISL-53]	100	50	C	MobL super family	MobL relaxases
p2851_p2_30	*orf30*	30618	30863	hypothetical protein	DUF5511 family protein [*C. alterfunditum* DSM5972]	91	57	E	DUF5511 super family	Family of unknown function (DUF5511)
p2851_p2_31	*ardC*	30856	32826	conjugative protein	ArdC family protein [*Carnobacterium maltaromaticum* LMA28]	100	46	C	LPD25 super family; Peptidase_M78 super family	proteins of conjugative elements; catalytic domain of the protein ImmA
p2851_p2_32	*orf32*	32913	33968	hypothetical protein	hypothetical protein [*Carnobacterium maltaromaticum* LMA28]	37	46	M	no data	no data
p2851_p2_33	*orf33*	34025	34528	hypothetical protein	hypothetical protein hypothetical protein [*Carnobacterium maltaromaticum* LMA28]	95	34	M	no data	no data

* Abbreviations used in protein localization prediction: C- cytoplasm; M- membrane, E- extracellular

S1e. Annotation of the 2851_p2 plasmid of *Carnobacterium* spp. strain 2851 (continued)

Tag	Gene	Position start	Position stop	Function	BLASTp best result	Identity (%)	Coverage (%)	Localization	Protein domain	Protein domain function
p2851_p2_34	*lexA*	34551	35135	hydrolase	metal-dependent hydrolase [*Bacillus* sp. ISL-53]	96	56	C	YdjM super family	LexA-binding, putative hydrolase
p2851_p2_35	*orf35*	35147	36118	hypothetical protein	DUF3991 domain-containing protein [*C. alterfunditum* DSM5972]	98	54	C	TOPRIM super family; DUF3991 super family; zf-CHC2 super family	Topoisomerase-primase domain
p2851_p2_36	*orf36*	36192	36554	hypothetical protein	hypothetical protein [*Carnobacterium maltaromaticum* LMA28]	75	44	E	no data	no data
p2851_p2_37	*topB*	36616	38670	DNA topoisomerase III	type III DNA topoisomerase [*Carnobacterium viridans* AB01]	100	68	C	PRK07726 super family; Toprim_Crpt	DNA topoisomerase 3
p2851_p2_38	*orf38*	38651	39094	protease	CPBP family intramembrane metalloprotease [*C. alterfunditum* DSM5972]	91	40	C	Abi super family	CAAX protease self-immunity
p2851_p2_39	*orf39*	39291	39908	hypothetical protein	recombinase family protein [*Carnobacterium maltaromaticum* LMA28]	95	42	C	PinE super family	Site-specific DNA recombinase related to the DNA invertase Pin
p2851_p2_40	*parA*	40156	40992	ATPase involved in chromosome partitioning	ParA family protein [*C. alterfunditum* DSM5972]	96	60	C	BcsQ	Cellulose biosynthesis protein BcsQ
p2851_p2_41	*orf41*	41005	41313	hypothetical protein	hypothetical protein [*Carnobacterium divergens* DSM17]	52	37	C	Omega_Repress super family	Omega Transcriptional Repressor
p2851_p2_42	*orf42*	41338	41616	hypothetical protein	no data	no data	no data	M	no data	no data
p2851_p2_43	*orf43*	41810	42160	hypothetical protein	DUF3846 domain-containing protein [*Carnobacterium viridans* AB01]	83	35	M	no data	no data
p2851_p2_44	*orf44*	42222	42905	hypothetical protein	no data	no data	no data	M	no data	no data
p2851_p2_45	*orf45*	42930	43373	hypothetical protein	no data	no data	no data	M	no data	no data

* Abbreviations used in protein localization prediction: C- cytoplasm; M- membrane, E- extracellular

S1e. Annotation of the 2851_p2 plasmid of *Carnobacterium* spp. strain 2851 (continued)

Tag	Gene	Position		Function	BLASTp best result	Identity (%)	Coverage (%)	Localization	Protein domain	Protein domain function
		start	stop							
p2851_p2_46	*orf46*	43582	43947	hypothetical protein	hypothetical protein [*Carnobacterium* sp. 17-4]	92	48	C	no data	no data
p2851_p2_47	*tnpR*	44044	44607	recombinase	recombinase family protein [*Carnobacterium viridans* AB01]	100	95	C	SR_ResInv; HTH super family	Serine Recombinase (SR) family
p2851_p2_48	*orf48*	44688	45161	hypothetical protein	no data	no data	no data	C	no data	no data

* Abbreviations used in protein localization prediction: C- cytoplasm; M- membrane, E- extracellular

S1f. Annotation of the 2851_p3 plasmid of *Carnobacterium* spp. strain 2851 (complement sequence)

Tag	Gene	Position		Function	BLASTp best result	Identity	Coverage	Localization	Protein domain	Protein domain function
		start	stop			(%)	(%)			
p2851_p3_39	*orf39*	494	802	hypothetical protein	hypothetical protein [*Carnobacterium* sp. 17-4]	100	87	E	LCB5 super family	Diacylglycerol kinase family enzyme
p2851_p3_38	*sapC*	1228	2172	peptide transporter	peptide transporter [*Carnobacterium funditum* DSM5970]	100	88	M	PRK15111 super family	peptide ABC transporter permease SapC
p2851_p3_37	*orf37*	2300	2998	hypothetical protein	peptide transporter [*Carnobacterium funditum* DSM5970]	100	91	M	PRK15111 super family	peptide ABC transporter permease SapC
p2851_p3_36	*orf36*	3124	3309	hypothetical protein	hypothetical protein [*Carnobacterium viridans* AB01]	75	47	M	no data	no data
p2851_p3_35	*mepB*	3443	3952	*mep* protein	*mep* operon protein MepB [*Carnobacterium viridans* AB01]	100	97	C	MepB super family	MepB is a functionally uncharacterized protein
p2851_p3_34	*orf34*	4110	4298	hypothetical protein	no data	no data	no data	E	no data	no data
p2851_p3_33	*orf33*	4530	5141	hypothetical protein	stage II sporulation protein M [*Carnobacterium jeotgali* MS3]	100	77	M	DUF2441 super family	Protein of unknown function (DUF2441
p2851_p3_32	*orf32*	6730	7293	hypothetical protein	DUF2441 domain-containing protein [*Carnobacterium* sp. 17-4]	100	76	E	Vir_act_alpha_C super family	Virulence activator alpha C-term
p2851_p3_31	*nemA*	7437	8531	NADH-dependent flavin oxidoreductase	NADH-dependent flavin oxidoreductase [*Carnobacterium divergens* DSM17]	99	66	C	OYE_like_4_FMN	Old yellow enzyme (OYE)-related FMN binding domain
p2851_p3_30	*umuD*	8990	9883	DNA polymerase	Y-family DNA polymerase [*Carnobacterium maltaromaticum* LMA28]	100	87	C	PolY_Pol_V_umuC	umuC subunit of DNA Polymerase V
p2851_p3_29	*orf29*	9876	10475	hypothetical protein	DUF1700 domain-containing protein [*Carnobacterium divergens* DSM17]	95	37	M	DUF1700 super family	Protein of unknown function (DUF1700)
p2851_p3_28	*padR*	10459	10785	PadR family transcriptional regulator	PadR family transcriptional regulator [*Carnobacterium divergens* DSM17]	99	65	C	Vir_act_alpha_C super family	Virulence activator alpha C-term

* Abbreviations used in protein localization prediction: C- cytoplasm; M- membrane, E- extracellular

S1f. Annotation of the 2851_p3 plasmid of *Carnobacterium* spp. strain 2851 (continued)

Tag	Gene	Position start	Position stop	Function	BLASTp best result	Identity (%)	Coverage (%)	Localization	Protein domain	Protein domain function
p2851_p3_27	*parA*	12720	13538	ATPase involved in chromosome partitioning	ParA family protein [*Carnobacterium* sp. 17-4]	100	94	C	BcsQ	Cellulose biosynthesis protein BcsQ, involed in cell motility
p2851_p3_26	*orf26*	13569	13988	hypothetical protein	hypothetical protein [*Carnobacterium* sp. 17-4]	100	93	M	no data	no data
p2851_p3_25	*orf25*	14106	14783	hypothetical protein	Fic family protein [*Carnobacterium* sp. 17-4]	100	85	C	COG3177 super family	Fic family protein, involved in transcription
p2851_p3_24	*tnpR*	15118	15714	recombinase	recombinase family protein [*Carnobacterium inhibens* WN1359]	98	94	C	SR_ResInv	Serine Recombinase (SR) family, Resolvase and Invertase subfamily
p2851_p3_23	*kdpD*	16227	18899	sensor histidine kinase KdpD	sensor histidine kinase KdpD [*Carnobacterium iners* TM377]	100	85	M	KdpD	K+-sensing histidine kinase KdpD, signal transduction mechanisms
p2851_p3_22	*kdpE*	18892	19602	KDP operon transcriptional regulatory protein	response regulator transcription factor [*Carnobacterium iners* TM377]	100	92	C	KdpE	DNA-binding response regulator
p2851_p3_21	*kdpA*	19986	21704	potassium-transporting ATPase A chain	potassium-transporting ATPase subunit KdpA [*Carnobacterium iners* TM377]	100	88	M	KdpA super family	Potassium-transporting ATPase A subunit, KdpA
p2851_p3_20	*kdpB*	21718	23772	potassium-transporting ATPase B chain	potassium-transporting ATPase subunit KdpB [*Carnobacterium iners* TM377]	100	93	M	P-type_ATPase_K	Potassium-transporting ATPase ATP-binding subunit, KdpB
p2851_p3_19	*kdpC*	23847	24479	potassium-transporting ATPase C chain	K(+)-transporting ATPase subunit C [*Carnobacterium iners* TM377]	98	80	M	KdpC super family	K+-transporting ATPase, c chain
p2851_p3_18	*orf18*	24783	25319	hypothetical protein	DNA/RNA non-specific endonuclease [*Carnobacterium divergens* DSM17]	98	78	M	no data	no data
p2851_p3_17	*pdaC*	25490	26947	peptidoglycan-N-acetylmuramic acid deacetylase PdaC	polysaccharide deacetylase family protein [*Carnobacterium* sp. 17-4]	99	61	C	CE4_SF super family, DUF3298	Catalytic NodB homology domain of the carbohydrate esterase 4 superfamily

* Abbreviations used in protein localization prediction: C- cytoplasm; M- membrane, E- extracellular

S1f. Annotation of the 2851_p3 plasmid of *Carnobacterium* spp. strain 2851 (continued)

Tag	Gene	Position start	Position stop	Function	BLASTp best result	Identity (%)	Coverage (%)	Localization	Protein domain	Protein domain function
p2851_p3_16	*orf16*	27214	27510	hypothetical protein	hypothetical protein [*Carnobacterium iners* TM377]	62	75	M	no data	no data
p2851_p3_15	*orf15*	27477	27725	hypothetical protein	transposase [*Carnobacterium* sp. 17-4]	100	77	C	transpos_ISL3 super family	ISL3 family transposase
p2851_p3_14	*orf14*	28023	28349	hypothetical protein	ISL3 family transposase [*Carnobacterium* sp. 17-4]	74	89	C	transpos_ISL3 super family	ISL3 family transposase
p2851_p3_13	*mobA*	28265	30241	mobilisation protein	MobA/MobL family protein [*Carnobacterium* sp. 17-4]	100	73	C	MobA_MobL; NES_C_h super family	MobA/MobL family; Nicking enzyme C-terminal middle helical domain
p2851_p3_12	*orf12*	30459	30791	hypothetical protein	hypothetical protein [*Carnobacterium* sp. 17-4]	100	92	M	no data	no data
p2851_p3_11	*orf11*	30813	31040	hypothetical protein	DUF3847 domain-containing protein [*Carnobacterium* sp. 17-4]	100	68	M	DUF3847 super family	Protein of unknown function (DUF3847); Domain of unknown function (DUF1738)
p2851_p3_10	*orf10*	31067	32476	hypothetical protein	ArdC family protein [*Jeotgalibaca* sp. MA1X17-3]	97	64	C	Peptidase_M78 super family; DUF1738 super family	IrrE N-terminal-like domain
p2851_p3_9	*orf9*	32488	32700	hypothetical protein	hypothetical protein [*Carnobacterium divergens* DSM17]	100	78	M	no data	no data
p2851_p3_8	*orf8*	33111	33548	hypothetical protein	hypothetical protein [*Carnobacterium pleistocenium* AT288]	94	56	M	no data	no data
p2851_p3_7	*orf7*	33986	34291	hypothetical protein	hypothetical protein [*Jeotgalibaca* sp. MA1X17-3]	100	100	M	no data	no data

* Abbreviations used in protein localization prediction: C- cytoplasm; M- membrane, E- extracellular

S1f. Annotation of the 2851_p3 plasmid of *Carnobacterium* spp. strain 2851 (continued)

Tag	Gene	Position start	Position stop	Function	BLASTp best result	Identity (%)	Coverage (%)	Localization	Protein domain	Protein domain function
p2851_p3_6	*orf6*	34281	34499	hypothetical protein	hypothetical protein [*Jeotgalibaca* sp. MA1X17-3]	100	100	M	no data	no data
p2851_p3_5	*orf5*	34576	35019	hypothetical protein	hypothetical protein [*Carnobacterium alterfunditum* DSM5972]	99	86	M	no data	no data
p2851_p3_4	*xre*	35022	35219	XRE family transcriptional regulator	helix-turn-helix transcriptional regulator [*Carnobacterium alterfunditum* DSM5972]	100	95	C	XRE	DNA-binding transcriptional regulator, XRE-family HTH domain
p2851_p3_3	*yefM*	35469	35723	antitoxin	type II toxin-antitoxin system Phd/YefM family antitoxin [*Carnobacterium divergens* DSM17]	100	93	C	StbD super family	Antitoxin component YafN of the YafNO toxin-antitoxin module
p2851_p3_2	*parE*	35708	35974	Txe/YoeB family addiction module toxin	Txe/YoeB family addiction module toxin [*Carnobacterium funditum* DSM5970]	100	96	C	ParE_toxin super family	ParE toxin of type II toxin-antitoxin system, parDE
p2851_p3_1	*repB*	36136	37126	replication protein	replication protein B [*Carnobacterium maltaromaticum* LMA28]	100	97	C	Rep_3	Initiator Replication protein

* Abbreviations used in protein localization prediction: C- cytoplasm; M- membrane, E- extracellular

S1g. Annotation of the 2856_p1 plasmid of *Carnobacterium* spp. strain 2856

Tag	Gene	Position		Function	BLASTp best result	Identity (%)	Coverage (%)	Localization	Protein domain	Protein domain function
		start	stop							
p2856_p1_1	*orf28*	1141	1449	hypothetical protein	hypothetical protein/ nickase [*Carnobacterium maltaromaticum* LMA28]	100/100	66	C	MobA_MobL	MobA/MobL family; mobilisation proteins
p2856_p1_2	*orf27*	1470	1697	hypothetical protein	hypothetical protein [*Carnobacterium maltaromaticum* LMA28]	90	52	E	no data	no data
p2856_p1_3	*orf26*	1720	3123	hypothetical protein	hypothetical protein [*Carnobacterium iners* TM377]	98	64	M	DUF3847 super family	Protein of unknown function (DUF3847)
p2856_p1_4	*orf25*	3123	3335	hypothetical protein	ImmA/IrrE family metallo-endopeptidase [*Carnobacterium iners* TM377]	100	58	C	DUF955 super family	Domain of unknown function (DUF955)
p2856_p1_5	*orf24*	3599	4666	hypothetical protein	Uncharacterised protein [*Carnobacterium maltaromaticum* LMA28]	95	48	E	no data	no data
p2856_p1_6	*orf23*	4841	4999	hypothetical protein	Transcriptional regulator, IclR family [*Carnobacterium iners* TM377]	93	38	C	HTH super family	DNA binding domain
p2856_p1_7	*orf22*	5085	5279	hypothetical protein	no data	no data	no data		no data	no data
p2856_p1_8	*parB*	5282	6061	hypothetical protein	ParB family protein [*Carnobacterium iners* TM377]	98	54	C	HTH_XRE	DNA binding domain
p2856_p1_9	*parA*	6157	6714	partition protein A	ParA family protein [*Carnobacterium maltaromaticum* LMA28]	98	58	C	HTH_XRE	DNA binding domain
p2856_p1_10	*orf19*	6868	7089	hypothetical protein	recombinase family protein [*Carnobacterium iners* TM377]	97	69	C	SR_ResInv / HTH super family	DNA binding domain
p2856_p1_11	*orf18*	7471	7791	hypothetical protein	no data	no data	no data	C	HAD_like super family	Haloacid Dehalogenase-like Hydrolases
p2856_p1_12	*orf17*	7809	8069	hypothetical protein	no data	no data	no data	E	no data	no data
p2856_p1_13	*orf16*	8140	8451	hypothetical protein	hypothetical protein [*Carnobacterium iners* TM377]	80	39	M	no data	no data

* Abbreviations used in protein localization prediction: C- cytoplasm; M- membrane, E- extracellular

S1g. Annotation of the 2856_p1 plasmid of *Carnobacterium* spp. strain 2856 (continued)

Tag	Gene	Position start	Position stop	Function	BLASTp best result	Identity (%)	Coverage (%)	Localization	Protein domain	Protein domain function
p2856_p1_14	*orf15*	8444	8743	hypothetical protein	hypothetical protein [*Carnobacterium iners* TM377]	86	57	M	no data	no data
p2856_p1_15	*topB*	9257	11371	DNA topoisomerase III	type IA DNA topoisomerase [*Carnobacterium maltaromaticum* LMA28]	100	70	C	TOP1Ac super family	DNA Topoisomerase, subtype IA
p2856_p1_16	*orf13*	11386	11886	hypothetical protein	no data	no data	no data	M	TOP1Ac super family	DNA Topoisomerase, subtype IA
p2856_p1_17	*topA*	11910	12737	DNA topoisomerase III	type IA DNA topoisomerase [*Carnobacterium* sp. 17-4]	100	70	C	TOP1Ac super family	DNA Topoisomerase, subtype IA
p2856_p1_18	*orf11*	12754	14316	hypothetical protein	peptidoglycan-binding protein [*Carnobacterium* sp. 17-4]	92	27	E	no data	no data
p2856_p1_19	*orf10*	14316	14756	hypothetical protein	hypothetical protein [*Carnobacterium maltaromaticum* LMA28]	99	70	M	no data	no data
p2856_p1_20	*trwB*	14773	15321	conjugation protein	conjugal transfer protein [*Carnobacterium iners* TM377]	99	80	C	TrwB_AAD_bind super famil	Type IV secretion-system coupling protein DNA-binding domain
p2856_p1_21	*orf8*	15339	16331	hypothetical protein	hypothetical protein [*Carnobacterium iners* TM377]	97	42	M	no data	no data
p2856_p1_22	*orf7*	16345	17715	hypothetical protein	hypothetical protein [*Carnobacterium iners* TM377]	99	63	M	no data	no data
p2856_p1_23	*yddH*	17730	19691	endopeptidase	endopeptidase YddH [*Carnobacterium maltaromaticum* LMA28]	85	77	C	Lysosyme / NLPC P60	Lysozym
p2856_p1_24	*yukC*	19709	20368	conjugal transfer protein	conjugal transfer protein [*Carnobacterium iners* TM377]	99	80	M	YukC super family / SMC_N super family	protein secretion system (Wss)
p2856_p1_25	*orf4*	20392	20736	hypothetical protein	DUF87 domain-containing protein [*Carnobacterium iners* TM377]	100	83	E	VirB4 super family	Type IV secretory pathway, VirB4 component

* Abbreviations used in protein localization prediction: C- cytoplasm; M- membrane, E- extracellular

S1g. Annotation of the 2856_p1 plasmid of *Carnobacterium* spp. strain 2856 (continued)

Tag	Gene	Position start	Position stop	Function	BLASTp best result	Identity (%)	Coverage (%)	Localization	Protein domain	Protein domain function
p2856_p1_26	*orf3*	20760	21095	hypothetical protein	conjugal transfer protein [*Carnobacterium maltaromaticum* LMA28]	94	72	E	no data	no data
p2856_p1_27	*mobA*	21098	21655	mobilisation protein	conjugal transfer protein [*Carnobacterium maltaromaticum* LMA28]	95	45	C	MobA_MobL; NES_C_h super family	MobA/MobL family; Nicking enzyme C-terminal middle helical domain
p2856_p1_28	*repB*	21790	22404	replication protein RepB	replication protein [*Carnobacterium maltaromaticum* LMA28]	100	81	C	Rep_3	Initiator replication protein

* Abbreviations used in protein localization prediction: C- cytoplasm; M- membrane, E- extracellular

S1h. Annotation of the 2859_p1 plasmid of *Carnobacterium* spp. strain 2859 (complement sequence)

Tag	Gene	Position start	Position stop	Function	BLASTp best result	Identity (%)	Coverage (%)	Localization	Protein domain	Protein domain function
p2859_p1_44	*parA*	1631	2449	ATPase involved in chromosome partitioning	ParA family protein [*Carnobacterium* sp. 17-4]	100	95	C	HTH_XRE	Helix-turn-helix XRE-family DNA binding domain
p2859_p1_43	*orf43*	2480	2899	hypothetical protein	hypothetical protein [*Carnobacterium* sp. 17-4]	100	88	E	no data	no data
p2859_p1_42	*orf42*	3177	3377	hypothetical protein	Fic family protein [*Carnobacterium* sp. 17-4]	100	74	E	no data	no data
p2859_p1_41	*orf41*	3381	3707	hypothetical protein	Fic family protein [*Carnobacterium* sp. 17-4]	100	86	M	COG3177 super family	Fic family protein involved in transcription
p2859_p1_40	*kduD*	3856	4701	gluconate 5-dehydrogenase	dehydrogenase [*Jeotgalibaca* sp. MA1X17-3]	100	94	C	PRK08277	D-mannonate oxidoreductase
p2859_p1_39	*uxuA*	4733	5773	mannonate dehydratase	mannonate dehydratase [*Jeotgalibaca* sp. MA1X17-3]	100	91	C	AP2Ec super family	AP endonuclease family 2 with role in DNA repair
p2859_p1_38	*uxaC*	5809	7221	glucuronate isomerase	glucuronate isomerase [*Carnobacterium* sp. AT7]	70	83	C	UxaC super family	Glucuronate isomerase
p2859_p1_37	*kdgA*	7267	7917	2-dehydro-3-deoxyphosphogluconate aldolase	2-dehydro-3-deoxyphosphogluconate aldolase [*Jeotgalibaca* sp. MA1X17-3]	100	85	C	TIM super family	TIM-like beta/alpha barrel domains
p2859_p1_36	*kdgK*	8251	9201	2-dehydro-3-deoxygluconokinase	sugar kinase [*Jeotgalibaca* sp. MA1X17-3]	99	86	C	KdgK	2-keto-3-deoxygluconate kinase (KdgK)
p2859_p1_35	*orf35*	9275	10315	hypothetical protein	TRAP transporter substrate-binding protein [*Jeotgalibaca* sp. MA1X17-3]	100	92	M	PBP2_TRAP_SBP_like_3	ATP-independent Periplasmic transporter family
p2859_p1_34	*dctR*	10315	10785	TRAP transporter	TRAP transporter substrate-binding protein [*Jeotgalibaca* sp. MA1X17-3]	87	86	C	DctM super family	TRAP-type C4-dicarboxylate transport system, small permease component

* Abbreviations used in protein localization prediction: C- cytoplasm; M- membrane, E- extracellular

S1h. Annotation of the 2859_p1 plasmid of *Carnobacterium* spp. strain 2859 (complement sequence, continued)

Tag	Gene	Position start	Position stop	Function	BLASTp best result	Identity (%)	Coverage (%)	Localization	Protein domain	Protein domain function
p2859_p1_33	*dctQ*	10819	12123	TRAP transporter	TRAP transporter large permease [*Jeotgalibaca* sp. MA1X17-3]	100	96	C	DctQ super family	TRAP-type C4-dicarboxylate transport system, large permease component
p2859_p1_32	*iclR*	12376	13143	IclR family transcriptional regulator	IclR family transcriptional regulator [*Jeotgalibaca* sp. MA1X17-3]	100	88	C	IclR	DNA-binding transcriptional regulator, IclR family
p2859_p1_31	*orf31*	13276	13446	hypothetical protein	no data	no data	no data	M	no data	no data
p2859_p1_30	*tnpR*	13549	14121	recombinase	recombinase family protein [*Jeotgalibaca* sp. MA1X17-3]	96	95	C	SR_ResInv	Serine Recombinase (SR) family
p2859_p1_29	*orf29*	14365	15249	hypothetical protein	IS1380 family transposase [*Carnobacterium iners* TM377]	100	91	C	DDE_Tnp_1_4 super family	Transposase DDE domain group 1
p2859_p1_28	*orf28*	15394	15675	hypothetical protein	IS1380 family transposase [*Carnobacterium maltaromaticum* LMA28]	100	86	C	DDE_Tnp_1_4 super family	Transposase DDE domain group 1
p2859_p1_27	*orf27*	16052	16642	hypothetical protein	transposase [*Carnobacterium maltaromaticum* LMA28]	100	93	C	transpos_IS30 super family	IS30 family transposase
p2859_p1_26	*orf26*	16715	17011	hypothetical protein	IS30 family transposase [*Carnobacterium alterfunditum* DSM5972]	100	97	C	Tra8 super family	Transposase and inactivated derivatives
p2859_p1_25	*kyeA*	17195	19144	2-enoate reductase	FAD-dependent oxidoreductase [*Jeotgalibaca* sp. MA1X17-3]	99	75	C	Pyr_redox_2	Pyridine nucleotide-disulphide oxidoreductase
p2859_p1_24	*orf24*	19303	20187	hypothetical protein	helix-turn-helix domain-containing protein [*Jeotgalibaca* sp. MA1X17-3]	97	62	C	AraC	AraC-type DNA-binding domain
p2859_p1_23	*orf23*	20333	20653	hypothetical protein	DNA topoisomerase [*Carnobacterium* sp. 17-4]	99	71	C	PRK07726 super family	DNA topoisomerase 3
p2859_p1_22	*mobA*	20747	22738	mobilisation protein	MobA/MobL family protein [*Carnobacterium* sp. 17-4]	100	78	C	MobA_MobL	MobA/MobL family

* Abbreviations used in protein localization prediction: C- cytoplasm; M- membrane, E- extracellular

S1h. Annotation of the 2859_p1 plasmid of *Carnobacterium* spp. strain 2859 (complement sequence, continued)

Tag	Gene	Position start	Position stop	Function	BLASTp best result	Identity (%)	Coverage (%)	Localization	Protein domain	Protein domain function
p2859_p1_21	*orf21*	22946	23254	hypothetical protein	hypothetical protein [*Carnobacterium* sp. 17-4]	95	62	E	no data	no data
p2859_p1_20	*orf20*	23274	23501	hypothetical protein	hypothetical protein [*Carnobacterium* sp. 17-4]	100	72	E	no data	no data
p2859_p1_19	*orf19*	23528	24010	hypothetical protein	hypothetical protein [*Carnobacterium* sp. 17-4]	100	63	E	no data	no data
p2859_p1_18	*orf18*	24046	24432	hypothetical protein	ArdC-like ssDNA-binding domain-containing protein [*Carnobacterium divergens* LV13]	82	72	M	Peptidase_M78 super family	IrrE N-terminal-like domain
p2859_p1_17	*orf17*	24720	24923	hypothetical protein	hypothetical protein [*Jeotgalibaca* sp. MA1X17-3]	100	82	M	no data	no data
p2859_p1_16	*orf16*	25469	25654	hypothetical protein	hypothetical protein [*Carnobacterium inhibens* WN1359]	100	93	M	no data	no data
p2859_p1_15	*orf15*	25647	26132	hypothetical protein	thioredoxin family protein [*Carnobacterium inhibens* WN1359]	100	97	C	Thioredoxin_like super family	Protein Disulfide Oxidoreductases
p2859_p1_14	*orf14*	26119	26652	hypothetical protein	hypothetical protein [*Carnobacterium inhibens* WN1359]	100	94	M	no data	no data
p2859_p1_13	*orf13*	26816	27241	hypothetical protein	helix-turn-helix transcriptional regulator [*Carnobacterium inhibens* WN1359]	100	99	C	HTH_XRE	DNA binding domain
p2859_p1_12	*orf12*	27447	29171	hypothetical protein	ATP-binding protein [*Carnobacterium divergens* LV13]	99	45	C	AAA_21 super family	AAA domain, putative AbiEii toxin, Type IV TA system
p2859_p1_11	*hipB*	29494	30249	transcription regulator	Rgg/GadR/MutR family transcriptional regulator [*Carnobacterium pleistocenium* FTR1]	99	78	C	RGG_Cterm super family	transcriptional activator

* Abbreviations used in protein localization prediction: C- cytoplasm; M- membrane, E- extracellular

S1h. Annotation of the 2859_p1 plasmid of *Carnobacterium* spp. strain 2859 (complement sequence, continued)

Tag	Gene	Position start	Position stop	Function	BLASTp best result	Identity (%)	Coverage (%)	Localization	Protein domain	Protein domain function
p2859_p1_10	*orf10*	30559	30975	hypothetical protein	hypothetical protein [*Carnobacterium mobile* S2]	99	73	M	no data	no data
p2859_p1_9	*orf9*	31032	31634	hypothetical protein	hypothetical protein [*Carnobacterium mobile* S2]	100	63	M	no data	no data
p2859_p1_8	*orf8*	31758	32180	hypothetical protein	hypothetical protein [*Carnobacterium inhibens* WN1359]	100	80	M	no data	no data
p2859_p1_7	*xre1*	32177	32386	XRE family transcriptional regulator	helix-turn-helix transcriptional regulator [*Carnobacteriaceae*]	100	95	C	XRE	DNA-binding transcriptional regulator
p2859_p1_6	*xre2*	32568	32765	XRE family transcriptional regulator	helix-turn-helix transcriptional regulator [*Carnobacteriaceae*]	100	93	C	XRE	DNA-binding transcriptional regulator
p2859_p1_5	*orf5*	32772	33188	hypothetical protein	membrane protein [*Carnobacterium inhibens* WN1359]	100	85	M	no data	no data
p2859_p1_4	*orf4*	33325	34110	hypothetical protein	NADH-dependent flavin oxidoreductase [*Trichococcus patagoniensis*]	99	66	M	TIM super family	TIM-like beta/alpha barrel domains
p2859_p1_3	*orf3*	34227	34418	hypothetical protein	NADH-dependent flavin oxidoreductase [*Carnobacterium maltaromaticum* LMA28]	98	69	M	TIM super family	TIM-like beta/alpha barrel domains
p2859_p1_2	*orf2*	34857	35750	hypothetical protein	DUF4097 family beta strand repeat protein [*Carnobacterium* sp. 17-4]	78	35	M	DUF4097 super family	Putative adhesin
p2859_p1_1	*repB*	35743	36820	replication protein	replication protein RepB [*Carnobacterium* sp. 17-4]	34	59	C	Rep_3	Initiator Replication protein

* Abbreviations used in protein localization prediction: C- cytoplasm; M- membrane, E- extracellular

S1i. Annotation of the 2862_p1 plasmid of *C. maltaromaticum* strain 2862

Tag	Gene	Position start	Position stop	Function	BLASTp best result	Identity (%)	Coverage (%)	Localization	Protein domain	Protein domain function
p2862_p1_1	*repB*	797	1735	replication protein B	Initiator replication protein [*Carnobacterium iners* TM377]	97	58	C	Rep_3	Initiator Replication protein
p2862_p1_2	*rep*	3692	4402	replication initiation protein	Ireplication protein A [*Carnobacterium iners* TM377]	96	57	C	HTH_XRE	Helix-turn-helix XRE-family like DNA binding domain
p2862_p1_3	*orf3*	4608	4952	hypothetical protein	MobV family relaxase [*Bacillus* sp. ISL-7]	82	37	C	MobM_relaxase super family	relaxase domain of MobM and similar proteins
p2862_p1_4	*mob*	5183	6493	Mob protein	mobilization protein [*Carnobacterium maltaromaticum* LMA28]	98	87	C	HTH_XRE	Helix-turn-helix XRE-family DNA binding domain
p2862_p1_5	*pepA*	8400	9296	Neutral endopeptidase	endopeptidase pepA [*Carnobacterium inhibens* WN1359]	80	44	C	Peptidase_M78	peptidase
p2862_p1_6	*cspD*	9802	10071	cold shock protein D	cold shock protein [*Carnobacterium inhibens* WN1359]	100	90	M	CspA	Cold shock protein, CspA family
p2862_p1_7	*orf7*	10185	10358	hypothetical protein	hypothetical protein [*Carnobacterium inhibens* WN1359]	87	58	M	no data	no data

* Abbreviations used in protein localization prediction: C- cytoplasm; M- membrane, E- extracellular

S1j. Annotation of the 2862_p2 plasmid of *C. maltaromaticum* strain 2862 (complement sequence)

Tag	Gene	Position		Function	BLASTp best result	Identity (%)	Coverage (%)	Localization	Protein domain	Protein domain function
		start	stop							
p2862_p2_73	*orf73*	1	529	hypothetical protein	hypothetical protein [*Carnobacterium maltaromaticum* LMA28]	91	92	M	no data	no data
p2862_p2_72	*orf72*	830	1492	transposase	transposase [*Carnobacterium maltaromaticum* LMA28]	100	93	C	transpos_IS3	IS3 family transposase
p2862_p2_71	*orf71*	1648	1914	transposase	transposase [*Carnobacterium maltaromaticum* LMA28]	94	95	C	HTH super family	DNA binding domain
p2862_p2_70	*orf70*	1929	2786	hypothetical protein	no data	no data	no data	M	no data	no data
p2862_p2_69	*orf69*	2913	3341	hypothetical protein	helix-turn-helix domain-containing protein [*Carnobacterium inhibens* WN1359]	90	42	C	CxxCG_CxxCG_HTH super family	putative zinc finger/helix-turn-helix protein
p2862_p2_68	*orf68*	3296	3943	hypothetical protein	hypothetical protein [*Carnobacterium inhibens* WN1359]	89	42	M	no data	no data
p2862_p2_67	*orf67*	3943	4317	hypothetical protein	N-acetylmuramoyl-L-alanine amidase [*Carnobacterium maltaromaticum* LMA28]	99	54	C	PGRP super family	Peptidoglycan recognition proteins (PGRPs)
p2862_p2_66	*orf66*	4512	5468	hypothetical protein	holin [*Carnobacterium inhibens* WN1359]	95	67	C	Phage_holin_5_2 super family	Phage holin family Hol44, in holin superfamily V
p2862_p2_65	*orf65*	5468	5734	hypothetical protein	hypothetical protein [*Carnobacterium maltaromaticum* LMA28]	100	88	M	no data	no data
p2862_p2_64	*orf64*	5751	6032	hypothetical protein	hypothetical protein [*Carnobacterium maltaromaticum* LMA28]	57	47	M	no data	no data

* Abbreviations used in protein localization prediction: C- cytoplasm; M- membrane, E- extracellular

S1j. Annotation of the 2862_p2 plasmid of *C. maltaromaticum* strain 2862 (complement sequence, continued)

Tag	Gene	Position		Function	BLASTp best result	Identity	Coverage	Localization	Protein domain	Protein domain function
		start	stop			(%)	(%)			
p2862_p2_63	*orf63*	6029	6376	hypothetical protein	glycerophosphodiester phosphodiesterase family protein [*Carnobacterium maltaromaticum* LMA28]	72	74	M	no data	no data
p2862_p2_62	*orf62*	6388	7869	hypothetical protein	phage tail spike protein [*Carnobacterium maltaromaticum* LMA28]	100	92	C	Prophage_tail super family	Prophage endopeptidase tail
p2862_p2_61	*orf61*	7883	9538	hypothetical protein	hypothetical protein [*Carnobacterium maltaromaticum* LMA28]	100	97	M	no data	no data
p2862_p2_60	*orf60*	9538	10248	hypothetical protein	phage tail tape measure protein [*Carnobacterium maltaromaticum* LMA28]	100	94	C	PhageMin_Tail super family	Phage-related minor tail protein
p2862_p2_59	*orf59*	10245	15140	phage tail tape measure protein	phage protein [*Carnobacterium maltaromaticum* LMA28]	100	100	C	PhageMin_Tail super family	Phage-related minor tail protein
p2862_p2_58	*orf58*	15170	15337	hypothetical protein	phage major tail, phi13 family protein [*Carnobacterium inhibens* WN1359]	100	75	M	no data	no data
p2862_p2_57	*orf57*	15367	15684	hypothetical protein	hypothetical protein [*Carnobacterium maltaromaticum* LMA28]	100	100	M	Phage_TTP_1 super family	Phage tail tube protein
p2862_p2_56	*orf56*	15786	16379	hypothetical protein	hypothetical protein [*Carnobacterium maltaromaticum* LMA28]	100	99	M	no data	no data
p2862_p2_55	*orf55*	16406	16795	hypothetical protein	HK97 gp10 family phage protein [*Carnobacterium maltaromaticum* LMA28]	100	97	C	HK97-gp10_like super family	Bacteriophage HK97-gp10, putative tail-component
p2862_p2_54	*orf54*	16792	17190	hypothetical protein	phage head closure protein [*Carnobacterium maltaromaticum* LMA28]	100	99	M	Phage_H_T_join super family	Phage head-tail joining protein

* Abbreviations used in protein localization prediction: C- cytoplasm; M- membrane, E- extracellular

S1j. Annotation of the 2862_p2 plasmid of *C. maltaromaticum* strain 2862 (complement sequence, continued)

Tag	Gene	Position start	Position stop	Function	BLASTp best result	Identity (%)	Coverage (%)	Localization	Protein domain	Protein domain function
p2862_p2_53	*orf53*	17168	17533	hypothetical protein	hypothetical protein [*Carnobacterium maltaromaticum* LMA28]	100	99	M	no data	no data
p2862_p2_52	*orf52*	17517	17813	hypothetical protein	hypothetical protein [*Carnobacterium maltaromaticum* LMA28]	100	95	M	no data	no data
p2862_p2_51	*orf51*	17830	18111	hypothetical protein	hypothetical protein [*Carnobacterium maltaromaticum* LMA28]	99	98	M	no data	no data
p2862_p2_50	*orf50*	18180	19364	phage capsid protein	phage major capsid protein [*Carnobacterium maltaromaticum* LMA28]	100	99	M	major_cap_HK97 super family	phage major capsid protein
p2862_p2_49	*clp*	19381	20091	ATP-dependent Clp protease	Clp protease ClpP [*Carnobacterium maltaromaticum* LMA28]	100	98	C	S14_ClpP_1	Caseinolytic protease (ClpP)
p2862_p2_48	*orf48*	20078	21355	phage portal protein	phage portal protein [*Carnobacterium maltaromaticum* LMA28]	100	99	C	Phage_portal super family	Phage portal protein
p2862_p2_47	*orf47*	21511	21687	hypothetical protein	hypothetical protein [*Carnobacterium maltaromaticum* LMA28]	99	98	M	no data	no data
p2862_p2_46	*ymfN*	21690	22007	YmfN terminase	terminase large subunit [*Carnobacterium maltaromaticum* LMA28]	100	99	M	YmfN super family	Phage terminase-like protein
p2862_p2_45	*xtmB*	22020	23741	phage terminase large subunit	phage terminase small subunit P27 family [*Carnobacterium maltaromaticum* LMA28]	100	100	C	Terminase_4 super family	Phage terminase, small subunit
p2862_p2_44	*orf44*	23738	24214	hypothetical protein	putative phage-related protein [*Carnobacterium maltaromaticum* LMA28]	100	99	C	HNHc super family	HNH nucleases

* Abbreviations used in protein localization prediction: C- cytoplasm; M- membrane, E- extracellular

S1j. Annotation of the 2862_p2 plasmid of *C. maltaromaticum* strain 2862 (complement sequence, continued)

Tag	Gene	Position		Function	BLASTp best result	Identity	Coverage	Localization	Protein domain	Protein domain function
		start	stop			(%)	(%)			
p2862_p2_43	*orf43*	24455	24799	hypothetical protein	hypothetical protein [*Carnobacterium maltaromaticum* LMA28]	100	94	E	no data	no data
p2862_p2_42	*orf42*	24792	25136	hypothetical protein	hypothetical protein [*Carnobacterium maltaromaticum* LMA28]	100	97	E	no data	no data
p2862_p2_41	*orf41*	25143	25406	hypothetical protein	hypothetical protein [*Carnobacterium maltaromaticum* LMA28]	57	47	M	no data	no data
p2862_p2_40	*orf40*	25981	26056	hypothetical protein	hypothetical protein [*Carnobacterium maltaromaticum* LMA28]	96	88	M	no data	no data
p2862_p2_39	*arpU*	26684	27115	autolysin	phage transcriptional regulator, ArpU family protein [*Carnobacterium maltaromaticum* LMA28]	100	98	M	phage_arpU super family	phage transcriptional regulator, ArpU family
p2862_p2_38	*orf38*	27128	27739	hypothetical protein	DNA polymerase III subunit beta [*Enterococcus faecalis* LM2876]	99	49	M	beta_clamp super family	The beta subunit (processivity factor) of DNA polymerase III holoenzyme
p2862_p2_37	*orf37*	27752	27880	hypothetical protein	hypothetical protein [*Carnobacterium maltaromaticum* LMA28]	100	100	M	no data	no data
p2862_p2_36	*orf36*	27877	28029	hypothetical protein	hypothetical protein [*Carnobacterium maltaromaticum* LMA28]	100	94	M	no data	no data
p2862_p2_35	*ssb*	28045	28491	single-stranded DNA-binding protein	single-stranded DNA-binding protein [*Carnobacterium maltaromaticum* LMA28]	100	93	C	RPA_2b-aaRSs_OBF_like super family	Replication protein A

* Abbreviations used in protein localization prediction: C- cytoplasm; M- membrane, E- extracellular

S1j. Annotation of the 2862_p2 plasmid of *C. maltaromaticum* strain 2862 (complement sequence, continued)

Tag	Gene	Position		Function	BLASTp best result	Identity (%)	Coverage (%)	Localization	Protein domain	Protein domain function
		start	stop							
p2862_p2_34	*orf34*	28495	28812	hypothetical protein	hypothetical protein [*Carnobacterium maltaromaticum* LMA28]	96	88	M	no data	no data
p2862_p2_33	*orf33*	28812	29036	hypothetical protein	YopX family protein [*Carnobacterium maltaromaticum* LMA28]	100	75	M	YopX super family	YopX protein
p2862_p2_32	*orf32*	29033	29455	hypothetical protein	hypothetical protein [*Carnobacterium maltaromaticum* LMA28]	100	98	M	no data	no data
p2862_p2_31	*orf31*	29461	29703	hypothetical protein	conserved hypothetical protein [*Carnobacterium maltaromaticum* LMA28]	99	84	M	DUF1064 super family	Protein of unknown function (DUF1064)
p2862_p2_30	*orf30*	29748	30158	hypothetical protein	hypothetical protein [*Carnobacterium maltaromaticum* LMA28]	98	98	M	no data	no data
p2862_p2_29	*orf29*	30148	30339	hypothetical protein	hypothetical protein [*Carnobacterium maltaromaticum* LMA28]	100	97	M	no data	no data
p2862_p2_28	*orf28*	30336	30575	hypothetical protein	hypothetical protein [*Carnobacterium divergens* TM377]	100	87	M	no data	no data
p2862_p2_27	*orf27*	30565	30732	hypothetical protein	ATP-binding protein [*Carnobacterium mobile* S2]	100	99	C	DnaC super family	DNA replication protein DnaC
p2862_p2_26	*dnaC*	30744	31583	DNA replication protein	phage replisome organizer N-terminal domain-containing protein [*Carnobacterium mobile* S2]	97	59	C	Phage_rep_org_N super family	N-terminal phage replisome organizer
p2862_p2_25	*orf25*	31561	32370	hypothetical protein	putative HNHc nuclease [*Carnobacterium maltaromaticum* LMA28]	100	99	C	HNHc_6 super family	Putative HNHc nuclease

* Abbreviations used in protein localization prediction: C- cytoplasm; M- membrane, E- extracellular

S1j. Annotation of the 2862_p2 plasmid of *C. maltaromaticum* strain 2862 (complement sequence, continued)

Tag	Gene	Position start	Position stop	Function	BLASTp best result	Identity (%)	Coverage (%)	Localization	Protein domain	Protein domain function
p2862_p2_24	*orf24*	32374	32787	hypothetical protein	hypothetical protein [*Carnobacterium maltaromaticum* LMA28]	100	97	M	no data	no data
p2862_p2_23	*orf23*	32878	33036	hypothetical protein	DUF1071 domain-containing protein [*Carnobacterium maltaromaticum* LMA28]	100	99	M	DUF1071 super family	Protein of unknown function (DUF1071)
p2862_p2_22	*orf22*	33033	33770	hypothetical protein	hypothetical protein [*Carnobacterium maltaromaticum* LMA28]	96	95	E	no data	no data
p2862_p2_21	*orf21*	33763	33963	hypothetical protein	hypothetical protein [*Carnobacterium maltaromaticum* LMA28]	97	80	E	no data	no data
p2862_p2_20	*orf20*	33956	34390	hypothetical protein	hypothetical protein [*Carnobacterium maltaromaticum* LMA28]	100	100	E	no data	no data
p2862_p2_19	*orf19*	34461	34619	hypothetical protein	hypothetical protein [*Carnobacterium maltaromaticum* LMA28]	100	96	M	no data	no data
p2862_p2_18	*orf18*	34612	34812	hypothetical protein	hypothetical protein [*Carnobacterium maltaromaticum* LMA28]	100	94	M	no data	no data
p2862_p2_17	*orf17*	34805	34978	hypothetical protein	hypothetical protein [*Vagococcus allomyrinae*]	84	45	M	PRK14559 super family	serine/threonine phosphatase
p2862_p2_16	*orf16*	35115	35414	hypothetical protein	hypothetical protein [*Carnobacterium maltaromaticum* LMA28]	100	70	M	no data	no data
p2862_p2_15	*orf15*	35411	35530	hypothetical protein	hypothetical protein [*Carnobacterium maltaromaticum* LMA28]	100	98	E	no data	no data

* Abbreviations used in protein localization prediction: C- cytoplasm; M- membrane, E- extracellular

S1j. Annotation of the 2862_p2 plasmid of *C. maltaromaticum* strain 2862 (complement sequence, continued)

Tag	Gene	Position		Function	BLASTp best result	Identity (%)	Coverage (%)	Localization	Protein domain	Protein domain function
		start	stop							
p2862_p2_14	*orf14*	35703	35966	hypothetical protein	KTSC domain-containing protein [*Carnobacterium maltaromaticum* LMA28]	100	98	M	no data	no data
p2862_p2_13	*orf13*	36060	36266	hypothetical protein	hypothetical protein [*Carnobacterium maltaromaticum* LMA28]	89	96	M	no data	no data
p2862_p2_12	*orf12*	36263	36439	hypothetical protein	hypothetical protein [*Carnobacterium maltaromaticum* LMA28]	100	98	M	no data	no data
p2862_p2_11	*orf11*	36453	37220	phage antirepressor	phage antirepressor KilAC domain-containing protein [*Clostridium tetani*]	98	66	M	ANT super family	Phage antirepressor protein KilAC domain
p2862_p2_10	*orf10*	37237	37485	hypothetical protein	Ltp family lipoprotein [*Carnobacterium maltaromaticum* LMA28]	100	97	M	Lipoprotein_Ltp	Host cell surface-exposed lipoprotein
p2862_p2_9	*hipB*	37653	38375	repressor	site-specific integrase [*Carnobacterium maltaromaticum* LMA28]	100	98	C	INT_ICEBs1_C_like	C-terminal catalytic domain of integrases from bacterial phages
p2862_p2_8	*orf8*	38469	39368	hypothetical protein	hypothetical protein [*Carnobacterium maltaromaticum* LMA28]	100	98	M	no data	no data
p2862_p2_7	*int*	39486	40628	integrase	site-specific integrase [*Carnobacterium maltaromaticum* LMA28]	100	98	C	INT_ICEBs1_C_like	C-terminal catalytic domain of integrases from bacterial phages
p2862_p2_6	*orf6*	40973	41338	hypothetical protein	hypothetical protein [*Carnobacterium maltaromaticum* LMA28]	100	98	M	no data	no data

* Abbreviations used in protein localization prediction: C- cytoplasm; M- membrane, E- extracellular

S1j. Annotation of the 2862_p2 plasmid of *C. maltaromaticum* strain 2862 (complement sequence, continued)

Tag	Gene	Position start	Position stop	Function	BLASTp best result	Identity (%)	Coverage (%)	Localization	Protein domain	Protein domain function
p2862_p2_5	*orf5*	41335	41607	hypothetical protein	hypothetical protein [*Carnobacterium maltaromaticum* LMA28]	100	96	M	no data	no data
p2862_p2_4	*orf4*	41595	41777	hypothetical protein	hypothetical protein [*Carnobacterium maltaromaticum* LMA28]	75	93	M	no data	no data
p2862_p2_3	*orf3*	41774	41926	hypothetical protein	ssDNA-binding protein [*Carnobacterium maltaromaticum* LMA28]	92	97	C	RPA_2b-aaRSs_OBF_like super family	Replication protein A
p2862_p2_2	*orf2*	41941	42384	hypothetical protein	hypothetical protein [*Enterococcus faecium*]	96	50	M	no data	no data
p2862_p2_1	*rep*	42377	43406	replication protein	replication protein [*Carnobacterium maltaromaticum* LMA28]	100	97	C	HTH super family	DNA binding domain

* Abbreviations used in protein localization prediction: C- cytoplasm; M- membrane, E- extracellular

S1k. Annotation of the 2862_p3 plasmid of *C. maltaromaticum* strain 2862 (complement sequence)

Tag	Gene	Position start	Position stop	Function	BLASTp best result	Identity (%)	Coverage (%)	Localization	Protein domain	Protein domain function
p2862_p3_78	*orf78*	1950	3206	transposase	IS110 family transposase [*Carnobacterium maltaromaticum* LMA28]	100	95	C	transpos_IS110 super family	IS110 family transposase
p2862_p3_77	*parB*	3536	4399	ATPase involved in chromosome partitioning	ParB family protein [*Carnobacterium maltaromaticum* LMA28]	100	99	C	BcsQ	Cellulose biosynthesis protein BcsQ
p2862_p3_76	*orf76*	4404	4811	hypothetical protein	hypothetical protein [*Carnobacterium maltaromaticum* LMA28]	91	92	M	no data	no data
p2862_p3_75	*orf75*	4857	5072	hypothetical protein	hypothetical protein [*Carnobacterium* sp. 17-4]	54	30	M	no data	no data
p2862_p3_74	*orf74*	5542	6372	transposase	IS3 family transposase [*Carnobacterium mobile* S2]	98	65	C	transpos_IS3	IS3 family transposase
p2862_p3_73	*orf73*	6378	6668	transposase	transposase [*Enterococcus saccharolyticus* subsp. *saccharolyticus* ATCC 4307]	100	76	C	transpos_IS3	IS3 family transposase
p2862_p3_72	*orf72*	6913	7440	hypothetical protein	super-infection exclusion protein B [*Carnobacterium maltaromaticum* LMA28]	100	73	M	no data	no data
p2862_p3_71	*orf71*	7682	8047	hypothetical protein	hypothetical protein [*Carnobacterium* sp. 17-4]	54	30	M	no data	no data
p2862_p3_70	*orf70*	8500	8766	hypothetical protein	transposase [*Carnobacterium mobile* S2]	100	75	C	transpos_IS3	IS3 family transposase
p2862_p3_69	*orf69*	9018	9713	transposase	IS3 family transposase [*Carnobacterium mobile* S2]	100	68	C	transpos_IS3	IS3 family transposase
p2862_p3_68	*orf68*	9893	10159	hypothetical protein	transposase [*Carnobacterium mobile* S2]	100	76	C	transpos_IS3	IS3 family transposase
p2862_p3_67	*ytgH*	10663	11205	transcriptional regulator	transcriptional regulator [*Carnobacterium mobile* S2]	92	54	C	Asp23 super family	cell envelope-related function

* Abbreviations used in protein localization prediction: C- cytoplasm; M- membrane, E- extracellular

S1k. Annotation of the 2862_p3 plasmid of *C. maltaromaticum* strain 2862 (complement sequence, continued)

Tag	Gene	Position		Function	BLASTp best result	Identity (%)	Coverage (%)	Localization	Protein domain	Protein domain function
		start	stop							
p2862_p3_66	*orf66*	11304	11480	hypothetical protein	DUF2273 domain-containing protein [*Apilactobacillus kunkee*i]	96	41	M	no data	no data
p2862_p3_65	*amaP*	11492	12067	alkaline shock response protein	alkaline shock response membrane anchor protein AmaP [*Carnobacterium mobile* S2]	90	32	M	DUF322 super family	Alakline shock response protein
p2862_p3_64	*orf64*	12426	12830	hypothetical protein	hypothetical protein [*Carnobacterium* sp. 17-4]	54	30	M	Gram_pos_anchor super family	LPXTG cell wall anchor motif
p2862_p3_63	*orf63*	12862	13425	hypothetical protein	hypothetical protein [*Carnobacterium mobile* S2]	96	50	M	no data	no data
p2862_p3_62	*orf62*	13473	13988	hypothetical protein	hypothetical protein [*Carnobacterium maltaromaticum* LMA28]	100	62	E	no data	no data
p2862_p3_61	*orf61*	14002	15801	hypothetical protein	BspA family leucine-rich repeat surface protein [*Carnobacterium maltaromaticum* LMA28]	90	33	M	DUF285 super family	Mycoplasma protein of unknown function
p2862_p3_60	*orf60*	15874	16119	hypothetical protein	hypothetical protein [*Carnobacterium maltaromaticum* LMA28]	91	45	M	no data	no data
p2862_p3_59	*orf59*	16443	16709	transposase	transposase [*Carnobacterium maltaromaticum* LMA28]	100	95	C	HTH super family	Helix-turn-helix domains
p2862_p3_58	*orf58*	16988	17527	hypothetical protein	conserved hypothetical protein [*Carnobacterium maltaromaticum* LMA28]	100	96	C	transpos_IS3	IS3 family transposase
p2862_p3_57	*orf57*	17660	17953	hypothetical protein	hypothetical protein [*Carnobacterium* sp. 17-4]	54	30	M	no data	no data
p2862_p3_56	*orf56*	19348	19971	hypothetical protein	helix-turn-helix transcriptional regulator [*Carnobacterium maltaromaticum* LMA28]	100	95	C	TPR_12 super family	Tetratricopeptide repeat

* Abbreviations used in protein localization prediction: C- cytoplasm; M- membrane, E- extracellular

S1k. Annotation of the 2862_p3 plasmid of *C. maltaromaticum* strain 2862 (complement sequence, continued)

Tag	Gene	Position		Function	BLASTp best result	Identity	Coverage	Localization	Protein domain	Protein domain function
		start	stop			(%)	(%)			
p2862_p3_55	*orf55*	20699	20887	hypothetical protein	hypothetical protein [*Carnobacterium maltaromaticum* LMA28]	100	90	M	PRK10787 super family	DNA-binding ATP-dependent protease
p2862_p3_54	*orf54*	20897	21121	hypothetical protein	hypothetical protein [*Carnobacterium maltaromaticum* LMA28]	700	97	M	no data	no data
p2862_p3_53	*orf53*	21189	21431	hypothetical protein	hypothetical protein [*Carnobacterium maltaromaticum* LMA28]	100	97	C	no data	no data
p2862_p3_52	*orf52*	21483	22178	transposase	IS3 family transposase [*Carnobacterium mobile* S2]	100	68	C	transpos_IS3	IS3 family transposase
p2862_p3_51	*orf51*	22358	22624	hypothetical protein	transposase [*Carnobacterium mobile* S2]	100	76	E	transpos_IS3	IS3 family transposase
p2862_p3_50	*orf50*	22696	22914	hypothetical protein	hypothetical protein [*Carnobacterium maltaromaticum* LMA28]	100	97	M	no data	no data
p2862_p3_49	*orf49*	22904	23515	resolvase	resolvase [*Carnobacterium maltaromaticum* LMA28]	100	97	C	SR_ResInv	Serine Recombinase (SR) family
p2862_p3_48	*orf48*	23764	24207	hypothetical protein	DUF5067 domain-containing protein [*Carnobacterium maltaromaticum* LMA28]	100	82	E	DUF4352 super family	Domain of unknown function (DUF4352)
p2862_p3_47	*yitT*	24518	25108	putative membrane spanning protein	YitT family protein [*Carnobacterium maltaromaticum* LMA28]	100	92	E	DUF6198 super family	Family of unknown function (DUF6198)
p2862_p3_46	*orf46*	25159	25923	hypothetical protein	hypothetical protein [*Carnobacterium mobile* S2]	100	56	E	PRiA4_ORF3 super family	Plasmid pRiA4b ORF-3-like protein
p2862_p3_45	*orf45*	26346	26498	hypothetical protein	hypothetical protein [*Carnobacterium maltaromaticum* LMA28]	100	100	E	no data	no data

* Abbreviations used in protein localization prediction: C- cytoplasm; M- membrane, E- extracellular

S1k. Annotation of the 2862_p3 plasmid of *C. maltaromaticum* strain 2862 (complement sequence, continued)

Tag	Gene	Position start	Position stop	Function	BLASTp best result	Identity (%)	Coverage (%)	Localization	Protein domain	Protein domain function
p2862_p3_44	*orf44*	26662	27030	hypothetical protein	hypothetical protein [*Carnobacterium gallinarum DSM4847*]	99	91	M	no data	no data
p2862_p3_43	*cro*	27066	27956	Cro/Cl family transcriptional regulator	helix-turn-helix transcriptional regulator [*Carnobacterium maltaromaticum* LMA28]	100	93	C	HTH_XRE super family	Helix-turn-helix XRE-family DNA binding domain
p2862_p3_42	*celB*	28411	29712	cellobiose-specific PTS system IIC component	PTS transporter subunit EIIC [*Carnobacterium maltaromaticum* LMA28]	100	97	C	CelB	Phosphotransferase system cellobiose-specific component IIC
p2862_p3_41	*gntR*	29753	30451	GntR family transcriptional regulator	GntR family transcriptional regulator [*Carnobacterium maltaromaticum* LMA28]	100	93	C	MngR	Helix-turn-helix DNA binding domains
p2862_p3_40	*orf40*	30757	31419	transposase	conserved hypothetical protein [*Carnobacterium maltaromaticum* LMA28]	100	92	C	transpos_IS3	IS3 family transposase
p2862_p3_39	*orf39*	31575	31841	transposase	recombinase family protein [*Carnobacterium maltaromaticum* LMA28]	100	97	C	HTH super family	Helix-turn-helix DNA binding domains
p2862_p3_38	*orf38*	31856	31990	hypothetical protein	hypothetical protein [*Carnobacterium gallinarum* DSM4847]	99	91	M	no data	no data
p2862_p3_37	*orf37*	31990	32193	hypothetical protein	hypothetical protein [*Carnobacterium gallinarum* DSM4847]	99	91	M	no data	no data
p2862_p3_36	*orf36*	32212	33168	hypothetical protein	hypothetical protein CN895_07965 [*Bacillus cereus*]	74	28	M	no data	no data
p2862_p3_35	*orf35*	33877	34002	hypothetical protein	hypothetical protein [*Carnobacterium mobile* S2]	90	86	M	no data	no data

* Abbreviations used in protein localization prediction: C- cytoplasm; M- membrane, E- extracellular

S1k. Annotation of the 2862_p3 plasmid of *C. maltaromaticum* strain 2862 (complement sequence, continued)

Tag	Gene	Position start	Position stop	Function	BLASTp best result	Identity (%)	Coverage (%)	Localization	Protein domain	Protein domain function
p2862_p3_34	*orf34*	34070	34336	hypothetical protein	transposase [*Carnobacterium mobile* S2]	100	76	C	transpos_IS3	IS3 family transposase
p2862_p3_33	*orf33*	34516	35211	transposase	IS3 family transposase [*Carnobacterium mobile* S2]	100	68	C	transpos_IS3	IS3 family transposase
p2862_p3_32	*orf32*	35247	35747	hypothetical protein	IS110 family transposase [*Carnobacterium maltaromaticum* LMA28]	97	98	C	transpos_IS110 super family	IS110 family transposas
p2862_p3_31	*orf31*	35878	36537	hypothetical protein	replication/maintenance protein RepL [*Carnobacterium mobile* S2]	49	31	C	no data	no data
p2862_p3_30	*mazF*	36640	36966	toxin-antitoxin addiction module toxin component MazF	type II toxin-antitoxin system PemK/MazF family toxin [*Carnobacterium maltaromaticum* LMA28]	96	88	C	PemK_toxin super family	PemK-like, MazF-like toxin of type II toxin-antitoxin system
p2862_p3_29	*abrB*	36966	37244	transcriptional regulator	AbrB/MazE/SpoVT family DNA-binding domain-containing protein [*Carnobacterium mobile* S2]	96	43	C	AbrB	Bifunctional DNA-binding transcriptional regulator of stationary/sporulation/toxin gene
p2862_p3_28	*orf28*	37309	37608	hypothetical protein	DNA topoisomerase III [*Carnobacterium mobile* S2]	90	48	C	Toprim_Crpt super family	C-terminal repeat of topoisomerase
p2862_p3_27	*orf27*	37688	37954	hypothetical protein	transposase [*Carnobacterium mobile* S2]	100	76	C	transpos_IS3 super family	IS3 family transposase
p2862_p3_26	*orf26*	38579	38962	hypothetical protein	IS256 family transposase [*Carnobacterium mobile* S2]	82	76	C	Transposase_mut super family	Transposase
p2862_p3_25	*orf25*	39068	39313	hypothetical protein	IS256 family transposase [*Carnobacterium mobile* S2]	80	88	C	Transposase_mut super family	Transposase
p2862_p3_24	*orf24*	39766	40605	hypothetical protein	hypothetical protein [*Carnobacterium maltaromaticum* LMA28]	100	96	M	no data	no data

* Abbreviations used in protein localization prediction: C- cytoplasm; M- membrane, E- extracellular

S1k. Annotation of the 2862_p3 plasmid of *C. maltaromaticum* strain 2862 (complement sequence, continued)

Tag	Gene	Position		Function	BLASTp best result	Identity (%)	Coverage (%)	Localization	Protein domain	Protein domain function
		start	stop							
p2862_p3_23	*orf23*	40741	40905	hypothetical protein	amidohydrolase [*Carnobacterium maltaromaticum* LMA28]	96	84	C	metallo-dependent_hydrolases super family	Superfamily of metallo-dependent hydrolases
p2862_p3_22	*orf22*	41139	41801	transposase	transposase [*Carnobacterium maltaromaticum* LMA28]	100	92	C	transpos_IS3	IS3 family transposase
p2862_p3_21	*orf21*	42041	42223	hypothetical protein	transposase [*Carnobacterium maltaromaticum* LMA28]	100	95	C	HTH super family	Helix-turn-helix DNA binding domains
p2862_p3_20	*parA*	42275	42811	hypothetical protein	replication initiator protein A [*Carnobacterium maltaromaticum* LMA28]	98	80	C	HTH super family	Helix-turn-helix DNA binding domains
p2862_p3_19	*orf19*	43305	44204	ATPase involved in chromosome partitioning	ParA superfamily DNA segregation protein PrgP [*Carnobacterium maltaromaticum* LMA28]	100	100	C	P-loop_NTPase super family	P-loop containing nucleoside triphosphate hydrolases
p2862_p3_18	*orf18*	44221	44490	hypothetical protein	hypothetical protein [*Carnobacterium maltaromaticum* LMA28]	100	100	M	no data	no data
p2862_p3_17	*tnpR*	45466	46059	resolvase	resolvase [*Carnobacterium maltaromaticum* LMA28]	97	93	C	SR_ResInv	Serine Recombinase (SR) family
p2862_p3_16	*orf16*	46248	47087	hypothetical protein	Tn3 family transposase [*Carnobacterium maltaromaticum* LMA28]	100	91	C	DUF4158 super family	Domain of unknown function (DUF4158)
p2862_p3_15	*orf15*	47210	49249	hypothetical protein	Tn3 family transposase [*Carnobacterium maltaromaticum* LMA28]	100	96	C	DDE_Tnp_Tn3 super family	Tn3 transposase DDE domain
p2862_p3_14	*orf14*	49362	49931	hypothetical protein	IS3 family transposase [*Carnobacterium mobile* S2]	91	87	C	transpos_IS3	IS3 family transposase

* Abbreviations used in protein localization prediction: C- cytoplasm; M- membrane, E- extracellular

S1k. Annotation of the 2862_p3 plasmid of *C. maltaromaticum* strain 2862 (complement sequence, continued)

Tag	Gene	Position		Function	BLASTp best result	Identity (%)	Coverage (%)	Localization	Protein domain	Protein domain function
		start	stop							
p2862_p3_13	*orf13*	50111	50377	hypothetical protein	transposase [*Carnobacterium mobile* S2]	100	76	C	transpos_IS3	IS3 family transposase
p2862_p3_12	*orf12*	50432	50971	hypothetical protein	type IV secretory system conjugative DNA transfer family protein [*Carnobacterium maltaromaticum* LMA28]	97	95	M	no data	no data
p2862_p3_11	*orf11*	51190	51810	transposase	transposase [*Carnobacterium maltaromaticum* LMA28]	100	93	C	transpos_IS3	IS3 family transposase
p2862_p3_10	*orf10*	52008	52274	transposase	transposase [*Carnobacterium maltaromaticum* LMA28]	100	90	C	HTH super family	Helix-turn-helix DNA binding domains
p2862_p3_9	*orf9*	52414	53124	hypothetical protein	hypothetical protein [*Carnobacterium maltaromaticum* DSM 20342]	99	89	M	no data	no data
p2862_p3_8	*orf8*	53195	53461	transposase	transposase [*Carnobacterium maltaromaticum* LMA28]	100	90	C	HTH super family	Helix-turn-helix DNA binding domains
p2862_p3_7	*orf7*	53659	54279	transposase	Transposase [*Carnobacterium maltaromaticum* LMA28]	100	95	C	transpos_IS3	IS3 family transposase
p2862_p3_6	*orf6*	54417	54737	hypothetical protein	conserved hypothetical protein [*Carnobacterium maltaromaticum* LMA28]	91	87	C	AcrIIA1 super family	Anti-CRISPR type II subtype A1
p2862_p3_5	*rep*	55034	56584	plasmid replication protein	primase C-terminal domain-containing protein [*Carnobacterium maltaromaticum* LMA28]	100	93	C	PriCT_1 super family	Primase C terminal 1

* Abbreviations used in protein localization prediction: C- cytoplasm; M- membrane, E- extracellular

S1k. Annotation of the 2862_p3 plasmid of *C. maltaromaticum* strain 2862 (complement sequence, continued)

Tag	Gene	Position		Function	BLASTp best result	Identity	Coverage	Localization	Protein domain	Protein domain function
		start	stop			(%)	(%)			
p2862_p3_4	*orf4*	57134	57496	hypothetical protein	hypothetical protein [*Enterococcus faecalis*]	90	46	M	no data	no data
p2862_p3_3	*parA*	57489	58295	chromosome partitioning protein ParA	partition protein ParA [*Lactobacillaceae bacterium* WR16-4]	95	48	C	P-loop_NTPase super family	P-loop containing nucleoside triphosphate hydrolases
p2862_p3_2	*orf2*	58747	59088	hypothetical protein	hypothetical protein [*Carnobacterium mobile* S2]	98	41	M	no data	no data
p2862_p3_1	*repB*	59250	60935	repliction protein RepB	replication protein [*Carnobacterium maltaromaticum* LMA28]	100	96	C	HTH super family	Helix-turn-helix DNA binding domains

* Abbreviations used in protein localization prediction: C- cytoplasm; M- membrane, E- extracellular

S1l. Annotation of the 2862_p4 plasmid of *C. maltaromaticum* strain 2862 (complement sequence)

Tag	Gene	Position		Function	BLASTp best result	Identity	Coverage	Localization	Protein domain	Protein domain function
		start	stop			(%)	(%)			
p2862_p4_85	*orf85*	259	402	hypothetical protein	hypothetical protein [*Carnobacterium* sp. 17-4]	100	97	M	no data	no data
p2862_p4_84	*orf84*	419	601	hypothetical protein	hypothetical protein [*Carnobacterium maltaromaticum* LMA28]	100	97	E	no data	no data
p2862_p4_83	*orf83*	634	840	hypothetical protein	hypothetical protein [*Carnobacterium* sp. 17-4]	100	100	M	no data	no data
p2862_p4_82	*orf82*	915	1223	hypothetical protein	hypothetical protein IV70_GL000464 [*Carnobacterium maltaromaticum* DSM 20342]	98	98	M	DUF1433 super family	Protein of unknown function
p2862_p4_81	*orf81*	1350	2531	hypothetical protein	hypothetical protein [*Carnobacterium maltaromaticum* LMA28]	100	97	C	Abhydrolase super family	alpha/beta hydrolases
p2862_p4_80	*orf80*	2533	2919	hypothetical protein	hypothetical protein [*Carnobacterium* sp. 17-4]	100	100	M	no data	no data
p2862_p4_79	*orf79*	3206	3748	hypothetical protein	helix-turn-helix transcriptional regulator [*Carnobacterium maltaromaticum* LMA28]	96	97	C	HTH_XRE	Prokaryotic DNA binding proteins belonging to the xenobiotic response element family of transcriptional regulators
p2862_p4_78	*orf78*	3774	4457	transposase	IS3 family transposase [*Carnobacterium* sp. 17-4]	99	69	C	transpos_IS3 super family	IS3 family transposase
p2862_p4_77	*orf77*	4649	4915	hypothetical protein	transposase [*Carnobacterium* sp. 17-4]	100	76	C	transpos_IS3 super family	IS3 family transposase
p2862_p4_76	*orf76*	4960	5355	hypothetical protein	helix-turn-helix transcriptional regulator [*Carnobacterium maltaromaticum* LMA28]	93	94	C	no data	no data
p2862_p4_75	*orf75*	5511	6854	hypothetical protein	FRG domain-containing protein [*Carnobacterium* sp. 17-4]	96	37	M	FRG super family	FRG domain, functionally uncharacterized

* Abbreviations used in protein localization prediction: C- cytoplasm; M- membrane, E- extracellular

S1l. Annotation of the 2862_p4 plasmid of *C. maltaromaticum* strain 2862 (complement sequence, continued)

Tag	Gene	Position		Function	BLASTp best result	Identity	Coverage	Localization	Protein domain	Protein domain function
		start	stop			(%)	(%)			
p2862_p4_74	*orf74*	6980	7729	hypothetical protein	hypothetical protein [*Carnobacterium* sp. 17-4]	85	65	E	DUF5677 super family	Family of unknown function
p2862_p4_73	*orf73*	7818	8168	hypothetical protein	hypothetical protein [*Carnobacterium* sp. 17-4]	54	30	E	no data	no data
p2862_p4_72	*tnpR*	8378	8986	resolvase	resolvase protein [*Carnobacterium maltaromaticum* LMA28]	99	99	C	SR_ResInv	Resolvase and Invertase subfamily, catalytic domain
p2862_p4_71	*orf71*	9092	9343	hypothetical protein	hypothetical protein [*Carnobacterium maltaromaticum* LMA28]	99	88	E	no data	no data
p2862_p4_70	*orf70*	9393	9590	hypothetical protein	hypothetical protein [*Carnobacterium maltaromaticum* LMA28]	100	94	E	no data	no data
p2862_p4_69	*orf69*	10162	10845	transposase	transposase [*Carnobacterium* sp. 17-4]	100	93	C	PriCT_1 super family	Primase C terminal 1 (PriCT-1)
p2862_p4_68	*orf68*	11037	11303	hypothetical protein	hypothetical protein [*Carnobacterium maltaromaticum* LMA28]	99	92	M	AcrllA1 super family	Anti-CRISPR type II subtype A1
p2862_p4_67	*orf67*	12548	12916	hypothetical protein	helix-turn-helix transcriptional regulator [*Carnobacterium maltaromaticum* LMA28]	100	95	C	HTH_XRE	Helix-turn-helix XRE-family DNA binding domain
p2862_p4_66	*parA*	12916	13788	chromosome partitioning protein ParA	chromosome partitioning protein [*Carnobacterium maltaromaticum* LMA28]	100	95	C	HTH super family	Helix-turn-helix DNA binding domain
p2862_p4_65	*rep*	14436	15986	plasmid replication protein	hypothetical protein [*Carnobacterium maltaromaticum* LMA28]	94	83	C	HTH_XRE	Helix-turn-helix XRE-family DNA binding domain
p2862_p4_64	*orf64*	16187	16750	hypothetical protein	hypothetical protein [*Carnobacterium maltaromaticum* LMA28]	99	64	E	no data	no data

* Abbreviations used in protein localization prediction: C- cytoplasm; M- membrane, E- extracellular

S1l. Annotation of the 2862_p4 plasmid of *C. maltaromaticum* strain 2862 (complement sequence, continued)

Tag	Gene	Position		Function	BLASTp best result	Identity (%)	Coverage (%)	Localization	Protein domain	Protein domain function
		start	stop							
p2862_p4_63	*croI*	17067	17954	CroI family transcriptional regulator	transcriptional regulator [*Carnobacterium maltaromaticum* LMA28]	35	55	C	Cro_N super family	Bacterial regulatory proteins, cro family
p2862_p4_62	*orf62*	18651	18944	hypothetical protein	hypothetical protein [*Carnobacterium maltaromaticum* LMA28]	100	52	M	no data	no data
p2862_p4_61	*orf61*	19358	19576	hypothetical protein	hypothetical protein [*Carnobacterium maltaromaticum* LMA28]	100	95	M	no data	no data
p2862_p4_60	*orf60*	19598	19987	hypothetical protein	hypothetical protein [*Carnobacterium maltaromaticum* LMA28]	100	88	M	Endotoxin_C2 super family	Delta endotoxin
p2862_p4_59	*orf59*	19974	20126	hypothetical protein	SpaA isopeptide-forming pilin-related protein [*Carnobacterium maltaromaticum* LMA28]	100	95	M	Gram_pos_anchor super family	LPXTG cell wall anchor motif
p2862_p4_58	*orf58*	20386	21051	hypothetical protein	hypothetical protein [*Carnobacterium maltaromaticum* LMA28]	100	88	E	no data	no data
p2862_p4_57	*orf57*	21105	21308	hypothetical protein	hypothetical protein [*Carnobacterium maltaromaticum* LMA28]	35	55	M	no data	no data
p2862_p4_56	*orf56*	21484	22596	hypothetical protein	type III secretion system protein PrgE [*Carnobacterium maltaromaticum* LMA28]	100	97	M	no data	no data
p2862_p4_55	*orf55*	22732	25554	cell wall surface anchor protein	hypothetical protein [*Carnobacterium maltaromaticum* LMA28]	35	55	M	no data	no data
p2862_p4_54	*orf54*	25619	25999	hypothetical protein	hypothetical protein [*Carnobacterium maltaromaticum* LMA28]	40	50	M	no data	no data

* Abbreviations used in protein localization prediction: C- cytoplasm; M- membrane, E- extracellular

S1l. Annotation of the 2862_p4 plasmid of *C. maltaromaticum* strain 2862 (complement sequence, continued)

Tag	Gene	Position start	Position stop	Function	BLASTp best result	Identity (%)	Coverage (%)	Localization	Protein domain	Protein domain function
p2862_p4_53	*orf53*	26009	26224	hypothetical protein	type III secretion system protein PrgF [*Carnobacterium maltaromaticum* LMA28]	100	87	M	no data	no data
p2862_p4_52	*orf52*	26574	27011	hypothetical protein	type IV secretion system protein [*Carnobacterium maltaromaticum* LMA28]	100	97	E	TrbL super family	TrbL/VirB6 plasmid conjugal transfer protein
p2862_p4_51	*orf51*	27036	27179	hypothetical protein	PrgI family protein [*Carnobacterium maltaromaticum* LMA28]	100	86	E	PrgI super family	This family of proteins is functionally uncharacterized
p2862_p4_50	*traC*	27201	27365	conjugal protein	TraC-F-type conjugal transfer protein [*Carnobacterium maltaromaticum* LMA28]	98	96	C	TraC-F-type super family	type-IV secretion system protein TraC
p2862_p4_49	*orf49*	27366	27593	hypothetical protein	phage tail tip lysozyme [*Carnobacterium maltaromaticum* LMA28]	100	85	C	Phage_lysozyme2 super family	Phage tail lysozyme
p2862_p4_48	*prgH*	27609	28409	PrgH protein	PrgH protein [*Carnobacterium maltaromaticum* LMA28]	100	88	E	no data	no data
p2862_p4_47	*prgI*	28411	28764	PrgI protein	type III secretion system protein PrgI [*Carnobacterium maltaromaticum* LMA28]	100	90	C	PRK12704 super family	phosphodiesterase
p2862_p4_46	*orf46*	28718	31099	hypothetical protein	hypothetical protein [*Carnobacterium maltaromaticum* LMA28]	100	94	M	Smc super family	Chromosome segregation ATPase
p2862_p4_45	*orf45*	31112	33721	hypothetical protein	hypothetical protein [*Carnobacterium maltaromaticum* LMA28]	100	98	E	no data	no data
p2862_p4_44	*orf44*	33771	34466	hypothetical protein	hypothetical protein [*Carnobacterium maltaromaticum* LMA28]	100	75	M	no data	no data

* Abbreviations used in protein localization prediction: C- cytoplasm; M- membrane, E- extracellular

S1l. Annotation of the 2862_p4 plasmid of *C. maltaromaticum* strain 2862 (complement sequence, continued)

Tag	Gene	Position start	Position stop	Function	BLASTp best result	Identity (%)	Coverage (%)	Localization	Protein domain	Protein domain function
p2862_p4_43	*orf43*	34438	34725	hypothetical protein	hypothetical protein [*Carnobacterium maltaromaticum* LMA28]	100	89	M	no data	no data
p2862_p4_42	*orf42*	34727	36043	hypothetical protein	hypothetical protein [*Carnobacterium maltaromaticum* LMA28]	100	95	M	no data	no data
p2862_p4_41	*orf41*	36171	36539	hypothetical protein	hypothetical protein [*Carnobacterium maltaromaticum* LMA28]	65	35	M	no data	no data
p2862_p4_40	*orf40*	36644	36985	hypothetical protein	hypothetical protein [*Carnobacterium maltaromaticum* LMA28]	100	94	E	no data	no data
p2862_p4_39	*orf39*	37082	37582	hypothetical protein	hypothetical protein [*Carnobacterium maltaromaticum* LMA28]	100	94	M	PB1 super family	PB1 domain involved in signal transduction
p2862_p4_38	*orf38*	37582	37842	hypothetical protein	hypothetical protein [*Carnobacterium maltaromaticum* LMA28]	90	65	M	no data	no data
p2862_p4_37	*orf37*	37917	38309	hypothetical protein	AbrB family transcriptional regulator [unclassified *Enterococcus*]	100	71	M	no data	no data
p2862_p4_36	*orf36*	38318	38671	hypothetical protein	type II toxin-antitoxin system PemK/MazF family toxin [*Carnobacterium mobile* S2]	100	81	C	PemK_toxin super family	PemK-like, MazF-like toxin of type II toxin-antitoxin system
p2862_p4_35	*orf35*	38688	39071	hypothetical protein	hypothetical protein [*Carnobacterium maltaromaticum* LMA28]	65	35	M	no data	no data
p2862_p4_34	*orf34*	39128	39511	hypothetical protein	hypothetical protein [*Carnobacterium maltaromaticum* LMA28]	65	35	E	no data	no data

* Abbreviations used in protein localization prediction: C- cytoplasm; M- membrane, E- extracellular

S1l. Annotation of the 2862_p4 plasmid of *C. maltaromaticum* strain 2862 (complement sequence, continued)

Tag	Gene	Position start	stop	Function	BLASTp best result	Identity (%)	Coverage (%)	Localization	Protein domain	Protein domain function
p2862_p4_33	*orf33*	39584	39805	transposase	IS3 family transposase [*Carnobacterium mobile* S2]	99	69	C	transpos_IS3 super family	IS3 family transposase
p2862_p4_32	*mazF*	39805	40179	toxin-antitoxin addiction module toxin component MazF	type II toxin-antitoxin system PemK/MazF family toxin [unclassified *Enterococcus*]	100	81	C	PemK_toxin super family	PemK-like, MazF-like toxin of type II toxin-antitoxin system
p2862_p4_31	*orf31*	40240	40500	hypothetical protein	type IV secretory system conjugative DNA transfer family protein [*Carnobacterium maltaromaticum* LMA28]	100	98	M	T4SS-DNA_transf super family	Type IV secretory system Conjugative DNA transfer
p2862_p4_30	*orf30*	40564	40884	hypothetical protein	toprim domain-containing protein [*Carnobacterium maltaromaticum* LMA28]	98	86	E	TOPRIM super family	Topoisomerase-primase domain
p2862_p4_29	*orf29*	40929	41612	transposase	transposase [*Carnobacterium maltaromaticum* LMA28]	100	97	C	transpos_IS3 super family	IS3 family transposase
p2862_p4_28	*orf28*	41804	42070	hypothetical protein	conjugal protein [*Carnobacterium maltaromaticum* LMA28]	100	98	M	no data	no data
p2862_p4_27	*traG*	42314	44131	conjugal transfer protein TraG	plasmid transfer protein TraG [*Carnobacterium maltaromaticum* LMA28]	98	87	C	MobC super family	Bacterial mobilisation protein (MobC)
p2862_p4_26	*orf26*	44228	48055	relaxase	relaxase/mobilization nuclease domain-containing protein [*Carnobacterium maltaromaticum* LMA28]	100	98	C	Relaxase super family	Relaxase/Mobilisation nuclease domain
p2862_p4_25	*orf25*	48102	49211	hypothetical protein	hypothetical protein [*Carnobacterium maltaromaticum* LMA28]	90	91	M	no data	no data

* Abbreviations used in protein localization prediction: C- cytoplasm; M- membrane, E- extracellular

S1l. Annotation of the 2862_p4 plasmid of *C. maltaromaticum* strain 2862 (complement sequence, continued)

Tag	Gene	Position		Function	BLASTp best result	Identity (%)	Coverage (%)	Localization	Protein domain	Protein domain function
		start	stop							
p2862_p4_24	*orf24*	49208	49510	transposase	transposase [*Enterococcus saccharolyticus* subsp. *saccharolyticus* ATCC 4307]	100	77	C	transpos_IS3 super family	IS3 family transposase;
p2862_p4_23	*mobA*	49741	50100	mobilization protein	mobilization protein [*Carnobacterium mobile* S2]	98	64	C	MobL super family	MobL relaxases
p2862_p4_22	*greAB*	50100	51779	elongation factor GreAB	elongation factor GreAB [*Carnobacterium maltaromaticum* LMA28]	100	93	C	AdoMet_MTases super family	S-adenosylmethionine-dependent methyltransferases
p2862_p4_21	*orf21*	51821	52255	hypothetical protein	DNA (cytosine-5-)-methyltransferase [*Carnobacterium maltaromaticum* LMA28]	100	93	M	AdoMet_MTases super family	S-adenosylmethionine-dependent methyltransferases
p2862_p4_20	*orf20*	52329	52619	transposase	transposase [*Carnobacterium maltaromaticum* LMA28]	100	96	C	AdoMet_MTases super family	S-adenosylmethionine-dependent methyltransferases
p2862_p4_19	*orf19*	52616	53455	transposase	transposase [*Carnobacterium maltaromaticum* LMA28]	100	100	C	PhdYeFM_antitox super family	Antitoxin Phd_YefM, type II toxin-antitoxin system
p2862_p4_18	*parE*	53666	54130	partition protein	Txe/YoeB family addiction module toxin [*Carnobacterium maltaromaticum* LMA28]	100	96	C	ParE_toxin super family	ParE toxin of type II toxin-antitoxin system, parDE
p2862_p4_17	*orf17*	54247	55740	hypothetical protein	hypothetical protein [*Carnobacterium maltaromaticum* LMA28]	100	98	M	no data	no data
p2862_p4_16	*orf16*	55914	56669	hypothetical protein	hypothetical protein [*Carnobacterium maltaromaticum* LMA28]	65	35	M	no data	no data
p2862_p4_15	*orf15*	56770	57018	hypothetical protein	hypothetical protein [*Carnobacterium maltaromaticum* LMA28]	98	91	M	no data	no data

* Abbreviations used in protein localization prediction: C- cytoplasm; M- membrane, E- extracellular

S1l. Annotation of the 2862_p4 plasmid of *C. maltaromaticum* strain 2862 (complement sequence, continued)

Tag	Gene	Position		Function	BLASTp best result	Identity	Coverage	Localization	Protein domain	Protein domain function
		start	stop			(%)	(%)			
p2862_p4_14	*yoeB*	57020	57307	Txe/YoeB family addiction module toxin	toxin protein [*Carnobacterium maltaromaticum* LMA28]	100	85	C	YoeB_toxin super family	YoeA-like, Txe-like toxin of type II toxin-antitoxin system
p2862_p4_13	*orf13*	57375	57608	hypothetical protein	thermonuclease family protein [*Carnobacterium maltaromaticum* LMA28]	100	91	C	SNc super family	*Staphylococcal* nuclease homologues
p2862_p4_12	*orf12*	57723	57857	hypothetical protein	hypothetical protein [*Carnobacterium maltaromaticum* LMA28]	100	93	M	no data	no data
p2862_p4_11	*orf11*	57940	58278	hypothetical protein	hypothetical protein [*Carnobacterium maltaromaticum* LMA28]	100	95	M	no data	no data
p2862_p4_10	*orf10*	58552	58875	hypothetical protein	hypothetical protein [*Carnobacterium maltaromaticum* LMA28]	100	92	M	no data	no data
p2862_p4_9	*dcm*	59075	59689	thermonuclease precursor	DNA cytosine methyltransferase [*Carnobacterium maltaromaticum* LMA28]	100	96	C	Dcm super family	Site-specific DNA-cytosine methylase
p2862_p4_8	*orf8*	59721	60164	hypothetical protein	DNA cytosine methyltransferase [*Carnobacterium maltaromaticum* LMA28]	100	96	M	AdoMet_MTases super family	S-adenosylmethionine-dependent methyltransferases
p2862_p4_7	*orf7*	60234	60509	hypothetical protein	hypothetical protein CMALT394_600003 [*Carnobacterium maltaromaticum* LMA28]	64	87	M	no data	no data
p2862_p4_6	*orf6*	60542	60766	hypothetical protein	IS110 family transposase [*Carnobacterium maltaromaticum* LMA28]	100	95	C	transpos_IS110 super family	IS110 family transposase
p2862_p4_5	*orf5*	60786	61358	hypothetical protein	DNA cytosine methyltransferase [*Carnobacterium maltaromaticum* LMA28]	100	96	C	Dcm super family	Site-specific DNA-cytosine methylase

* Abbreviations used in protein localization prediction: C- cytoplasm; M- membrane, E- extracellular

S1l. Annotation of the 2862_p4 plasmid of C. maltaromaticum strain 2862 (complement sequence, continued)

Tag	Gene	Position start	Position stop	Function	BLASTp best result	Identity (%)	Coverage (%)	Localization	Protein domain	Protein domain function
p2862_p4_4	*orf4*	61503	62270	hypothetical protein	DNA cytosine methyltransferase [*Carnobacterium maltaromaticum* LMA28]	100	96	C	AdoMet_MTases super family	S-adenosylmethionine-dependent methyltransferases
p2862_p4_3	*orf3*	62270	62644	hypothetical protein	hypothetical protein CMALT394_600003 [*Carnobacterium maltaromaticum* LMA28]	64	87	E	no data	no data
p2862_p4_2	*orf2*	62963	64219	transposase	IS110 family transposase [*Carnobacterium maltaromaticum* LMA28]	100	95	C	transpos_IS110 super family	IS110 family transposase
p2862_p4_1	*repB*	64612	65928	repliction protein RepB	replication protein B [*Carnobacterium maltaromaticum* LMA28]	100	96	C	HTH super family	Helix-turn-helix DNA binding domain

* Abbreviations used in protein localization prediction: C- cytoplasm; M- membrane, E- extracellular

S1m. Annotation of the 2862_p5 plasmid of *C. maltaromaticum* strain 2862

Tag	Gene	Position start	Position stop	Function	BLASTp best result	Identity (%)	Coverage (%)	Localization	Protein domain	Protein domain function
p2862_p5_1	*rep*	819	2399	plasmid replication protein	replication protein Rep [*Carnobacterium maltaromaticum* LMA28]	97	85	C	Rep_3	Initiator Replication protein RepB
p2862_p5_2	*orf2*	2756	3085	hypothetical protein	C-terminal domain-containing primase [*Carnobacterium maltaromaticum* LMA28]	100	97	E	PriCT_1 super family	Primase C terminal 1 (PriCT-1)
p2862_p5_3	*parA*	3457	4419	chromosome partitioning protein ParA	partition protein [*Carnobacterium* sp. 17-4]	100	100	C	HTH super family	Helix-turn-helix DNA binding domain
p2862_p5_4	*orf4*	4419	4892	hypothetical protein	hypothetical protein [*Carnobacterium* sp. 17-4]	100	100	E	P-loop_NTPase super family	P-loop containing Nucleoside Triphosphate Hydrolase
p2862_p5_5	*orf5*	5249	5932	transposase	hypothetical protein [*Carnobacterium* sp. 17-4]	100	96	C	transpos_IS3 super family	IS3 family transposase
p2862_p5_6	*orf6*	6091	6357	hypothetical protein	IS3 family transposase [*Carnobacterium inhibens* WN1359]	99	68	C	transpos_IS3 super family	IS3 family transposase
p2862_p5_7	*orf7*	6549	7232	transposase	transposase [*Carnobacterium* sp. 17-4]	100	76	C	transpos_IS3 super family	IS3 family transposase
p2862_p5_8	*orf8*	7374	7640	hypothetical protein	IS3 family transposase [*Carnobacterium inhibens* WN1359]	97	65	C	transpos_IS3 super family	IS3 family transposase
p2862_p5_9	*xerS*	9156	10229	tyrosine recombinase XerS	transposase [*Carnobacterium inhibens* WN1359]	100	76	C	transpos_IS3 super family	IS3 family transposase
p2862_p5_10	*orf10*	10483	10794	hypothetical protein	tyrosine recombinase XerS [*Carnobacterium maltaromaticum* LMA28]	100	99	M	xerS	site-specific tyrosine recombinase XerS
p2862_p5_11	*orf11*	10787	10930	hypothetical protein	type II toxin-antitoxin system RelB/DinJ family antitoxin [*Carnobacterium inhibens* WN1359]	99	84	M	RelB super family	RelB antitoxin; RelE and RelB form a toxin-antitoxin system

* Abbreviations used in protein localization prediction: C- cytoplasm; M- membrane, E- extracellular

S1m. Annotation of the 2862_p5 plasmid of *C. maltaromaticum* strain 2862 (continued)

Tag	Gene	Position		Function	BLASTp best result	Identity	Coverage	Localization	Protein domain	Protein domain function
		start	stop			(%)	(%)			
p2862_p5_12	*orf12*	10920	11105	hypothetical protein	Select seq ref|WP_142967480.1| type II toxin-antitoxin system RelE/ParE family toxin [*Carnobacterium inhibens* WN1359]	91	61	M	no data	no data
p2862_p5_13	*orf13*	11769	12035	hypothetical protein	transposase [*Carnobacterium* sp. 17-4]	100	76	C	transpos_IS3 super family	IS3 family transposase
p2862_p5_14	*orf14*	12428	13111	transposase	transposase [*Carnobacterium* sp. 17-4]	99	60	C	transpos_IS3 super family	IS3 family transposase
p2862_p5_15	*orf15*	13303	13569	hypothetical protein	IS3 family transposase [*Latilactobacillus curvatus* CRL 705]	100	76	C	transpos_IS3 super family	IS3 family transposase
p2862_p5_16	*mazF*	13614	13907	type II toxin-antitoxin system PemK/MazF	type II toxin-antitoxin protein [*Carnobacterium maltaromaticum* LMA28]	99	65	C	PemK_toxin super family	PemK-like, MazF-like toxin of type II toxin-antitoxin system
p2862_p5_17	*orf17*	14322	15317	hypothetical protein	hypothetical protein [*Carnobacterium maltaromaticum* LMA28]	99	98	E	no data	no data
p2862_p5_18	*atpD*	15554	16180	V-type ATP synthase subunit D	V-type ATP synthase subunit D [*Carnobacterium maltaromaticum* LMA28]	100	99	C	ATP-synt_D super family	ATP synthase subunit D
p2862_p5_19	*atpB*	16188	17564	V-type ATP synthase subunit B	V-type ATP synthase subunit B [*Carnobacterium maltaromaticum* LMA28]	100	100	C	PRK04196	V-type ATP synthase subunit B
p2862_p5_20	*atpA*	17557	19341	V-type ATP synthase alpha chain	V-type ATP synthase subunit A [*Carnobacterium maltaromaticum* LMA28]	100	99	C	PRK04192	V-type ATP synthase subunit A
p2862_p5_21	*atpF*	19357	19668	V-type ATP synthase subunit F	V-type ATP synthase subunit A [*Carnobacterium maltaromaticum* LMA28]	100	99	C	ATP-synt_F super family	ATP synthase (F/14-kDa) subunit

* Abbreviations used in protein localization prediction: C- cytoplasm; M- membrane, E- extracellular

S1m. Annotation of the 2862_p5 plasmid of *C. maltaromaticum* strain 2862 (continued)

Tag	Gene	Position		Function	BLASTp best result	Identity	Coverage	Localization	Protein domain	Protein domain function
		start	stop			(%)	(%)			
p2862_p5_22	*atpC*	19658	20662	V-type ATP synthase subunit C	V-type ATPase subunit [*Carnobacterium maltaromaticum* LMA28]	100	76	C	vATP-synt_AC39 super family	ATP synthase (C/AC39) subunit
p2862_p5_23	*atpE*	20680	21264	V-type ATP synthase subunit E	V-type ATP synthase subunit E [*Carnobacterium maltaromaticum* LMA28]	100	95	C	NtpE super family	ATPase subunit E/Vma4
p2862_p5_24	*atpK*	21296	21772	V-type ATP synthase subunit K	V-type ATP synthase subunit K [*Carnobacterium maltaromaticum* LMA28]	100	99	C	PRK06558	V-type ATP synthase subunit K
p2862_p5_25	*atpI*	21836	23806	V-type ATP synthase subunit I	V-type ATP synthase subunit I [*Carnobacterium maltaromaticum* LMA28]	100	85	C	PRK05771	V-type ATP synthase subunit I
p2862_p5_26	*orf26*	23793	24119	hypothetical protein	hypothetical protein IV70_GL002486 [*Carnobacterium maltaromaticum* DSM 20342]	100	74	M	no data	no data
p2862_p5_27	*orf27*	24357	25040	transposase	IS3 family transposase [*Carnobacterium* sp. 17-4]	99	69	C	transpos_IS3 super family	IS3 family transposase;
p2862_p5_28	*orf28*	25232	25498	transposase	transposase [*Carnobacterium* sp. 17-4]	100	76	C	transpos_IS3 super family	IS3 family transposase;
p2862_p5_29	*tetR*	25561	26175	TetR family transcriptional regulator	helix-turn-helix domain-containing protein [*Carnobacterium maltaromaticum* LMA28]	94	93	C	TetR_N super family	Bacterial regulatory proteins, tetR family
p2862_p5_30	*orf30*	26477	26842	hypothetical protein	hypothetical protein [*Carnobacterium maltaromaticum* LMA28]	99	98	E	no data	no data
p2862_p5_31	*orf31*	27069	28895	extracellular protein	exopolysaccharide [*Carnobacterium funditum* DSM5970]	100	38	E	LPS_length_determ_N_term	Polysaccharide chain length determinant N-terminal

* Abbreviations used in protein localization prediction: C- cytoplasm; M- membrane, E- extracellular

S1m. Annotation of the 2862_p5 plasmid of *C. maltaromaticum* strain 2862 (continued)

Tag	Gene	Position start	Position stop	Function	BLASTp best result	Identity (%)	Coverage (%)	Localization	Protein domain	Protein domain function
p2862_p5_32	*orf32*	29105	29725	hypothetical protein	WxL domain-containing protein [*Carnobacterium maltaromaticum* LMA28]	100	49	M	WxL super family	WxL domain surface cell wall-binding
p2862_p5_33	*orf33*	29974	30633	hypothetical protein	DUF3324 domain-containing protein [*Carnobacterium maltaromaticum* LMA28]	94	61	E	DUF3324 super family	Protein of unknown function C-terminal
p2862_p5_34	*orf34*	30802	31485	transposase	IS3 family transposase [*Carnobacterium inhibens* WN1359]	99	69	C	transpos_IS3 super family	IS3 family transposase
p2862_p5_35	*orf35*	31677	31943	hypothetical protein	transposase [*Carnobacterium inhibens* WN1359]	100	76	C	transpos_IS3 super family	IS3 family transposase
p2862_p5_36	*orf36*	32172	33698	hypothetical protein	helix-turn-helix domain-containing protein [*Carnobacterium maltaromaticum* LMA28]	98	50	C	BglG super family	Transcriptional antiterminator
p2862_p5_37	*orf37*	33854	34291	hypothetical protein	ISL3 family transposase [*Carnobacterium maltaromaticum* LMA28]	100	93	C	transpos_IS3 super family	IS3 family transposase
p2862_p5_38	*orf38*	34324	34665	hypothetical protein	transposase (fragment) [*Carnobacterium maltaromaticum* LMA28]	92	96	C	transpos_IS3 super family	IS3 family transposase
p2862_p5_39	*itsB*	34945	35685	IstB-like ATP-binding protein	IS21-like element helper ATPase IstB [*Carnobacterium maltaromaticum* LMA28]	89	95	M	IS21_help_AAA	IS21-like element helper ATPase IstB
p2862_p5_40	*orf40*	35743	35961	transposase	transposase [*Carnobacterium inhibens* WN1359]	100	70	C	transpos_IS3 super family	IS3 family transposase
p2862_p5_41	*orf41*	35987	36670	transposase	helix-turn-helix domain-containing protein [*Carnobacterium maltaromaticum* LMA28]	50	85	C	transpos_IS3 super family	IS3 family transposase

* Abbreviations used in protein localization prediction: C- cytoplasm; M- membrane, E- extracellular

S1m. Annotation of the 2862_p5 plasmid of *C. maltaromaticum* strain 2862 (continued)

Tag	Gene	Position		Function	BLASTp best result	Identity (%)	Coverage (%)	Localization	Protein domain	Protein domain function
		start	stop							
p2862_p5_42	*orf42*	36862	37128	transposase	transposase [*Carnobacterium inhibens* WN1359]	100	76	C	transpos_IS3 super family	IS3 family transposase
p2862_p5_43	*orf43*	37392	37658	transposase	transposase [*Carnobacterium inhibens* WN1359]	100	76	C	transpos_IS3 super family	IS3 family transposase
p2862_p5_44	*orf44*	37850	38533	transposase	IS3 family transposase [*Carnobacterium inhibens* WN1359]	99	69	C	transpos_IS3 super family	IS3 family transposase
p2862_p5_45	*orf45*	38573	38812	hypothetical protein	hypothetical protein [*Carnobacterium maltaromaticum* LMA28]	98	83	E	no data	no data
p2862_p5_46	*orf46*	39307	39927	transposase	conserved hypothetical protein [*Carnobacterium maltaromaticum* LMA28]	100	98	C	transpos_IS3 super family	IS3 family transposase
p2862_p5_47	*orf47*	40124	40390	transposase	transposase [*Carnobacterium maltaromaticum* LMA28]	100	93	C	transpos_IS3 super family	IS3 family transposase
p2862_p5_48	*marR*	40627	41046	MarR family transcriptional regulator	MarR family transcriptional regulator [*Enterococcus* sp. 10A9_DIV0425]	100	65	C	HTH super family	Helix-turn-helix DNA binding domain
p2862_p5_49	*nmrA*	41049	41912	NmrA family transcriptional regulator	NmrA family NAD(P)-binding protein [*Enterococcus* sp. 10A9_DIV0425]	99	80	M	TMR_SDR_a	triphenylmethane reductase (TMR)-like proteins
p2862_p5_50	*orf50*	41976	43244	hypothetical protein	transposase [*Carnobacterium maltaromaticum* LMA28]	96	92	C	transpos_ISL3 super family	IS3 family transposase
p2862_p5_51	*orf51*	43315	43749	hypothetical protein	hypothetical protein [*Carnobacterium maltaromaticum* LMA28]	96	95	C	transpos_IS3 super family	IS3 family transposase

* Abbreviations used in protein localization prediction: C- cytoplasm; M- membrane, E- extracellular

S1m. Annotation of the 2862_p5 plasmid of *C. maltaromaticum* strain 2862 (continued)

Tag	Gene	Position start	Position stop	Function	BLASTp best result	Identity (%)	Coverage (%)	Localization	Protein domain	Protein domain function
p2862_p5_52	*orf52*	43906	44349	death-on-curing family protein	type II toxin-antitoxin system death-on-curing family toxin [*Enterococcus* sp. 669A]	99	55	M	Doc super family	Prophage maintenance system killer protein
p2862_p5_53	*mazE*	44368	44607	MazE antitoxin system	toxin [*Carnobacterium maltaromaticum* LMA28]	88	61	C	MazE_antitoxin super family	Antidote-toxin recognition MazE, bacterial antitoxin
p2862_p5_54	*orf54*	44879	45382	hypothetical protein	hypothetical protein CMA01_25390 [*Carnobacterium maltaromaticum* LMA28]	85	92	C	DDE_Tnp_IS240 super family	DDE domain, transposase
p2862_p5_55	*orf55*	45536	45802	hypothetical protein	transposase [*Carnobacterium inhibens* WN1359]	100	76	C	transpos_ISL3 super family	IS3 family transposase
p2862_p5_56	*orf56*	45994	46677	transposase	IS3 family transposase [*Carnobacterium inhibens* WN1359]	99	63	M	transpos_IS3 super family	IS3 family transposase
p2862_p5_57	*orf57*	46798	47064	hypothetical protein	transposase [*Carnobacterium inhibens* WN1359]	100	76	C	transpos_IS3 super family	IS3 family transposase
p2862_p5_58	*orf58*	47256	47939	transposase	IS3 family transposase [*Carnobacterium inhibens* WN1359]	99	69	C	transpos_IS3 super family	IS3 family transposase
p2862_p5_59	*orf59*	47984	48136	hypothetical protein	hypothetical protein [*Carnobacterium inhibens* WN1359]	77	72	E	no data	no data
p2862_p5_60	*orf60*	48129	49511	hypothetical protein	O-antigen ligase family protein [*Carnobacterium maltaromaticum* LMA28]	96	35	M	DUF4271 super family	Domain of unknown function

* Abbreviations used in protein localization prediction: C- cytoplasm; M- membrane, E- extracellular

S1m. Annotation of the 2862_p5 plasmid of *C. maltaromaticum* strain 2862 (continued)

Tag	Gene	Position		Function	BLASTp best result	Identity (%)	Coverage (%)	Localization	Protein domain	Protein domain function
		start	stop							
p2862_p5_61	*wecB*	49530	50654	UDP-N-acetyl-glucosamine 2-epimerase	UDP-N-acetylglucosamine 2-epimerase [*Carnobacterium maltaromaticum* LMA28]	100	88	C	GTB-type super family	glycosyltransferase family 1
p2862_p5_62	*capE*	50641	51765	capsular polysaccharide biosynthesis protein CapE	NAD-dependent epimerase/dehydratase family protein [*Carnobacterium maltaromaticum* LMA28]	98	77	C	NADB_Rossmann super family	Rossmann-fold NAD(P)(+)-binding proteins
p2862_p5_63	*capE*	51770	52789	NAD-dependent epimerase/dehydratase family protein	polysaccharide epimerase [*Carnobacterium* sp. CS13]	99	88	C	FlaA1 super family	NDP-sugar epimerase
p2862_p5_64	*orf64*	52815	53999	hypothetical protein	glycosyltransferase [*Carnobacterium inhibens* WN1359]	100	45	E	GT4_PimA-like	phosphatidyl-myo-inositol mannosyltransferase
p2862_p5_65	*ugd*	54014	55294	UDP-glucose 6-dehydrogenase	sugar dehydrogenase [*Carnobacterium inhibens* WN1359]	100	71	C	PRK15182 super family	Vi polysaccharide biosynthesis UDP-N-acetylglucosamine C-6 dehydrogenase TviB
p2862_p5_66	*galE*	55287	56321	UDP-glucose 4-epimerase	epimerase [*Carnobacterium inhibens* WN1359]	100	76	C	UDP_GE_SDE_e	UDP glucuronic acid epimerase
p2862_p5_67	*orf67*	56551	56817	hypothetical protein	transposase [*Carnobacterium inhibens* WN1359]	99	69	C	transpos_ISL3 super family	IS3 family transposase
p2862_p5_68	*orf68*	57009	57692	transposase	DUF308 domain-containing protein [*Carnobacterium maltaromaticum* LMA28]	100	93	C	HdeD super family	Uncharacterized membrane protein HdeD
p2862_p5_69	*orf69*	57849	58379	hypothetical protein	DUF5067 domain-containing protein [*Carnobacterium maltaromaticum* LMA28]	100	82	M	DUF4352 super family	Domain of unknown function
p2862_p5_70	*yitT*	58964	59407	membrane protein YitT	YitT family protein [*Carnobacterium maltaromaticum* LMA28]	100	93	M	DUF6198 super family	Family of unknown function

* Abbreviations used in protein localization prediction: C- cytoplasm; M- membrane, E- extracellular

S1m. Annotation of the 2862_p5 plasmid of *C. maltaromaticum* strain 2862 (continued)

Tag	Gene	Position start	Position stop	Function	BLASTp best result	Identity (%)	Coverage (%)	Localization	Protein domain	Protein domain function
p2862_p5_71	*orf71*	59718	60308	hypothetical protein	hypothetical protein [*Carnobacterium inhibens* WN1359]	100	55	M	PRiA4_ORF3 super family	Plasmid pRiA4b ORF-3-like protein
p2862_p5_72	*orf72*	60359	61123	hypothetical protein	IS3 family transposase [*Carnobacterium inhibens* WN1359]	98	65	C	transpos_IS3 super family	IS3 family transposase
p2862_p5_73	*orf73*	61442	62272	transposase	IS3 family transposase [*Carnobacterium inhibens* WN1359]	100	76	C	transpos_IS3 super family	IS3 family transposase
p2862_p5_74	*orf74*	62278	62568	transposase	transposase [*Carnobacterium maltaromaticum* LMA28]	98	65	C	Glyco_hydro super family	Glycosyl hydrolases
p2862_p5_75	*orf75*	62855	63028	hypothetical protein	amidohydrolase [*Carnobacterium maltaromaticum* LMA28]	100	100	M	Zinc_peptidase_like super family	Zinc peptidase
p2862_p5_76	*yjiB*	63220	64392	amidohydrolase	hydrolase [*Carnobacterium maltaromaticum* LMA28]	100	97	C	Glyco_hydro super family	Glycosyl hydrolases
p2862_p5_77	*orf77*	64385	64681	hypothetical protein	conserved hypothetical protein [*Carnobacterium maltaromaticum* LMA28]	100	96	E	transpos_IS3 super family	IS3 family transposase
p2862_p5_78	*parB*	64882	65502	partition protein B	partition protein B [*Carnobacterium maltaromaticum* LMA28]	100	92	C	HTH super family	Helix-turn-helix domains
p2862_p5_79	*orf79*	65700	65966	transposase	transposase [*Carnobacterium maltaromaticum* LMA28]	99	83	C	PTZ00121 super family	MAEBL; Provisional
p2862_p5_80	*orf80*	66152	68608	hypothetical protein	transposase [*Carnobacterium inhibens* WN1359]	100	76	C	transpos_IS3 super family	IS3 family transposase

* Abbreviations used in protein localization prediction: C- cytoplasm; M- membrane, E- extracellular

S1m. Annotation of the 2862_p5 plasmid of *C. maltaromaticum* strain 2862 (continued)

Tag	Gene	Position start	Position stop	Function	BLASTp best result	Identity (%)	Cover (%)	Localization	Protein domain	Protein domain function
p2862_p5_81	*orf81*	68861	69127	transposase	IS3 family transposase [*Carnobacterium inhibens* WN1359]	99	69	E	transpos_IS3 super family	IS3 family transposase
p2862_p5_82	*orf82*	69319	70002	transposase	hypothetical protein [*Carnobacterium maltaromaticum* LMA28]	95	76	E	no data	no data
p2862_p5_83	*parE*	70028	70432	partition protein E	Txe/YoeB family addiction module toxin [*Carnobacterium maltaromaticum* LMA28]	100	96	M	ParE_toxin super family	ParE toxin of type II toxin-antitoxin system, parDE
p2862_p5_84	*yoeB*	70459	70716	Txe/YoeB family addiction module toxin	type II toxin-antitoxin system Phd/YefM family antitoxin [*Carnobacterium maltaromaticum* LMA28]	100	97	C	PhdYeFM_antitox super family	Antitoxin Phd_YefM, type II toxin-antitoxin system
p2862_p5_85	*orf85*	70709	70972	prevent-host-death protein	DUF6290 family protein [*Carnobacterium maltaromaticum* LMA28]	80	52	E	Hol_Tox super family	Putative Holin-like Toxin
p2862_p5_86	*orf86*	71045	71233	hypothetical protein	putative holin-like toxin [*Carnobacterium maltaromaticum* LMA28]	95	94	E	Hol_Tox super family	Putative Holin-like Toxin
p2862_p5_87	*orf87*	71417	71566	hypothetical protein	type IV secretory system conjugative DNA transfer family protein [*Carnobacterium maltaromaticum* LMA28]	100	100	C	HTH super family	DNA binding domain
p2862_p5_88	*orf88*	71670	71801	hypothetical protein	IS3 family transposase [*Enterococcus faecalis*]	100	81	C	transpos_IS3 super family	IS3 family transposase
p2862_p5_89	*orf89*	71859	72092	hypothetical protein	transposase [*Carnobacterium inhibens* WN1359]	100	76	C	transpos_IS3 super family	IS3 family transposase

* Abbreviations used in protein localization prediction: C- cytoplasm; M- membrane, E- extracellular

S1m. Annotation of the 2862_p5 plasmid of *C. maltaromaticum* strain 2862 (continued)

Tag	Gene	Position start	Position stop	Function	BLASTp best result	Identity (%)	Coverage (%)	Localization	Protein domain	Protein domain function
p2862_p5_90	*orf90*	72205	72471	hypothetical protein	transposase [*Carnobacterium inhibens* WN1359]	100	76	C	transpos_IS3 super family	IS3 family transposase
p2862_p5_91	*orf91*	72648	72914	hypothetical protein	IS3 family transposase [*Carnobacterium inhibens* WN1359]	99	63	C	transpos_IS3 super family	IS3 family transposase
p2862_p5_92	*orf92*	73106	73789	transposase	IS3 family transposase [*Carnobacterium inhibens* WN1359]	100	73	C	transpos_IS3 super family	IS3 family transposase
p2862_p5_93	*orf93*	73850	73987	hypothetical protein	IS3 family transposase [*Carnobacterium inhibens* WN1359]	99	64	C	transpos_IS3 super family	IS3 family transposase
p2862_p5_94	*orf94*	74123	74536	hypothetical protein	IS3 family transposase [*Carnobacterium inhibens* WN1359]	93	78	C	transpos_IS3 super family	IS3 family transposase
p2862_p5_95	*orf95*	74581	74769	hypothetical protein	recombinase family protein [*Carnobacterium maltaromaticum* LMA28]	97	93	C	HTH super family	DNA binding domain
p2862_p5_96	*tnpR*	74819	75412	transposase	Tn3 family transposase [*Carnobacterium maltaromaticum* LMA28]	100	92	C	DUF4158 super family	Domain of unknown function (DUF4158)
p2862_p5_97	*orf97*	75601	76440	hypothetical protein	Tn3 family transposase [*Carnobacterium maltaromaticum* LMA28]	100	96	M	DDE_Tnp_Tn3 super family	Tn3 transposase DDE domain
p2862_p5_98	*orf98*	76563	78602	hypothetical protein	hypothetical protein [*Carnobacterium maltaromaticum* LMA28]	100	98	M	no data	no data
p2862_p5_99	*orf99*	78902	79504	hypothetical protein	hypothetical protein [*Carnobacterium maltaromaticum* LMA28]	76	60	M	no data	no data

* Abbreviations used in protein localization prediction: C- cytoplasm; M- membrane, E- extracellular

Table S2. Bacterial strains and primers used this study

Bacterial strains				
**Species**	**Strain**	**Isolation source**	**Optimum growth conditions**	**Source (Reference) ***
Carnobacterium alterfunditum	2835	Cryoconite, Antarctica	50% BHI, 16°C	this study
Carnobacterium spp.	2850	Ornitoghenic soil, Antarctica	50% BHI, 16°C	this study
Carnobacterium spp.	2851	Ornitoghenic soil, Antarctica	50% BHI, 16°C	this study
Carnobacterium jeotgali	2854	Soil, Baranowski Glacier foreland, Antarctica	50% BHI, 16°C	this study
Carnobacterium spp.	2855	Soil, Baranowski Glacier foreland, Antarctica	50% BHI, 16°C	this study
Carnobacterium spp.	2856	Soil, seal elephants wake, Antarctica	50% BHI, 16°C	this study
Carnobacterium maltaromaticum	2857	Soil, Hans Glacier foreland, Spitsbergen, Arctic	50% BHI, 16°C	this study
Carnobacterium jeotgali	2858	Soil, Windy Glacier foreland, Antarctica	50% BHI, 16°C	this study
Carnobacterium spp.	2859	Soil, Windy Glacier foreland, Antarctica	50% BHI, 16°C	this study
Carnobacterium maltaromaticum	2862	Microbial mats, Jasnorzewski Garden, Antarctica	50% BHI, 16°C	this study
Carnobacterium alterfunditum	DSM5972	Polar lake sediment, Antarctica	50% BHI, 16°C	[4]
Carnobacterium funditum	DSM5970	Polar lake sediment, Antarctica	50% BHI, 16°C	[4]
Carnobacterium maltaromaticum	IBB3447	Cow raw milk, Poland	100% BHI, 30°C	[82]
Bacillus subtilis	168	Human skin	100% BHI, 37°C	[81]
Campylobacter jejuni	81176	Food sample	100% BHI, 37°C	[75]
Candida albicans	CAI-4	Human intravenous infection	100% BHI, 37°C	[37]
Lactococcus lactis	IL1403	Plasmid-free derivative of cheese isolate	100% BHI, 30°C	[48]
Listeria monocytogenes	LMGT2604	Food sample	100% BHI, 37°C	[75]
Pseudomonas aeruginosa	ATCC9027	Human outer ear infection	100% BHI, 37°C	FB UW
Salmonella typhimurium	TT622	pork sausage	100% BHI, 37°C	[81]
Staphylococcus aureus	ATCC6538	Human skin lesion	100% BHI, 37°C	FB UW
Staphylococcus intermedius	DSM20373	Human skin lesion	100% BHI, 37°C	DSMZ
Staphylococcus pseudintermedius	DMS21284	Human skin lesion	100% BHI, 37°C	DSMZ
Staphylococcus hyicus subsp. *chromogenes*	DMS20454	Human epidermitis	100% BHI, 37°C	DSMZ

* Abbreviations used in protein localization prediction: C- cytoplasm; M- membrane, E- extracellular

Staphylococcus hyicus	DMS20459	Human skin lesion	100% BHI, 37°C	DSMZ
Staphylococcus epidermidis	DMS20044	Human skin infection	100% BHI, 37°C	DSMZ
Staphylococcus hyicus	DMS17421	Human nose infection	100% BHI, 37°C	DSMZ
Staphylococcus caprae	DMS20608	Human intravenous infection	100% BHI, 37°C	DSMZ
Staphylococcus lugdunensis	DMS4804	Human skin lesion	100% BHI, 37°C	DSMZ
Staphylococcus schleiferi subsp. *coagulans*	DMS6628	Human skin lesion	100% BHI, 37°C	DSMZ
Staphylococcus saprophyticus	DMS18669	Human skin lesion	100% BHI, 37°C	DSMZ
Staphylococcus delphini	DMS20771	Human throat infection	100% BHI, 37°C	DSMZ
Staphylococcus saprophyticus	DMS20229	Human throat infection	100% BHI, 37°C	DSMZ
Streptococcus agalactiae	IBB123	Cow raw milk	100% BHI, 37°C	IBB PAS
Streptococcus sobrinus	IBB3450	Human throat infection	100% BHI, 37°C	IBB PAS
Streptococcus pneumoniae	5005	Human infection	100% BHI, 37°C	IBB PAS
Sterpococcus parauberis	IBB272	Cow raw milk	100% BHI, 37°C	IBB PAS

Primers

**Primer**	**PCR product size (bp)**	**Sequence (5'-3') ****	**Target gene**	**Primer coordinates (bp)**
27F<br>1492R	1466	AGAGTTTGATCCTGGCTCAG<br>GGCTACCTTGTTACGACTT	16S rRNA	8–27<br>1492–1513
rpoA-21-F<br>*rpoA*-23-R	802	TGATYGARTTTGAAAAA<br>CHGTRTTRATDCCDGCR	*rpoA*	1-802
pheS-21-F<br>*pheS*-22-R	475	AYCCNGCHCGYGAYATG<br>CWARVCCRAARGCAAARC	*pheS*	557-1031
atpA-20-F<br>*atpA*-27-R	1123	TAYRTYGGKGAYGGDATYGC<br>CCRCGRTTHARYTTHGCYTG	*atpA*	97-1219
KIL1<br>GIL2	300-1500	CAAGGCATCCACCGT<br>GAAGTCGTAACAAGG	conserved regions in the 16S and 23S rRNA genes	

* **DSMZ-** Deutsche Sammlung von Mikroorganismen und Zellkulturen, ACAM- Australian Collection of Antarctic Microorganisms, University of Tasmania, Box 252C, Hobart, Tasmania 7001, Australia; **IBB PAS**- Institute of Biochemistry and Biophysics strains collection, Polish Academy of Sciences, Pawinskiego 5a, 02-106 Warsaw, Poland**; FB UW** - Faculty of Biology, University of Warsaw, Poland

** R=A or G; Y=T or C; W=A or T; H=A or T or C; D=G or A or T; K=G or T

* Abbreviations used in protein localization prediction: C- cytoplasm; M- membrane, E- extracellular

Table S3. Accession numbers of 16S rRNA, *pheS* and *rpoA* genes from polar *Carnobacterium* sp. strains deposited in the GenBank (NCBI) database

Strain		Nucleotide sequence		
		16S rRNA	*pheS*	*rpoA*
C. alterfunditum	2835	OQ266887	OQ865257	OQ865241
C. jeotgali	2854	OQ445549	OQ865250	OQ865246
C. maltaromaticum	2857	OQ445550	OQ865256	OQ865238
	2862	OQ445552	OQ865255	OQ865247
Carnobacterium spp.	2850	OQ448831	OQ865254	OQ865242
	2851	OQ445553	OQ865253	OQ865240
	2855	OQ445555	OQ865259	OQ865243
	2856	OQ445554	OQ865252	OQ865239
	2858	OQ445556	OQ865258	OQ865244
	2859	OQ445557	OQ865251	OQ865245

* Abbreviations used in protein localization prediction: C- cytoplasm; M- membrane, E- extracellular
